# Conformational Restriction of Designer Drugs Reveals Subtype-Selective and Biased CB_2_ Agonists with Neuroprotective Effects

**DOI:** 10.1021/acs.jmedchem.5c00604

**Published:** 2025-08-12

**Authors:** Claudia Gioé-Gallo, Sandra Ortigueira, Rubén Prieto-Díaz, Marialessandra Contino, Jhonny Azuaje, Maria Grazia Perrone, Chiara Riganti, Domenico Alberga, Giuseppe Felice Mangiatordi, Antonio Andújar-Arias, Aitor García-Rey, Giovanni Graziano, Angela Stefanachi, Cristina Val, Antón Leandro Martínez, Joan Biel Rebassa, David Reza, Asier Selas, Fabio Francavilla, M. Rita Paleo, Xerardo García-Mera, M. Isabel Loza, Gemma Navarro, José Brea, Eddy Sotelo

**Affiliations:** † Centro Singular de Investigación en Química Biolóxica y Materiais Moleculares (CIQUS), 16780 Universidade de Santiago de Compostela, 15782 Santiago de Compostela, Spain; ‡ Dipartimento di Farmacia-Scienze del Farmaco, 9295 Università degli Studi di Bari ALDO MORO, via Orabona 4, 70125 Bari, Italy; § Dipartimento di Oncologia, 9314 Università degli Studi di Torino, piazza Nizza 44, 10126 Torino, Italy; ∥ CNR − Institute of Crystallography, Via Giovanni Amendola, 122/O, 70126 Bari, Italy; ⊥ Centro Singular de Investigación en Medicina Molecular y Enfermedades Crónicas (CIMUS), Universidade de Santiago de Compostela, 15782 Santiago de Compostela, Spain; # Department of Biochemistry and Physiology, School of Pharmacy and Food Science, Universitat de Barcelona, Institut de Neurosciències UB, Barcelona 08028, Spain

## Abstract

This study presents the design, synthesis, and characterization of a novel series of structurally simple, selective, and functionally biased CB_2_ receptor (CB_2_R) agonists with potent anti-inflammatory and neuroprotective properties. These compounds were developed using a conformational restriction strategy to abolish CB_1_R binding, thereby enhancing CB_2_R selectivity. Pharmacological profiling identified ligands with distinct bias toward β-arrestin, MAPK, and G-protein signaling pathways. The series exhibits favorable drug-like properties, including high BBB permeability, low P-glycoprotein interaction, and microsomal stability. Representative compounds demonstrated neuroprotective activity in mouse primary neuronal assays and significantly reduced ROS and caspase levels *in vitro*, indicating mitigation of oxidative stress and apoptosis. In a neuron-like SH-SY5Y model expressing pathogenic mutations, they preserved neurite complexity in a CB_2_R-dependent manner. Collectively, these findings highlight the advantages of conformational restriction in transforming abused promiscuous, neurotoxic ligands into highly selective and efficacious agents for the treatment of neurodegenerative disorders, without CB_1_R-mediated psychoactive effects.

## Graphical abstract

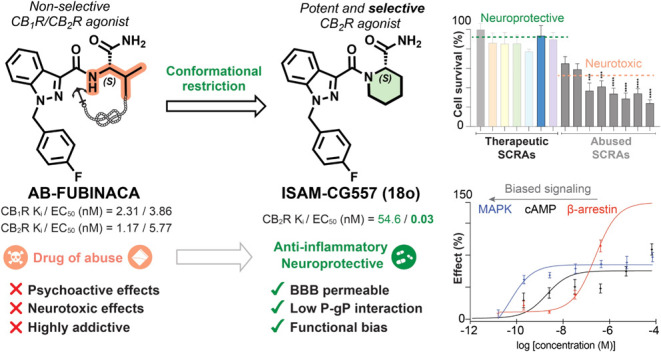

## Introduction

The endocannabinoid system (ECS) is a crucial regulatory network responsible for maintaining homeostasis in diverse physiological processes within the human body.^1,2^ The ECS comprises endogenous cannabinoids, cannabinoid receptors (CBRs: CB_1_R and CB_2_R), and approximately 20 associated enzymes involved in the biosynthesis and degradation of endocannabinoids, as well as hundreds of lipid mediators. It modulates a wide range of physiological functions, including appetite, pain sensation, immune responses, mood, and memory.^3,4^ CB_1_R and CB_2_R, both class A GPCRs with high transmembrane sequence similarity,^5,6^ have distinct distributions and physiological roles, making them key regulators of the endocannabinoid system.^7–9^ Since the discovery and cloning of CBRs, interest in understanding their signaling mechanisms, pharmacology, and therapeutic potential has grown significantly.^10,11^

The CB_2_R, initially dubbed as the peripheral cannabinoid receptor, due to its early discovery and abundance in immune system cells, plays a crucial role in regulating immune response and inflammation.^5,9,12^ More recently, high levels of CB_2_R expression have been confirmed in different brain regions, including the cortex, striatum, amygdala, cerebellum, and microglia.^13,14^ Notably, under conditions of inflammation or glial activation, CB_2_R expression undergoes a significant upregulation. This widespread expression pattern suggests the involvement of CB_2_R in a multitude of neurological functions within the brain.^9,12^ Emerging evidence supports a critical role for CB_2_R in mediating neuroprotection and neuroinflammation.^15,16^ Dysregulation of the CB_2_R produces immunomodulatory effects, influencing cytokine release, cell migration, and immune cell proliferation and, has been implicated in various pathological conditions, highlighting its involvement in chronic inflammation and autoimmune disorders.^12,14^

The CB_2_R′s involvement in neuronal damage and neuroinflammation underscores its potential as a therapeutic target for neuroprotection and the management of central nervous system pathologies with an inflammatory basis.^17^ Indeed, by modulating the activity of microglia, the primary immune cells in the brain, CB_2_R agonists can suppress the production of pro-inflammatory cytokines and enhance the clearance of toxic protein aggregates implicated in neurodegenerative processes, promoting neurogenesis and neuroplasticity, facilitating neuronal repair and regeneration.^18–20^ Additionally, preclinical data and clinical studies have demonstrated the efficacy of CB_2_R agonists in ameliorating autoimmune responses, reducing inflammation, and alleviating chronic pain without the psychoactive effects associated with CB_1_R receptor activation.^21^ Despite their promise, CB_2_R agonists have not performed well in clinical trials.^9,22^ Several factors may account for the discrepancy between preclinical and clinical efficacy, including interspecies differences in receptors and signaling pathways, insufficient receptor subtype selectivity and issues with functional behavior. Consequently, there is a pressing need to develop novel potent, subtype selective and effective CB_2_R agonists to address challenging medical needs.^12,23,24^

Indole and indazole derivatives are among the earliest identified and most studied synthetic CBR ligands.^25^ While these ligands inspired the early development of CBR agonists, a significant number of derivatives based on these scaffolds have emerged as designer drugs of abuse since the 2000s, under the misleading claim of being synthetic, legal and safe alternatives to marijuana (Figure 1).^25,26^ In addition to their promiscuous effects on CB_1_R and CB_2_R, abused synthetic cannabinoid receptor agonists (SCRAs) demonstrate exceptional potency and efficacy (Figure 1), making them archetypal models for developing pharmacomodulation strategies to identify CB_2_R-selective agonists.^27,28^

**Figure 1:**
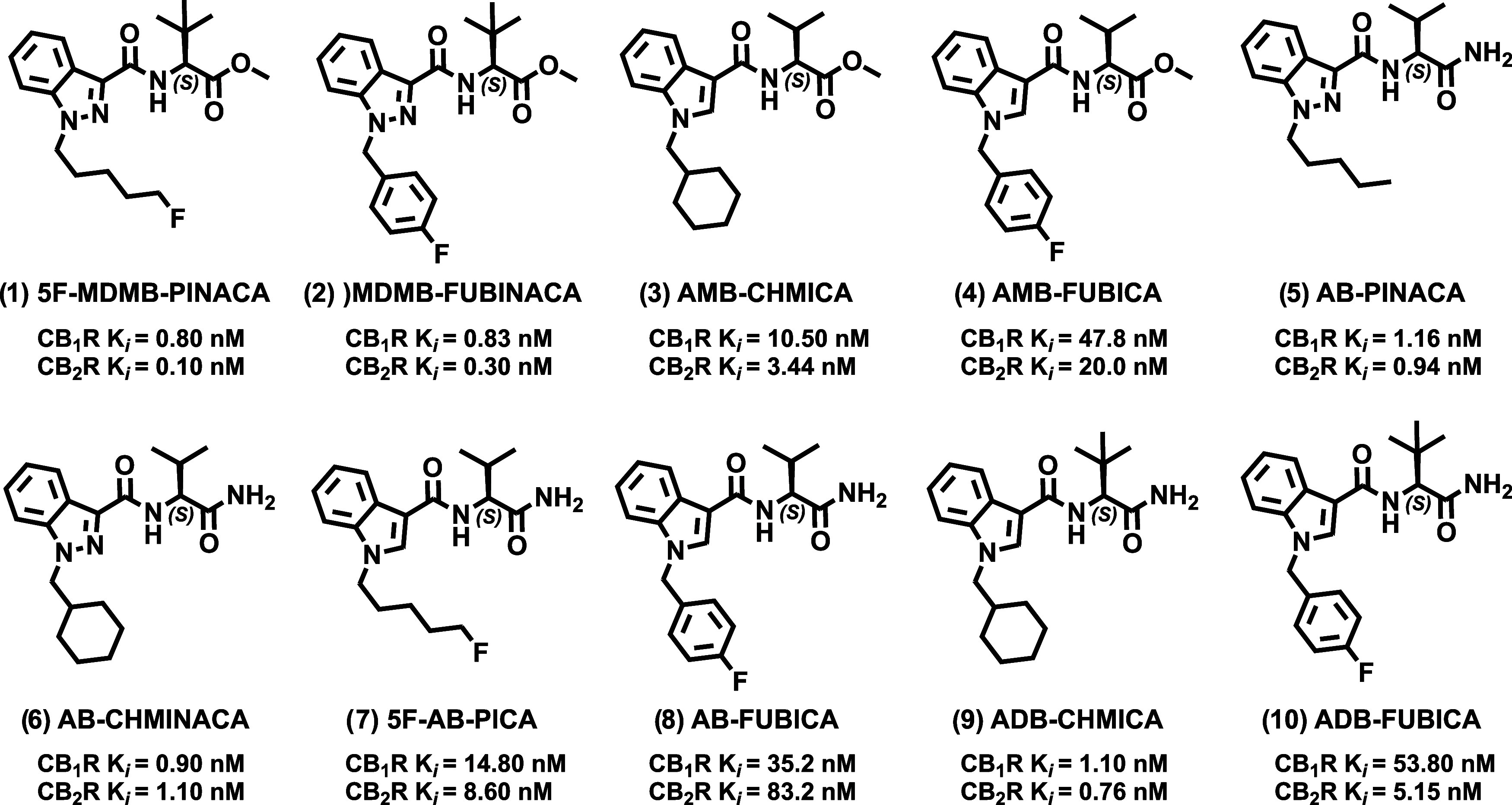
Structure and binding data of representative abused indole and indazole SCRAs used as models in this study.^29^

Recent reports have highlighted medicinal chemistry efforts aimed at dissociating the intrinsically promiscuous cannabinomimetic profiles of designer drugs to develop selective CB_2_R agonists.^30^ Despite important advances in structural biology of cannabinoid receptors,^7,8,10^ the high homology between these receptors continues to pose significant challenges, making this goal elusive. We recently reported the synthesis and detailed pharmacological characterization of a large collection of indole- and indazole-based abused SCRAs.^29^ This study revealed key structure–activity relationship trends that inspired the design of new series. Building upon these findings, we herein investigated the impact of introducing conformational constraints on prototypical indole- and indazole-based designer drugs of abuse (Figure 1). This conformational lock strategy effectively eliminated CB_1_R binding, resulting in the discovery of structurally novel, potent, efficacious, and highly selective CB_2_R agonists. The new ligands exhibit diverse functional selectivity profiles, excellent CNS permeability, and demonstrate remarkable anti-inflammatory and neuroprotective properties.

## Results and Discussion

### Design

Understanding the thermodynamics of molecular recognition events is essential in drug design.^31^ When a ligand binds to its receptor, it undergoes a conformational energy loss, which increases entropy in the surroundings, often weakening the interaction between the ligand and receptor.^32^ To mitigate these entropic penalties and enhance the potency of the ligand, a thoughtful strategy is to limit its conformational flexibility through rigidification.^33^ This method has become a well-established approach in rational drug design,^31^ as it can significantly improve both pharmacodynamic and pharmacokinetic profiles, leading to better clinical outcomes. By locking a ligand into a specific conformation optimal for binding, rigidification can improve affinity and selectivity profiles, thus reducing off-target interactions. In the development of kinase inhibitors, for example, scaffold rigidification enhances selectivity by reducing interactions with related kinases, minimizing adverse effects, and improving therapeutic efficacy.^34^ Similarly, in the design of HIV protease inhibitors, rigidification boosts metabolic stability and bioavailability by stabilizing the peptide backbone.^35^

The accepted pharmacophoric model of synthetic cannabinoid receptor agonists (SCRAs) comprises four key structural elements (Figure 2): the tail (purple), the heterocyclic core (green), the linker (blue), and the head (brown). Our early study on the pharmacological profiles of abused indole and indazole-based SCRAs^29^ revealed that methylation at the amide group near the heterocyclic core led to a significant reduction in cannabinoid receptor (CBR) affinity, compared to their unmethylated counterparts (Figure 2, compare the binding data for compounds **11** vs **12** and **13** vs **14**).^29^ This effect was significantly more pronounced for CB_1_R than CB_2_R (Figure 2), thus suggesting an opportunity to develop CB_2_R-selective agonists. This observation guided the design of herein documented conformationally restricted analogues (Figure 2), in which a five- or six-membered ring was created (Figure 2 bottom left, red dotted lines) to connect the nitrogen atom of the amide group in the linker fragment (Figure 2, blue) to the central carbon of the amino acid (Figure 2, Series **I**–**IV**). This structural modification, aimed at limiting the flexibility of the molecules, was expected to retain CB_2_R affinity while drastically reducing CB_1_R binding, thus improving the selectivity and therapeutic profile. In practice, the proposed strategy involved replacing the classical amino acids present in abused SCRAs (Figure 1, e.g., valine and *tert*-leucine) with proline and homoproline derivatives (Figure 2). It was hypothesized that the reduced rotational freedom conferred by this bridging would induce a conformational lock in the bioactive form of the ligands, thereby diminishing CB_1_R binding while preserving the critical interactions with the orthosteric pocket of CB_2_R.

**Figure 2:**
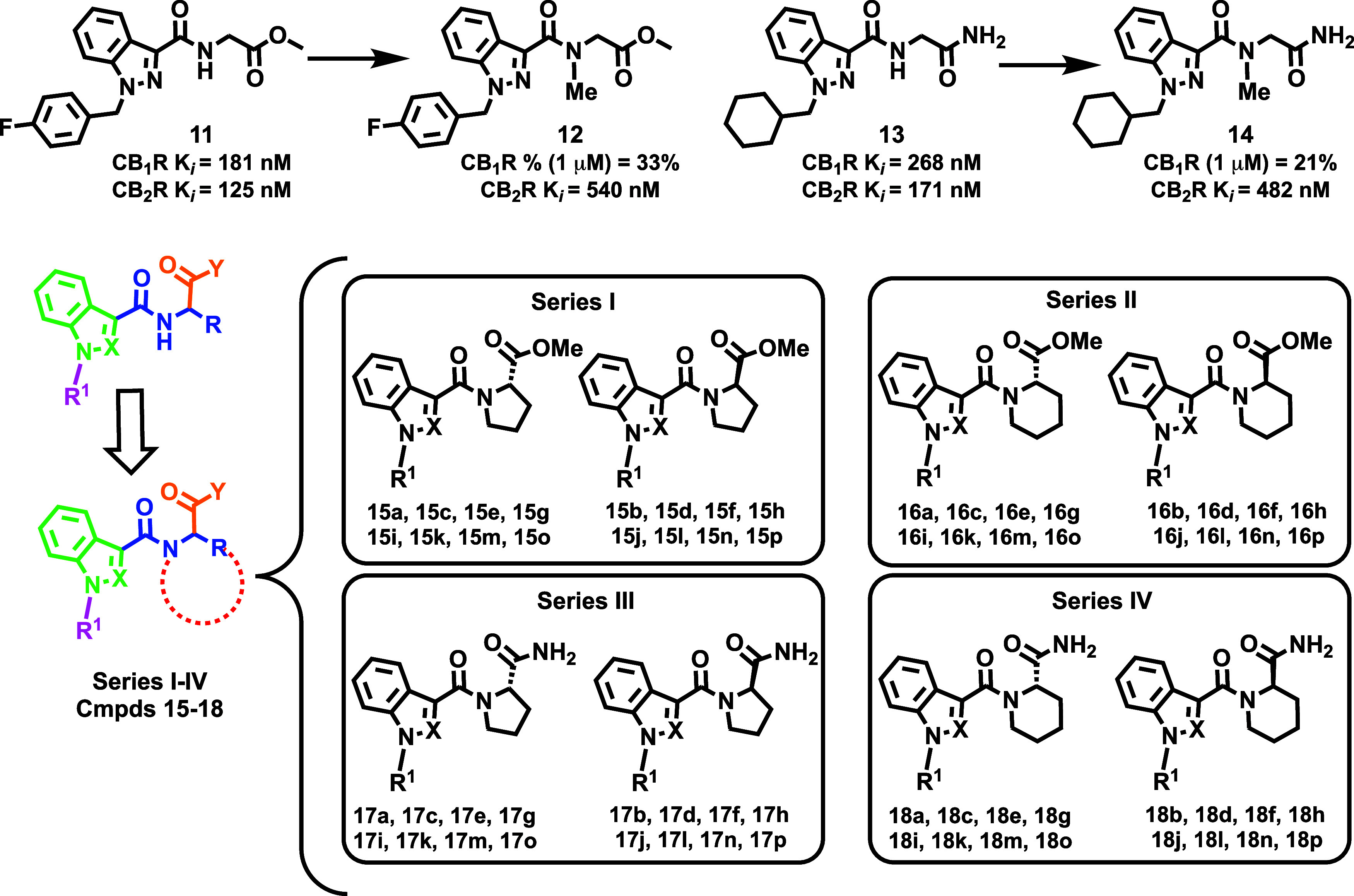
Design strategy followed within the project and general structure of the new series.

### Prediction and Comparative Analysis of Molecular Descriptors Impacting Drug-Like Properties

Assessing physicochemical properties early in drug discovery is crucial, as they influence ADME-Tox profiles.^36^ Molecular descriptors like Log *P*, Log *D*, and tPSA help predict a compound’s biological behavior, such as membrane permeability and blood-brain barrier (BBB) crossing.^37,38^ Cannabinoid receptor ligands often exhibit high lipophilicity, challenging drug-likeness criteria and pharmacokinetics.^39,40^ This high lipophilicity can contribute to adverse effects and pharmacokinetic challenges.^40^ To address this, we conducted an *in silico* evaluation of molecular descriptors for newly designed cyclic amides compared to abused SCRAs (Figure 3).^29^ This study was conducted during the library design phase to compare the new series with abused SCRAs and to anticipate issues that could affect the drug-likeness and ADME properties of the novel series. For this assessment, 64 representative compounds were selected from the abused SCRAs series [including the two central scaffolds (indole and indazole), four representative R^1^ (pentyl, 5-fluoropentyl, cyclohexylmethyl and 4-fluorobenzyl), and two R^2^ groups (isopropyl and *tert*-butyl), as well as their (*R*) and (*S*) enantiomers]. The same scaffolds and R^1^ groups were introduced to the new cyclic amides, substituting the R^2^ group with either a 5- or 6-membered ring.

**Figure 3:**
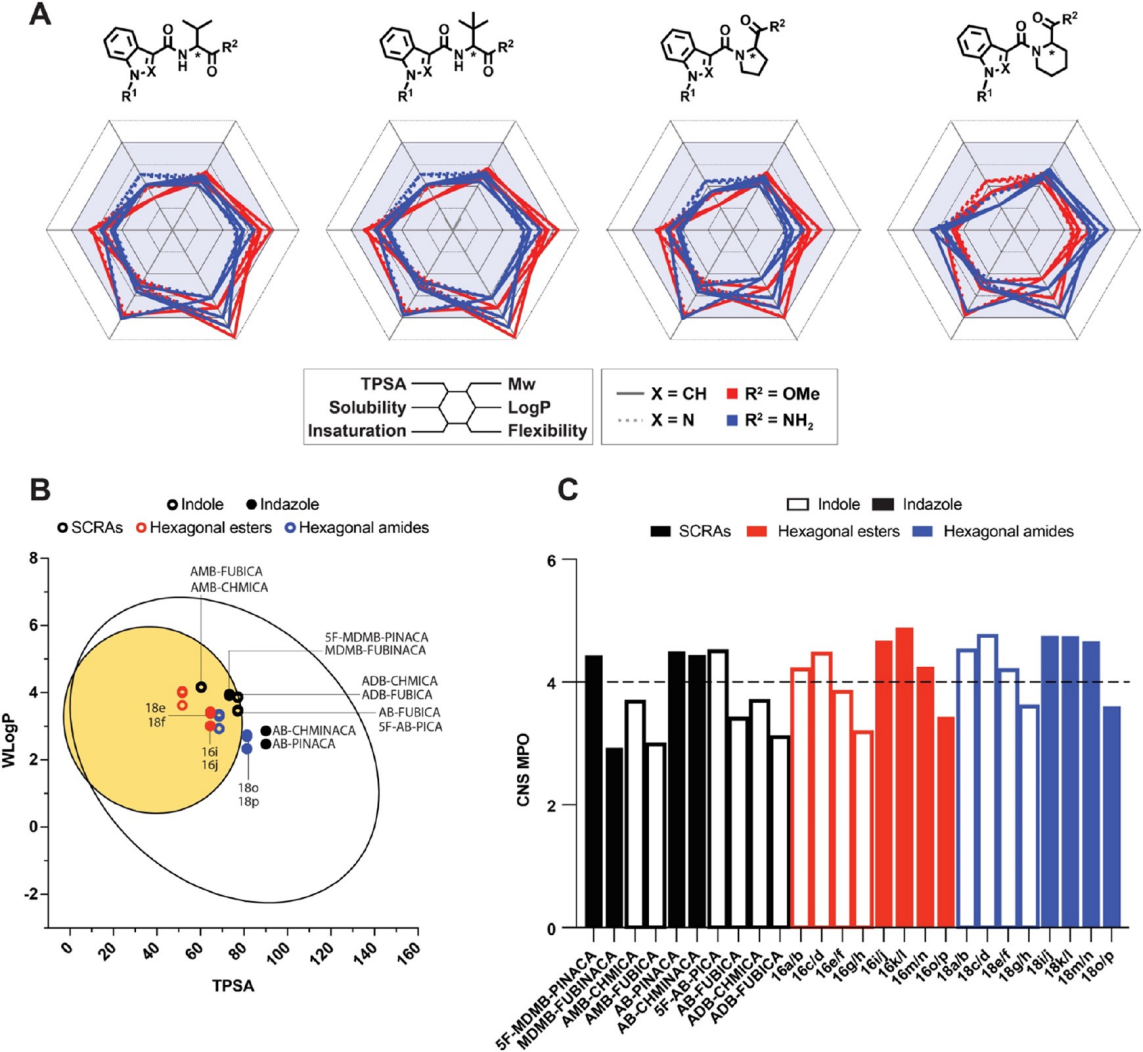
Comparative analysis of molecular descriptors. (A) Radar-type molecular descriptors analysis, (B) BOILED-egg, (c) CNS-MPO.

Six molecular descriptors were selected and calculated to identify key differences between the two series (Figure 3). The predicted Log *P* values of the conformationally restricted ligands (Figure 2, Compounds **15**–**18**) typically ranged from 2 to 3.5, which is 1.5 to 3 points lower than those calculated for abused SCRAs,^41^ remaining well within the range compatible with efficient blood-brain barrier (BBB) permeability. The results, presented in Figure 3A, demonstrate that the introduction of the cyclic structure (conformational restriction) leads to significant changes in representative molecular descriptors. As observed (Figure 3A), the new series show improved parameters, particularly with marked reductions in lipophilicity (Log *P*), polar surface area (TPSA) and flexibility (number of rotatable bonds).

To complement the predictive analysis of how structural rigidification would affects physicochemical properties of the new ligands (**15**–**18**) and its comparison with abused SCRAs (Figure 3), two additional computational approaches were employed: the CNS MPO criteria and the BOILED-egg model (Figure 3).^42,43^ A CNS-MPO score above 4 is generally considered a strong indicator of effective BBB permeability.^43^ The BOILED-egg model offers a simple yet effective method for predicting both blood-brain barrier (BBB) penetration and gastrointestinal absorption, improving early stage drug evaluation by classifying compounds based on their potential to reach the CNS or other target tissues.^42^ Finally, a screening was performed to identify any potential Pan-Assay Interference Structures (PAINS), using the PAINS filters,^44^ which could otherwise lead to false positives in biological assays. The studies were conducted for six representative new ligands (**16b**, **16j**, **18e**, **18f**, **18o**, and **18p**) and a selection of structural analogs of abused SCRAs. As observed (Figure 3), the computational predictions for CNS-MPO and BOILED-egg parameters yielded excellent results, suggesting that the conformationally restricted ligands possess favorable profiles as potential CNS-targeted therapeutics. The CNS-MPO scores (Figure 3C), generally above 4, suggest that new ligands are well-suited for crossing the blood-brain barrier (BBB) and maintaining efficacy within the CNS. Similarly, the BOILED-egg model (Figure 3B) supports these findings, with most ligands positioned in the yellow region, indicating favorable BBB permeability and optimal CNS exposure. Finally, all tested ligands passed the PAINS filter (see Experimental Section), ruling out the possibility of false positives in biological assays and suggesting they are robust drug candidates.

### Chemistry

Eight carboxylic acids (Scheme 1, Compounds **19a**–**h**), obtained via alkylation and saponification,^29^ were selected to systematically explore the effects of rigidification of the linker region (Figure 2) on the cannabinomimetic profile. Carboxylic acids **19** incorporate both indole and indazole cores and the most common tail residues (R^1^) found in abused SCRAs: *n*-pentyl, 5-fluoropentyl, cyclohexylmethyl, and 4-fluorobenzyl (Figure 2). Four cyclic amino acid methyl esters (Scheme 1, Compounds **20a**–**d**), with both (*R*) and (*S*) configurations, were chosen as precursors. The library was synthesized using an efficient two-step approach (Scheme 1). First, the amide coupling of compounds **19** with commercially available enantiopure methyl prolinates or homoprolinates (**20a**–**d**) yielded the targeted ester derivatives (**15** and **16**) with high efficiency (72 to 98%). In the second step, mild ammonolysis of **15** and **16** afforded the final amides (**17** and **18**) in nearly quantitative yields. The structure and diversity of the 64-member library are shown in Scheme 1.

**Scheme 1:**
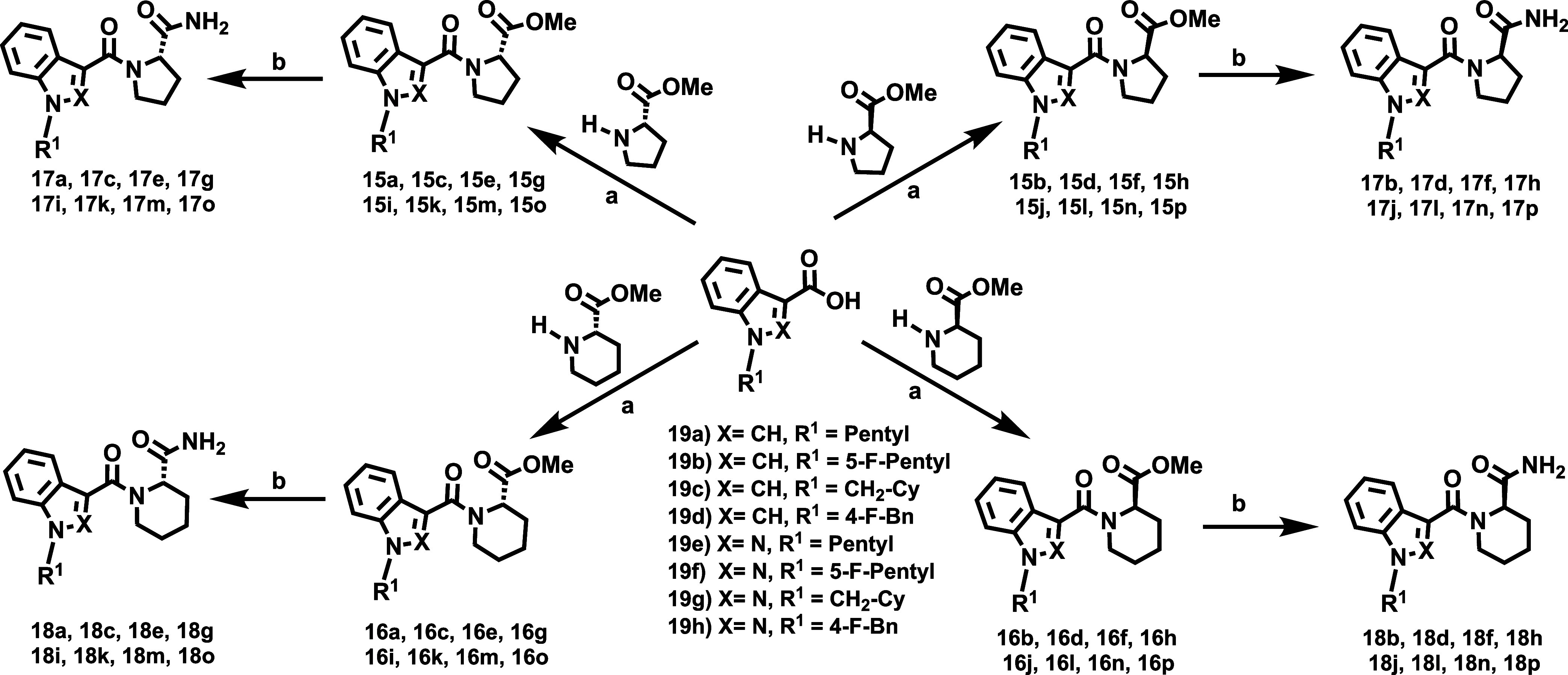
Synthetic Pathway Employed for the Synthesis of the New Series of CBR Ligands^a^

a Method (a) HATU, DIPEA, CH_2_Cl_2_, rt, 24 h. Method (b) NH_3_ in MeOH, rt, 4 h.

Although racemization is unlikely under the utilized experimental conditions,^45^ it was standard practice to analyze all library members using analytical chiral HPLC to verify the preservation of the configuration at the stereocenter (ee ≥ 98%) in the final compounds. A selection of the HPLC traces obtained for representative enantiomeric pairs and the characterization through Circular Dichroism (CD) is presented in Figure S1. A detailed description of the synthetic methods and the complete structural, spectroscopic, and analytical data for all compounds as well as HPLC traces for representative enantiomeric pairs is provided in the experimental part and Supporting Information.

### Biological Evaluation

The cannabimimetic binding profile of the 64 newly synthesized ligands was first studied *in vitro* by evaluating their affinity (*K*_*i*_) for human CB_1_R and CB_2_R following established experimental protocols.^29^ Competitive radioligand binding assays were conducted using membrane preparations from human CB_1_R transfected in CHO C3 cells and from human CB_2_R transfected in HEK-293T cells. A well-characterized tritiated ligand ([^3^H]­CP55,940) was used for these assays. The binding affinity data obtained are reported in Tables 1–4. For comparative purposes, binding data for representative CBR ligands (Surinabant, CP55,940, and GW405833) were also determined using the reported experimental protocols. These data are also included in the tables. Representative dose–response curves obtained for selected ligands during the binding affinity determinations at CB_2_R are presented in Figure S2. A detailed description of the employed protocols is reported in the experimental part and the Supporting Information.

**Table 1:**
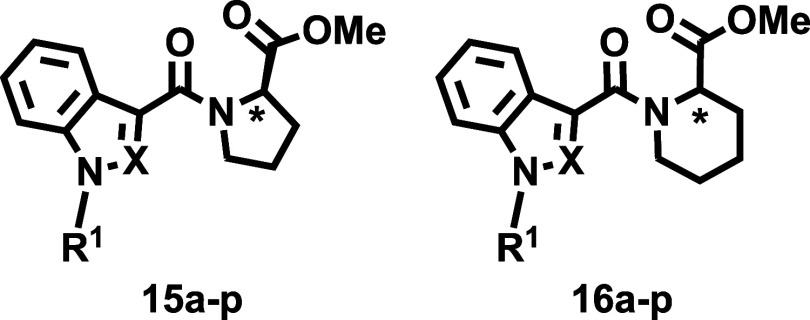
Structure and Binding Data Obtained for the Esters **15a**–**p** and **16a**–**p**

				CB_1_R		CB_2_R		
compound	X	*(*R*/*S*)	R^1^	*K*_*i*_ (nM) or % at 1 μM^a^	p*K*_*i*_	*K*_*i*_ (nM) or % at 1 μM^b^	p*K*_*i*_	SI *K*_*i*_ CB_1_R/ *K*_*i*_ CB_2_R
**15a**	CH	*(S)*	Pentyl	121 ± 6	6.92	189 ± 10	6.72	0.6
**15b**	CH	*(R)*	Pentyl	4%	-	25%	-	-
**15c**	CH	*(S)*	5-F-Pentyl	55.9 ± 2.7	7.25	71.7 ± 2.0	7.14	0.8
**15d**	CH	*(R)*	5-F-Pentyl	3%	-	26%	-	-
**15e**	CH	*(S)*	CH_2_–Cy	11.3 ± 0.5	7.95	38.3 ± 2.1	7.42	0.3
**15f**	CH	*(R)*	CH_2_–Cy	32%	-	463 ± 11	6.33	>100
**15g**	CH	*(S)*	4-F-Bn	14.4 ± 0.6	7.84	47.7 ± 1.9	7.32	0.3
**15h**	CH	*(R)*	4-F-Bn	10%	-	599 ± 13	6.22	>100
**15i**	N	*(S)*	Pentyl	337 ± 9	6.47	75.9 ± 2.2	7.12	4
**15j**	N	*(R)*	Pentyl	4%	-	208 ± 5	6.68	>100
**15k**	N	*(S)*	5-F-Pentyl	173 ± 5	6.76	41.9 ± 2.1	7.38	4
**15l**	N	*(R)*	5-F-Pentyl	1%	-	513 ± 3	6.29	>100
**15m**	N	*(S)*	CH_2_–Cy	73.4 ± 2.9	7.13	20.7 ± 0.6	7.68	3
**15n**	N	*(R)*	CH_2_–Cy	2%	-	99.3 ± 2.8	7.00	>100
**15o**	N	*(S)*	4-F-Bn	46.2 ± 2.3	7.34	16.5 ± 1.1	7.78	3
**15p**	N	*(R)*	4-F-Bn	5%	-	157 ± 4	6.80	>100
**16a**	CH	*(S)*	Pentyl	644 ± 13	6.19	114 ± 6	6.94	6
**16b**	CH	*(R)*	Pentyl	18%	-	273 ± 4	6.56	>100
**16c**	CH	*(S)*	5-F-Pentyl	716 ± 16	6.15	84.1 ± 1.9	7.08	8
**16d**	CH	*(R)*	5-F-Pentyl	24%	-	251 ± 7	6.60	>100
**16e**	CH	*(S)*	CH_2_–Cy	122 ± 8	6.91	45.1 ± 1.7	7.35	3
**16f**	CH	*(R)*	CH_2_–Cy	37%	-	93.1 ± 3.3	7.03	>100
**16g**	CH	*(S)*	4-F-Bn	90.3 ± 2.3	7.04	22.5 ± 1.1	7.65	4
**16h**	CH	*(R)*	4-F-Bn	35%	-	109 ± 4	6.96	>100
**16i (ISAM-CG538)**	N	*(S)*	Pentyl	685 ± 21	6.16	10.9 ± 0.5	7.96	62
**16j (ISAM-CG528)**	N	*(R)*	Pentyl	3%	-	89.1 ± 3.1	7.05	>100
**16k**	N	*(S)*	5-F-Pentyl	392 ± 5	6.41	10.6 ± 0.7	7.97	37
**16l**	N	*(R)*	5-F-Pentyl	5%	-	127 ± 5	6.90	>100
**16m**	N	*(S)*	CH_2_–Cy	162 ± 3	6.79	5.20 ± 0.4	8.28	31
**16n**	N	*(R)*	CH_2_–Cy	3%	-	164 ± 5	6.79	>100
**16o**	N	*(S)*	4-F-Bn	65.4 ± 2.4	7.18	4.10 ± 0.12	8.39	16
**16p**	N	*(R)*	4-F-Bn	5%	-	48.4 ± 1.8	7.32	>100
**Surinabant**	-		-	3.00 ± 0.2		400 ± 6		0.0075
**GW405833**	-		-	4772 ± 110		5.80 ± 0.4		822
**CP55**,**940**	-		-	1.01 ± 0.08		0.13 ± 0.08		7.76

a Displacement of specific [^3^H]­CP 55,940 binding in membrane preparations of human CB_1_R transfected in CHO–CB1 C3 cell line expressed as *K*_i_ in nM (*n* = 3) or percentage displacement of specific binding at 1 μM (*n* = 2).

b Displacement of specific [^3^H]­CP 55,940 binding in membrane preparations of human CB_2_R transfected in HEK-293T cells expressed as *K*_i_ in nM (*n* = 3) or percentage displacement of specific binding at 1 μM (*n* = 2).

The compounds are grouped into four series (**I**–**IV**, Figure 2). Tables 1 and 2 show the data for ester derivatives (Series **I and II**), while Tables 3 and 4 report the data for amides (Series **III** and **IV**). To help in identifying the most promising ligands and rapidly analyzing structure-selectivity relationships across the series, the selectivity index (SI), calculated as the affinity ratio (*K*_*i*_ CB_1_R/*K*_*i*_ CB_2_R), is also reported alongside the tables.

**Table 2:**
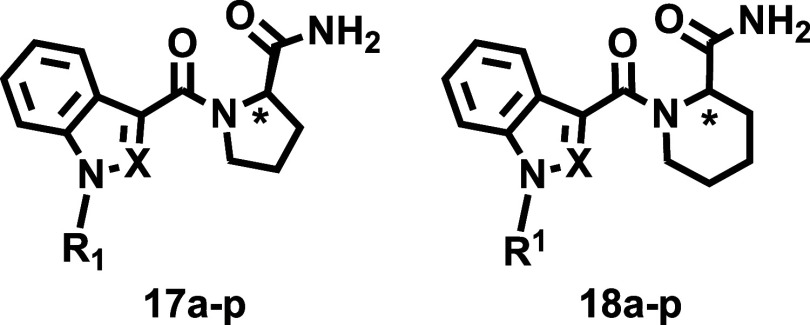
Structure and Binding Data Obtained for the Amides **17a**–**p** and **18a**–**p**

				CB_1_R	CB_2_R		
compound	X	*(*R*/*S*)	R^1^	*K*_*i*_ (nM) or % at 1 μM^a^	*K*_*i*_ (nM) or % at 1 μM^b^	p*K*_*i*_	SI *K*_*i*_ CB_1_R/ *K*_*i*_ CB_2_R
**17a**	CH	*(S)*	Pentyl	2%	24%	-	-
**17b**	CH	*(R)*	Pentyl	3%	17%	-	-
**17c**	CH	*(S)*	5-F-Pentyl	1%	15%	-	-
**17d**	CH	*(R)*	5-F-Pentyl	5%	16%	-	-
**17e**	CH	*(S)*	CH_2_–Cy	6%	288 ± 7	6.54	>100
**17f**	CH	*(R)*	CH_2_–Cy	3%	411 ± 16	6.39	>100
**17g**	CH	*(S)*	4-F-Bn	3%	20%	-	-
**17h**	CH	*(R)*	4-F-Bn	4%	7%	-	-
**17i**	N	*(S)*	Pentyl	2%	31%	-	-
**17j**	N	*(R)*	Pentyl	3%	28%	-	-
**17k**	N	*(S)*	5-F-Pentyl	2%	17%	-	-
**17l**	N	*(R)*	5-F-Pentyl	4%	25%	-	-
**17m**	N	*(S)*	CH_2_–Cy	1%	27%	-	-
**17n**	N	*(R)*	CH_2_–Cy	2%	28%	-	-
**17o**	N	*(S)*	4-F-Bn	4%	628 ± 21	6.20	>100
**17p**	N	*(R)*	4-F-Bn	4%	12%	-	-
**18a**	CH	*(S)*	Pentyl	3%	111 ± 6	6.95	>100
**18b**	CH	*(R)*	Pentyl	10%	136 ± 5	6.87	>100
**18c**	CH	*(S)*	5-F-Pentyl	7%	132 ± 4	6.88	>100
**18d**	CH	*(R)*	5-F-Pentyl	15%	169 ± 6	6.77	>100
**18e (ISAM-CG585)**	CH	*(S)*	CH_2_–Cy	22%	30.2 ± 1.2	7.52	>100
**18f (ISAM-CG586)**	CH	*(R)*	CH_2_–Cy	11%	96.8 ± 2.6	7.01	>100
**18g**	CH	*(S)*	4-F-Bn	21%	115 ± 5	6.94	>100
**18h**	CH	*(R)*	4-F-Bn	3%	207 ± 9	6.68	>100
**18i**	N	*(S)*	Pentyl	2%	91.8 ± 4.9	7.04	>100
**18j**	N	*(R)*	Pentyl	2%	145 ± 6	6.84	>100
**18k**	N	*(S)*	5-F-Pentyl	3%	151 ± 6	6.82	>100
**18l**	N	*(R)*	5-F-Pentyl	3%	143 ± 5	6.84	>100
**18m (ISAM-CG558)**	N	*(S)*	CH_2_–Cy	21%	59.5 ± 2.8	7.23	>100
**18n**	N	*(R)*	CH_2_–Cy	26%	112 ± 4	6.95	>100
**18o (ISAM-CG557)**	N	*(S)*	4-F-Bn	3%	54.6 ± 1.1	7.26	>100
**18p (ISAM-CG549)**	N	*(R)*	4-F-Bn	2%	120 ± 5	6.92	>100
**Surinabant**	-		-	3.00 ± 0.2	400 ± 6		0.0075
**GW405833**	-		-	4772 ± 110	5.80 ± 0.4		822
**CP55**,**940**	-		-	1.01 ± 0.08	0.13 ± 0.08		7.76

a Displacement of specific [^3^H]­CP 55,940 binding in membrane preparations of human CB_1_R transfected in CHO–CB1 C3 cell line expressed as *K*_i_ in nM (*n* = 3) or percentage displacement of specific binding at 1 μM (*n* = 2).

b Displacement of specific [^3^H]­CP 55,940 binding in membrane preparations of human CB_2_R transfected in HEK-293T cells expressed as *K*_i_ in nM (*n* = 3) or percentage displacement of specific binding at 1 μM (*n* = 2).

**Table 3:**
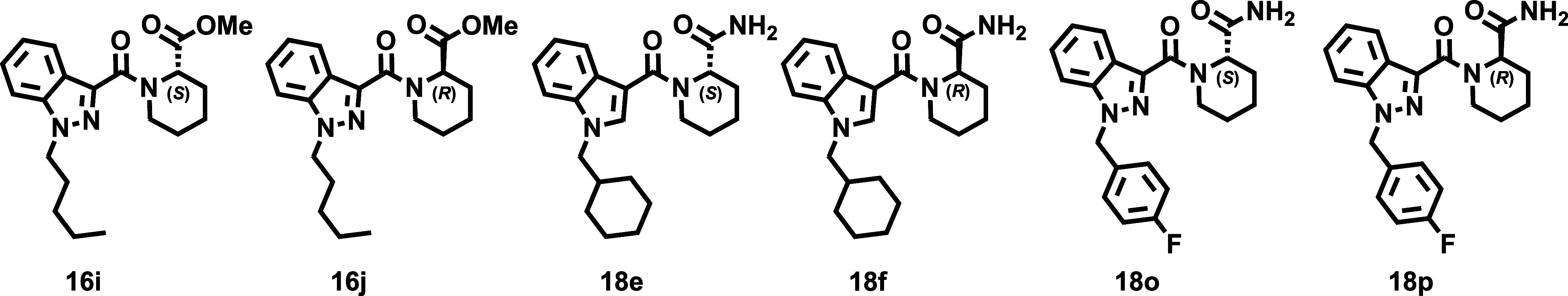
Structure, Affinity, and Functional Data of Selected Derivatives

		CB_2_R				Bias factor β-arrestin – cAMP^e^		Bias factor MAPK – cAMP^e^		Bias factor β-arrestin – MAPK^e^	
	CB_1_R		EC_50_ nM^c^ (*E*_max_)^d^								
Cpds	*K*_*i*_ or % at 1 μM^a^	*K*_*i*_ (nM)^b^	AMPc	β-arrestin	MAPK	$\Delta \Delta \log\left(\frac{E_{\max}}{EC_{50}}\right)$	$10^{\left|\mathrm{\Delta \Delta log}\left(\frac{E_{\max}}{EC_{50}}\right)\right|}$	$\Delta \Delta \log\left(\frac{E_{\max}}{EC_{50}}\right)$	$10^{\left|\mathrm{\Delta \Delta log}\left(\frac{E_{\max}}{EC_{50}}\right)\right|}$	$\Delta \Delta \log\left(\frac{E_{\max}}{EC_{50}}\right)$	$10^{\left|\Delta \Delta \,\log\left(\frac{E_{\max}}{EC_{50}}\right)\right|}$
**16i**	685 nM	10.9	0.03 ± 0.01 (64%)	1.09 ± 0.01 (48%)	0.38 ± 0.09 (88%)	–2.36 (G protein)	231 (G protein)	–1.36 (G protein)	23 (G protein)	–1.00 (MAPK)	10 (MAPK)
**16j**	3%	89.1	0.09 ± 0.01 (53%)	0.28 ± 0.02 (65%)	0.09 ± 0.003 (89%)	–1.08 (G protein)	12 (G protein)	–0.17 (No bias)	1 (No bias)	–0.91 (MAPK)	8 (MAPK)
**18e**	22%	30.2	1701 ± 12 (82%)	0.40 ± 0.08 (120%)	0.09 ± 0.002 (74%)	3.12 (β-arrestin)	1306 (β-arrestin)	3.84 (MAPK)	6843 (MAPK)	–0.72 (MAPK)	5 (MAPK)
**18f**	11%	96.8	118 ± 4 (55%)	0.08 ± 0.01 (88%)	0.07 ± 0.007 (94%)	2.69 (β-arrestin)	495 (β-arrestin)	3.06 (MAPK)	1156 (MAPK)	–0.37 (MAPK)	2 (MAPK)
**18o**	3%	54.6	0.60 ± 0.09 (60%)	60.9 ± 2.5 (148%)	0.03 ± 0.003 (68%)	–2.29 (G protein)	196 (G protein)	0.96 (MAPK)	9 (MAPK)	–3.25 (MAPK)	1783 (MAPK)
**18p**	2%	120	6.80 ± 0.3 (58%)	326 ± 4 (77%)	0.40 ± 0.02 (79%)	–2.24 (G protein)	172 (G protein)	0.97 (MAPK)	9 (MAPK)	–3.20 (MAPK)	1599 (MAPK)
**JWH-133**	-	-	6.73 ± 0.5 (98%)	1.34 ± 0.05 (93%)	2.70 ± 0.19 (98%)		1		1		1

a Displacement of specific [3H]­CP 55,940 binding in membrane preparations of human CB1R transfected in CHO–CB_1_R C3 cell line expressed as *K*_*i*_ in nM (*n* = 3) or percentage displacement of specific binding at a concentration of 1 μM (*n* = 2).

b Displacement of specific [3H]­CP 55,940 binding in membrane preparations of human CB_2_R transfected in HEK-293T cells expressed as *K*_*i*_ in nM (*n* = 3) or percentage displacement of specific binding at a concentration of 1 μM (*n* = 2).

c Functional activity assessed in transfected HEK-293T cells by determining cAMP levels after forskolin stimulation.

d *E*_max_ values were normalized to a maximal efficacious concentration of CP 55,940.

e Bias factors were quantified by the relative activity model using JWH-133 as a reference ligand (see the Experimental Section). The preferred (biased) pathway is indicated in parentheses.

**Table 4:**
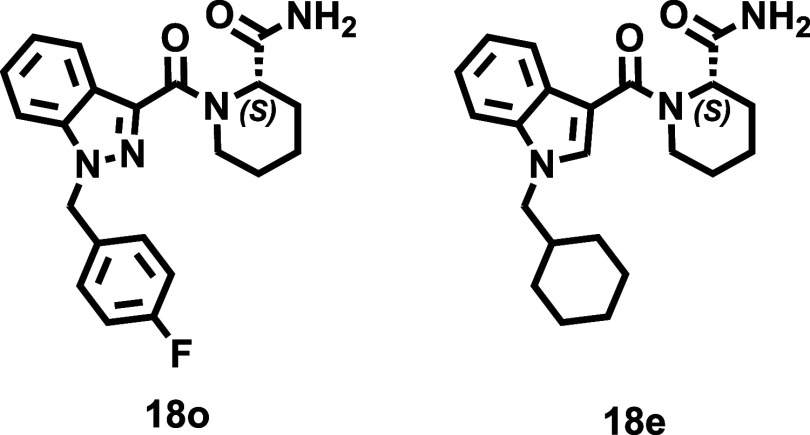
Solubility, Plasma Protein Binding and Metabolic Stability of Selected Ligands

		microsomal stability					
compound	solubility (μM)^a^	specie	%^b^	*t*_1/2_ ^c^	Cl_int_ ^d^	*h*CB_2_R *K*_*i*_ (nM)^e^	*h*CB_1_R % at 1 μM^f^
**18o**	43.7	Human	59.6	50.9	11.8	54.6	21%
		Mouse	28.4	28.0	26.1		
**18e**	40.1	Human	15.9	19.4	67.3	30.2	19%
		Mouse	22.3	21.8	54.3		
**Progesterone**	6.7	-	-	-	-	-	-
**Prazosin**	31.3	-	-	-	-	-	-
**Testosterone**	-	Human	7.11	16.13	32.44	-	-

a Solubility in 1:99 DMSO/PSB buffer.

b Percentage remanent (sampling time 60 min).

c *t*_1/2_ in min.

d Intrinsic clearance in μL·min^–1^·mg_prot_^–1^.

e Displacement of specific [^3^H]­CP 55,940 binding in membrane preparations of human CB_1_R transfected in CHO–CB_1_RC3 cell line expressed as *K*_i_ in nM (*n* = 3).

f Displacement of specific [^3^H]­CP55,940 binding in membrane preparations of human CB_2_R transfected in HEK-293T cells expressed as percentage displacement of specific binding at a concentration of 1 μM (*n* = 2).

### Structure–Activity and Structure-Selectivity Relationship Analyses

In this section, we review the binding data (Tables 1, 2) and discuss key aspects of the structure-affinity (SAR) and structure-selectivity (SSR) relationships within the series. These findings are further supported by molecular modeling studies using a refined 3D model of CB_2_R. As previously indicated, all ligands were tested as enantiopure compounds. For clarity, the collection was organized into four series, each containing 16 derivatives and exploring five points of diversity: the tail group (R^1^), the central scaffold (indole or indazole), the conformationally restricted amino acid linker (proline or homoproline), the functional group (ester or amide), and the stereochemistry [(*S*) and (*R*) enantiomers].

The cannabinomimetic affinity profiles of the conformationally constrained, analogues of abused SCRAs, obtained in this study are presented in the Tables 1, 2. As observed, nine ligands combine attractive CB_2_R affinity (*K*_*i*_ < 100 nM) and high subtype selectivity [e.g., compounds **15n**, **16f**, **16j**, **16p**, **18e**, **18f**, **18i**, **18m**, **18o**]. In addition to uncovering interesting structural diversity and SAR trends, the obtained data emphasize the benefits of pharmacophore rigidification as an effective strategy to achieving selective CB_2_R ligands starting from highly promiscuous (CB_1_R & CB_2_R) abused SCRAs (Figure 1), probably by capturing bioactive conformation. Among the four subsets (Tables 1, 2), derivatives containing the piperidine core and amide functional group (Series **IV**, Table 2) collectively emerge as the most appealing CB_2_R agonists. All derivatives in this subset exhibit high affinity and excellent subtype selectivity (>100-fold), regardless of the tail group at R^1^, the central bicyclic heterocycle, or the configuration at the piperidine nuclei. Compounds **18e** (**ISAM-CG585**), **18m** (**ISAM-CG558**), and **18o** (**ISAM-CG557**) stand out, exhibiting *K*_*i*_ values of 30.2, 59.5, and 54.6 nM, respectively, with almost negligible binding (∼20%) at CB_1_R (at a concentration of 1 μM).

To analyze the structure–activity and structure-selectivity relationships, the binding data from Tables 1, 2 were examined. For clearer visualization of both affinity and selectivity variations, the binding data (p*K*_*i*_ values) were graphically represented by plotting p*K*_*i*_ CB_1_R (Y axis) against p*K*_*i*_ CB_2_R (X axis), using an identical scale for both axes (Figures 6 and 7). Compounds with equal binding affinities to CB_1_R and CB_2_R will align along the diagonal (Y = X), while those with selective affinity toward CB_1_R or CB_2_R will appear below or above the diagonal, respectively. The distance of each compound from this diagonal serves as a direct indicator of its degree of selectivity.

A rapid understanding of the significant improvement in selectivity between the novel conformationally restricted derivatives (Compounds **15**–**18**) and the reference library of structurally analogous abused SCRAs (Figure 1) can be obtained by analyzing the data in Figure 4. As observed in the graphs, the majority of the classical abused SCRAs cluster near the diagonal (Figure 4 left), indicating their nonselective agonist profile. In contrast, many of the newly synthesized cyclic compounds (Figure 4 right) are positioned in the lower right region of the plot, demonstrating a clear shift toward greater selectivity for CB_2_R, thereby minimizing CB_1_R-related side effects while improving their therapeutic potential.

**Figure 4:**
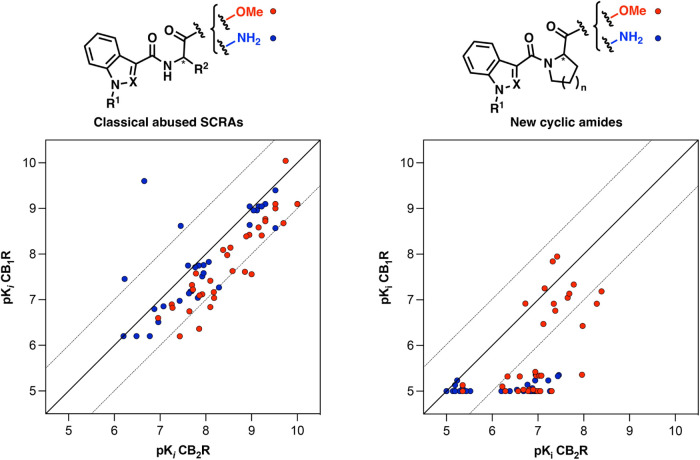
Affinity-selectivity plot for 64 classical abused SCRAs (left) and the 64 new derivatives (right) herein documented.

Although the esters reported here (Series **I** and **II**) were originally conceived as synthetic intermediates for the preparation of the targeted amides (Scheme 1), they were ultimately included in the pharmacological evaluation. While esters are generally not considered optimal drug candidates, their evaluation in this context provides valuable data for comparing the effects of rigidification on binding profiles in two functionally distinct series (esters and amides). This comparison is further supported by available binding data on structurally analogous abused ester SCRAs (Figure 1), offering deeper insights into structure–activity relationships. Additionally, the affinity data for esters contributed to identify key SAR trends, both within this group and in relation to their amide counterparts.

The analysis of the binding data for the ester series (Table 1) shows remarkably similar structure activity/selectivity relationship (SAR) patterns. Both subsets—prolinates [Table 1 and Figure 5 (left)] and homoprolinates [Table 1 and Figure 5 (right)]—exhibit promiscuous binding profiles for the (*S*) stereoisomers (Figure 5A squares), consistent with the nonselective behavior characteristic of both enantiomers in abused synthetic cannabinoids (SCRAs). The nonselective binding profile observed for (*S*) stereoisomers is largely independent of the R^1^ group, the central core (whether indole or indazole), or the size of the amino acid residue (proline or homoproline). Despite this promiscuity, subtle differences in binding affinity can be discerned. For prolinate derivatives (Table 1), compounds in the indole series generally show higher affinity for CB_1_R than for CB_2_R, whereas the opposite trend is noted in the indazole series. Additionally, ligands featuring cyclohexylmethyl or 4-fluorobenzyl groups at R^1^ tend to display superior binding affinities across both receptor types. A comparative analysis of the cannabimimetic binding profiles of the (*R*) stereoisomers from the ester series reveals marked differences compared to their (*S*) counterparts. The (*R*) stereoisomers generally exhibit high selectivity for CB_2_R across both ester series (Table 1). This selectivity is primarily achieved through a reduction in overall affinity, with the (*R*) derivatives displaying a 3- to 12-fold lower CB_2_R affinity (*K*_*i*_ = 99.3–599 nM) compared to the (*S*) stereoisomers. The most potent ligands in the series often feature cyclohexylmethyl or 4-fluorobenzyl groups at R^1^, with indazole derivatives generally displaying superior CB_2_R affinity. Notably, the piperidine derivative **16p** stands out, showing high affinity for CB_2_R (*K*_*i*_ = 48.4 nM) and negligible binding to CB_1_R at 1 μM.

**Figure 5:**
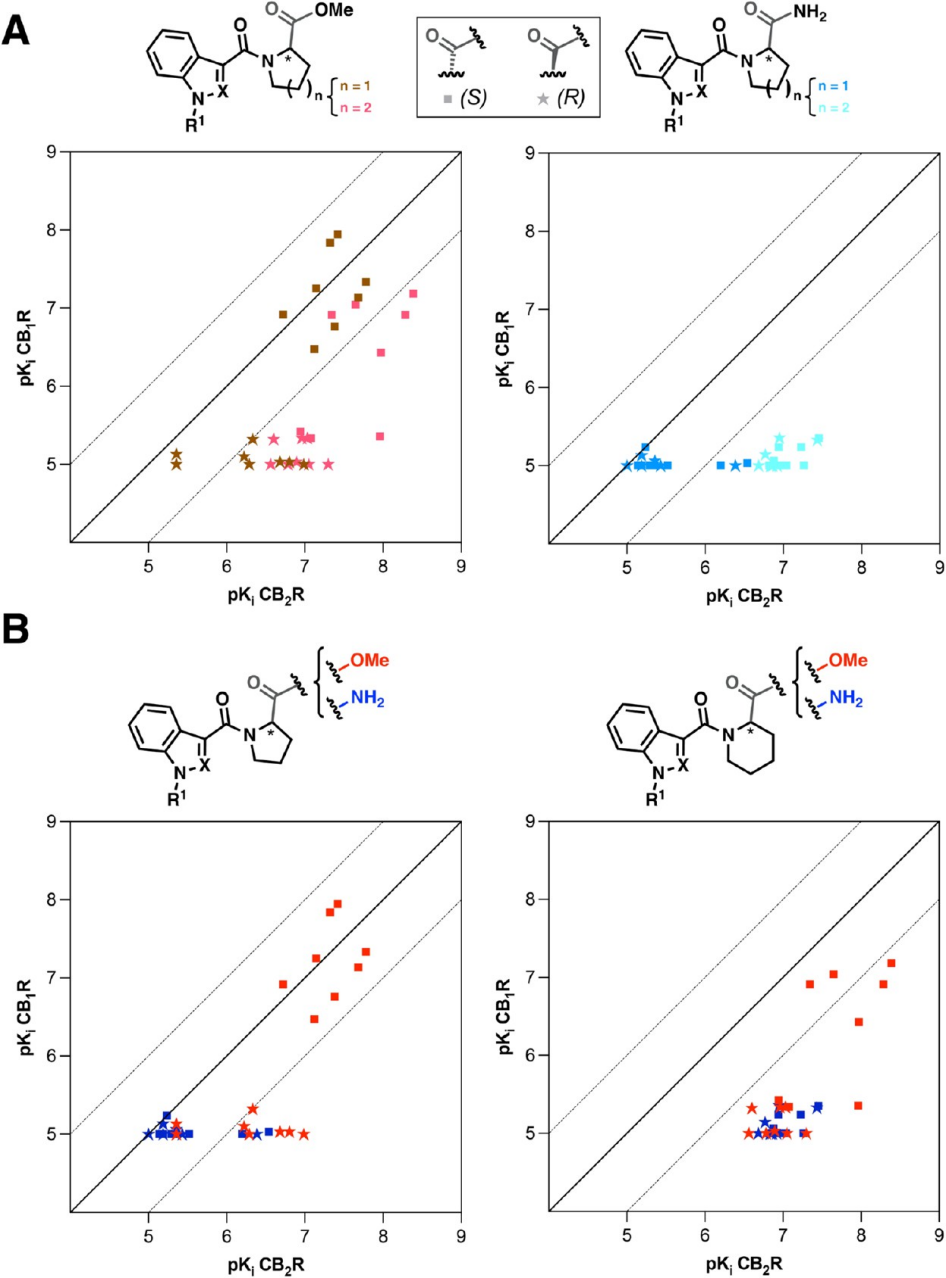
Affinity-selectivity plot for the 64 new derivatives herein documented clustered according to (A) their functional group or (B) ring size.

In sharp contrast to the consistent binding profiles and SAR trends seen in the ester series (**I** and **II**, Table 1), the affinity data obtained for the amide subsets (**III** and **IV**, Table 2) reveal markedly different binding characteristics. The conversion of esters in series **I** to its amide analogues (Series **III**) triggers a substantial shift in cannabimimetic binding effect (Figure 5). Notably, amides with a five-membered central amino acid residue (Table 2) largely lose their ability to bind either CB_2_R or CB_1_R. The few exceptions to this trend include indole derivatives **17e** and **17f**, and indazole-based amide **17o**, which display *K*_*i*_ values of 288, 411, and 628 nM, respectively, with no significant CB_1_R binding. It is also worth noting that enantiomers **17e** and **17f** both demonstrate moderate CB_2_R affinity, although the (*S*) stereoisomer shows slightly higher CB_2_R affinity.

The homoproline amides (series **IV**, Table 2) represent the most promising CB_2_R ligands identified in this study. In stark contrast to the previously discussed derivatives, particularly their ester counterparts (series **II**) and their inferior homologues pyrrolidine-based amides (series **III**), piperidine-based amides (series **IV**) display a significantly different cannabimimetic binding profile, both in terms of affinity and selectivity. As shown in Table 2, all members of this subset demonstrate moderate to high CB_2_R affinity while exhibiting negligible binding to CB_1_R. Notably, the expansion of the central cyclic nitrogenated core plays a crucial role, transforming inactive pyrrolidine derivatives (series **III**) into potent, highly selective CB_2_R ligands. A direct comparison between amides **IV** and their ester analogues (series **II**) further underscores the importance of the amide functional group in enhancing potency and selectivity. As observed (Figure 5 right bottom), transformation of the ester into an amide group produces an even more a significant loss of affinity for the CB_1_R receptor, particularly in the (*S*) enantiomers. The potency and selectivity profiles observed are consistent across the series, showing minimal influence from stereochemistry or the presence of a second nitrogen atom in the core structure (indole vs indazole). Changes in R^1^ similarly have a slight impact, except for the cyclohexylmethyl group, which generates the ligands with the highest affinity. A comparison of the CB_2_R affinity between (*R*) and (*S*) enantiomers throughout series **IV** indicates that they are generally equipotent, though (*S*) enantiomers occasionally demonstrate slightly higher affinity. The most notable differences (2–3-fold) in enantiomeric binding is observed for ligands bearing cyclohexylmethyl or 4-fluorobenzyl groups at R^1^, being consistently the (*S*) stereoisomers more potent than its (*R*) counterparts (e.g., compare **18e** and **18f**, **18m** and **18n**, **18g** and **18h**, **18o** and **18p**).

Since amide series **IV** (Table 2) led to the identification of the most potent (*K*_*i*_ < 100 nM) and selective CB_2_R ligands identified in this study (e.g., **18e**, **18m**, **18o**), these compounds, along with other structurally and functionally diverse analogs, were selected for in-depth analysis through computational tools and advanced pharmacological studies. The exploration encompassed their functional behavior, preferred signaling pathways, preliminary pharmacokinetic profiles, as well as their anti-inflammatory and neuroprotective effects.

### Molecular Docking

To provide a molecular basis for the obtained affinity data, we conducted docking simulations within the orthosteric binding site of the CB_2_R. As the starting protein structure, we employed the recently published X-ray data of the receptor complexed with an agonist (PDB code: 6KPC, released in 2020 ^46^). Notice that given the structural differences of the studied molecules with respect to the cocrystallized ligand (i.e., AM12033), flexibility was allowed for all protein cavity atoms during postdocking Molecular Mechanics energies combined with the Generalized Born and Surface Area continuum solvation (MM-GBSA) calculations.^47^ For the sake of clarity, we present below the molecular insights derived from the docking simulations for each SAR clue obtained from the experimental data. Figure 6 shows the complexes obtained after performing postdocking MM-GBSA calculations on the top-scored pose of ligands **11** (Figure 6A) and **12** (Figure 6B). Notably, compound **11** is predicted to establish a T-shaped π-π stacking interaction with F183. Additionally, its amide group interacts with F106 (aromatic H-bond) and H95 (H-bond interaction). Interestingly, methylation of this amide group disrupts these interactions, providing a possible explanation for the observed reduced CB_2_R affinity as indicated by the measured *K*_*i*_ (125 nM vs 540 nM returned by **11** and **12**, respectively). However, this substitution does not significantly alter the predicted binding mode, as the newly introduced methyl group engages in hydrophobic interactions with F91. The picture emerging from the analysis of the binding poses is consistent with the MM-GBSA free binding energy values, which are −63.79 kcal/mol and −56.19 kcal/mol for compounds **11** and **12**, respectively. In summary, the study provides a robust binding mode hypothesis for these compounds and offers a rational explanation of how methylation, although detrimental, does not result in a dramatic loss of CB_2_R affinity.

**Figure 6:**
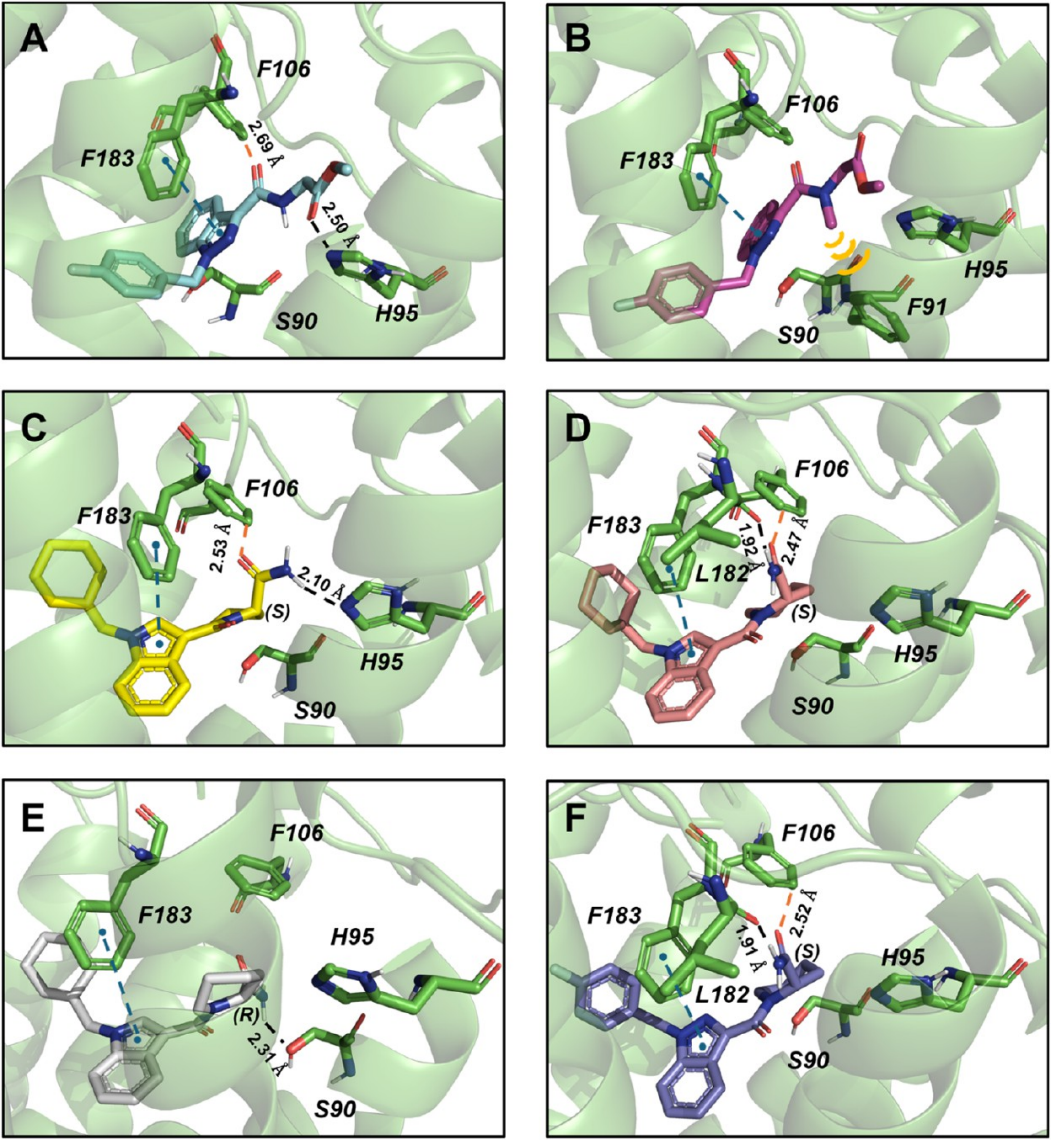
Protein–ligand complexes returned by MMGBSA calculations performed on the top-scored docking poses of: (A) **11**, (B) **12**, (C) **17e**, (D) **18e**, (E) **18f**, and (F) **18o**. π–π stacking interactions are shown as blue dashed lines, hydrogen bonds as black dashed lines, aromatic hydrogen bonds as red dashed lines, and hydrophobic interactions are indicated by yellow lines.

The effect of conformational restriction, as well as ring contraction, was studied using compounds **17e** (pentagonal amide, *K*_*i*_ = 288 nM) and **18e** (hexagonal amide, *K*_*i*_ = 30.2 nM) as model ligands [both (*S*) stereoisomers]. The top-scored poses obtained after MM-GBSA calculations are shown in Figure 6C (**17e**) and Figure 6D (**18e**). Remarkably, compound **17e** is predicted to interact with the receptor pocket through a binding mode very similar to that observed for compound **11** (Figure 6A). Specifically, it establishes a T-shaped π-π interaction with F183, an aromatic H-bond with F106, and a hydrogen bond with H95. The substitution of a pyrrolidine (series **III**) with a piperidine (series **IV**) results in a significant change in binding mode. The amide group, instead of interacting with H95, establishes a strong interaction with the backbone of L182. Consistent with the experimental data, this different binding mode translates into a distinct MMGBSA energy, estimated to be −64.03 kcal/mol (**17e**) and −71.65 kcal/mol (**18e**). Notably, the importance of the interaction with L182 is highlighted by a more detailed analysis of the individual contributions to the free binding energy. The difference, indeed, is primarily attributed to the electrostatic contribution (i.e., MM-GBSA dG Bind Coulomb), which increases from −1.77 kcal/mol (17e) to −12.48 kcal/mol (**18e**).

Since some of the enantiomeric pairs of the most intriguing series (series **IV**—piperidine derivatives) exhibits noticeable enantioselectivity, we aimed to investigate its underlying reasons by analyzing two enantiomers, namely **18e** (*K*_*i*_ = 30.2 nM) and **18f** (*K*_*i*_ = 96.8 nM). The complexes obtained after MM-GBSA calculations are shown in Figure 6D (**18e**-(*S*) enantiomer) and Figure 6E (**18f**-(*R*) enantiomer). As evident from the figures, the change in chirality results in a different orientation of the amide moiety within the pocket. Specifically, this moiety, which establishes a strong interaction with L182 in the (*S*) enantiomer, reorients in the (*R*) enantiomer to form an interaction with the side chain of S90. This altered orientation seems to be the responsible for the differing free binding energy values, consistent with the experimental data: −71.65 kcal/mol for **18e** and −63.65 kcal/mol for **18f**. Notably, this conformational adaptation within the pocket leads to a reduced contribution to the free energy in terms of Coulombic interactions (−9.57 kcal/mol vs −12.48 kcal/mol). Given that compound **18o** is one of the most promising candidates, the same protocol was applied to it. The complex obtained from the MM-GBSA calculations is shown in Figure 6F. Consistent with the previously observed trends and experimental data, **18o** is predicted to interact with CB_2_R in the same manner as **18e**, with its terminal amide forming a strong interaction with the backbone of L182. The binding free energy values support this finding, with a predicted value of −83.50 kcal/mol. Finally, as observed across the binding data analysis (Tables 1, 2), the similar binding modes obtained for **18o** and **18e** support the noncritical role of the second nitrogen atom and the residue at R^1^ for CB_2_R binding.

It is worth noting that, to gain molecular insights into the effect of the conformational restriction on CB_1_R affinity, **13** (*K_i_* = 268 nM) and **18e** (19% displacement at 1 μM) were docked into the CB_1_R binding site. The docking results indicate that the applied conformational restriction prevents **18e** from efficiently binding CB_1_R due to a steric hindrance with F177 (Figure S3).

### Intrinsic Activity Assays and Signaling Bias Evaluation

To gain deeper insight into the functional effects of the identified CB_2_R ligands, a subset of six ligands (**16i**, **16j**, **18e**, **18f**, **18o**, and **18p**) was selected based on their affinity, selectivity, and structural diversity

Initially we tested their ability to modify CB_2_R-mediated cAMP accumulation, providing insights into their functional behavior as either agonists or antagonists (Table 3). Moreover, the signaling profiles of selected ligands were investigated through their impact on β-arrestin recruitment and Mitogen-Activated Protein Kinase (MAPK) phosphorylation pathway, enabling a more comprehensive evaluation of their intracellular signaling profiles (Table 3).

A detailed description of the protocols employed is reported in the experimental part. An initial observation from the data (Table 3) reveals the identification of some ligands with EC_50_ values in the low picomolar range across various signaling pathways, accompanied by *E*_max_ values indicative of either partial or full agonist profiles. The dose–response curves for each ligand, evaluated across the different signaling pathways, are presented in Figure 7. The cAMP assay data presented in Table 3 and Figure 7, expressed as the maximal effect achieved by JWH-133, provide unequivocal evidence that tested ligands behave as CB_2_R agonists. A dose-dependent reduction in cAMP levels was observed for all evaluated compounds. As observed (Table 3), except for **18e**, most compounds exhibited EC_50_ values in the low nanomolar (e.g., **18p**, **18f**) or picomolar (e.g., **16i**, **16j**, and **18o**) range. It should be highlighted that marked EC_50_ differences (>10-fold) were observed (Table 3) for two enantiomer pairs (**18e**/**18f** and **18o**/**18p**) of the evaluated subset. For ligands **16i**, **16j**, and **18o**, the observed EC_50_ values indicated significantly greater functional activity compared to the reference agonist JWH-133. As observed, in this pathway (cAMP), all compounds behave as partial or full agonists, with *E*_max_ values ranging from 53 to 82%.

**Figure 7:**
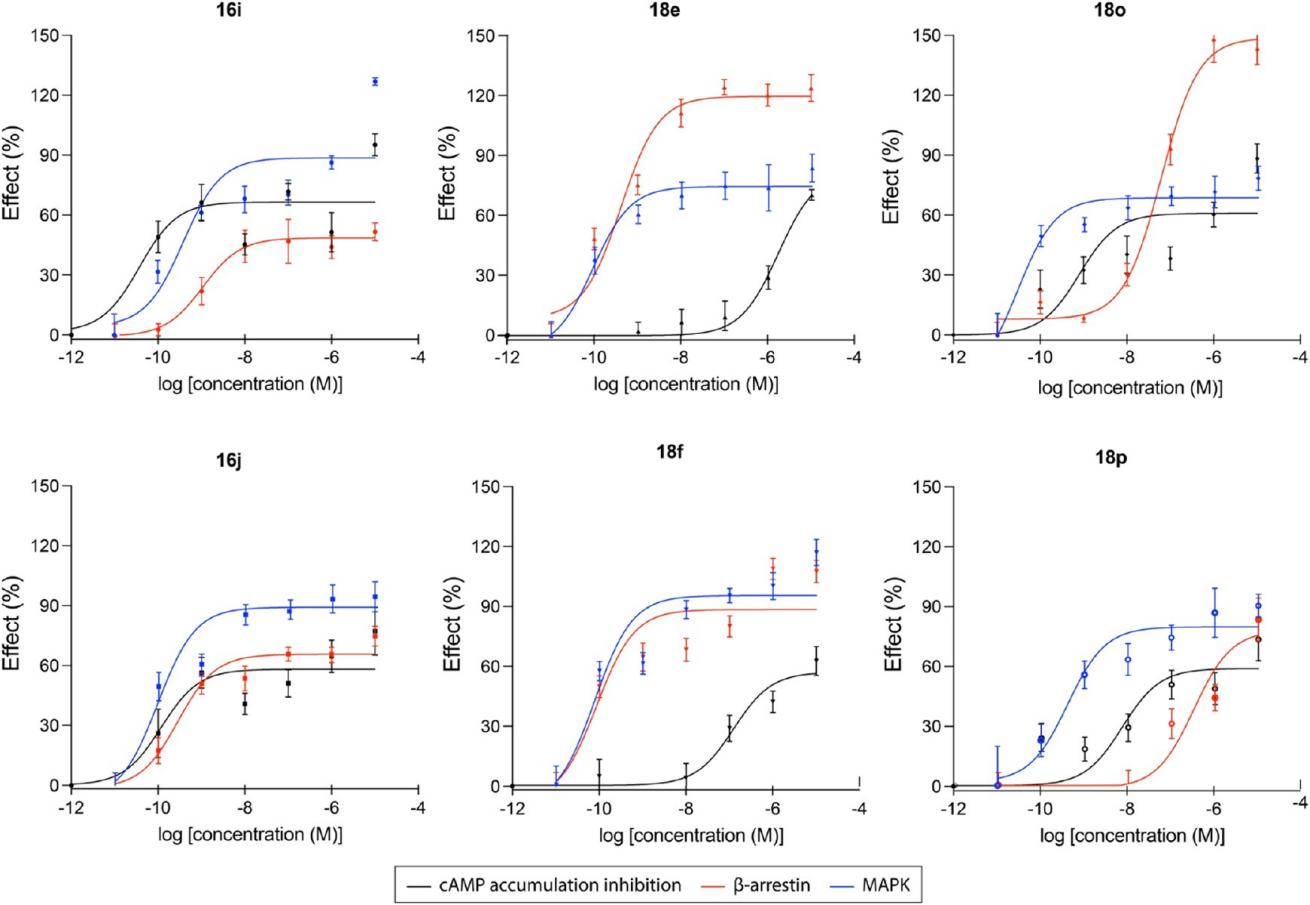
Dose–response curves (expressed as the percentage of the maximum decrease of forskolin-induced cAMP levels elicited by JWH-133) obtained for selected ligands across the different signaling pathways to assess its biased signaling profile at CB_2_R.

Biased agonists are ligands that exhibit preferential activation of certain signaling pathways over others, thereby offering the possibility of fine-tuning receptor-mediated responses. Accordingly, the study of signaling bias has gained great attention due to its potential to develop safer and more efficacious drugs.^48^ Although several CB_2_R-biased agonists have been identified,^49^ most of these agonists, to the best of our knowledge, exhibit limited subtype selectivity (e.g., CB_1_R vs CB_2_R).^49^ Because of this lack of subtype selectivity, it is difficult to precisely investigate and interpret biased signaling effects in complex cellular systems (i.e., where both receptors are coexpressed). Given the enormous therapeutic potential of CB_2_R agonists, particularly for treating challenging diseases and conditions such as neuroinflammation, pain, and immune disorders,^17,40^ it is essential to assess the signaling profiles of new subtype selective CB_2_R ligands to determine whether they exhibit significant bias toward intracellular pathways.^49^

To investigate the biased signaling of selected ligands (**16b**, **16j**, **18e**, **18f**, **18o**, and **18p**), we focused on three key pathways: cAMP modulation, β-arrestin recruitment, and MAPK phosphorylation, each associated with distinct downstream physiological outcomes.^10,50^ The compounds were assessed for their ability to engage these pathways by determining their EC_50_ values and efficacy (*E*_max_) in each assay (Figure 7). JWH-133, a well-known nonbiased (balanced) agonist,^49^ was employed as the reference for comparing the signaling profiles of the tested compounds. An operational model analysis^51^ was applied to quantify the functional bias of each CB_2_R-selective agonist. This involved calculating the bias factor by comparing the signaling pathways: β-arrestin versus cAMP, MAPK versus cAMP, and β-arrestin versus MAPK. By calculating the bias factors relative to JWH-133, nonbiased reference, it was possible to identify pathway-selective preferences of the compounds. The results, summarized in Table 3, highlight the distinct signaling preferences of each compound, allowing us to better understand their selectivity and potential for biased agonism.

Enantiomers **16i** and **16j** demonstrated a marked bias for G-protein pathway (Table 3), as evidenced by negative ΔΔlog­(*E*_max_/EC_50_) values in β-arrestin–cAMP and MAPK–cAMP comparisons. This suggests that these ligands preferentially activate G-protein signaling while minimizing β-arrestin and MAPK signaling. On the other hand, enantiomers **18e** and **18f** show a strong bias toward β-arrestin and MAPK pathways, with exceptionally high values in β-arrestin–cAMP and MAPK–cAMP comparisons, highlighting the predominant activation of the β-arrestin pathway among the evaluated compounds. Finally, compounds **18o** and **18p** display notable bias toward MAPK, especially in β-arrestin–MAPK signaling, indicating a selective MAPK pathway activation.

Understanding functional bias is crucial for designing drugs that selectively modulate CB_2_R signaling to meet specific therapeutic needs, while minimizing side effects linked to unwanted signaling. In this context, our newly identified compounds demonstrate both high subtype selectivity (most of them with negligible CB_1_R binding) and attractive biased profiles. This analysis demonstrates that each ligand’s specific bias profile could support tailored therapeutic applications, with **16i** and **16j** favoring G-protein pathways, while **18e**, **18f**, **18o**, and **18p** offer potential for therapies leveraging β-arrestin and MAPK pathway activation selectively. This differential signaling highlights their versatility and potential of these candidates for pathway-specific modulation in targeted therapeutic settings.

### Solubility and Microsomal Stability Exploration

As part of the profiling of selected ligands, and to complement the computational studies outlined in the previous sections (Figure 3), two CB_2_R agonists (**18o** and **18e**) were selected for preliminary pharmacokinetic characterization to assess their kinetic solubility and liver microsomal stability. These data provide preliminary insights into their *in vitro* ADMET profiles. As shown (Table 4), both ligands display moderate to satisfactory solubility (40–55 μM) in PBS at pH 7, indicating that structural rigidification did not significantly impact this property. These results are encouraging, as maintaining adequate solubility while introducing molecular rigidity is often challenging.

The microsomal stability study for compounds **18o** and **18e** reveals differences in metabolic stability between species and across compounds (Table 4). Compound **18o** stands out for its favorable stability in human microsomes, with 59.6% of the compound remaining after 60 min, a half-life of 50.9 min, and a low clearance rate of 11.8 μL·min^–1^·mgprot^–1^. These metrics suggest that **18o** is metabolically stable, making it a strong candidate for further development. However, in mouse microsomes, **18o** shows a faster metabolism, with only 28.4% remaining and a shorter half-life of 28 min, highlighting the species-specific metabolic differences often observed in preclinical models.^52^ Compound **18e**, on the other hand, exhibits poor stability in both human and mouse microsomes, with significantly lower remaining percentage (15.9% in humans and 22.3% in mice), shorter half-lives (19.4 min in humans and 21.8 min in mice), and much higher clearance rates (67.3 μL·min^–1^·mgprot^–1^ in humans), suggesting rapid metabolism that could limit its therapeutic potential.

It is important to highlight the structural differences between compounds **18o** and **18e**, which likely contribute to their distinct metabolic profiles. The key variations lie in the presence of a nitrogen atom at position 2 of the central scaffold (indole vs indazole) and the substituent at the R^1^ position (4-fluorobenzyl in **18o** vs cyclohexylmethyl in **18e**). Among these structural features, it is reasonable to hypothesize that the R^1^ group would have the most significant impact on their metabolic stability. The 4-fluorobenzyl group in **18o** may contribute to its superior microsomal stability, especially in human liver microsomes, by reducing susceptibility to metabolic degradation. In contrast, the cyclohexylmethyl group in **18e** could be more prone to metabolic transformations, accounting for the much higher clearance and shorter half-life observed across both human and mouse microsomes. This structural insight aligns with the superior pharmacokinetic profile of **18o** and underscores the importance of R^1^ group selection when designing metabolically stable drug candidates in these subsets.

### BBB Permeability and P-Glycoprotein Interaction Investigation

The therapeutic potential of CB_2_R agonists in treating neurodegenerative diseases, hinges critically on their ability to cross the blood-brain barrier (BBB) and evade active efflux mechanisms, such as that mediated by P-glycoprotein (P-gP). The BBB is a highly selective barrier that regulates the passage of substances into the central nervous system (CNS), making it essential to evaluate drug candidates’ permeability across this barrier.^53^ Furthermore, P-gP, a transporter protein expressed in the BBB, often actively pumps foreign substances out of the brain, limiting drug efficacy in CNS applications.^54^ Therefore, understanding both BBB permeability and P-gp interaction is crucial for developing CNS-acting agents, as effective compounds must exhibit both good brain penetration and minimal efflux to ensure sustained therapeutic concentrations within the CNS.^55^

To validate the early predictions from the CNS-MPO and BOILED-egg models (Figure 3), we experimentally assessed the BBB permeability and P-gp interaction of the selected CB_2_R agonists. For these studies, two pairs of amide enantiomers (**18p** and **18o**, **18e**, and **18f**) were selected, and the evaluations were conducted using established protocols previously published by our group.^20^ Key parameters measured included the permeability coefficients (*P*_app_BA and *P*_app_AB), the bidirectional permeability ratio (BA/AB), and the degree of interaction with P-gP, as detailed in Table 5.

**Table 5:**
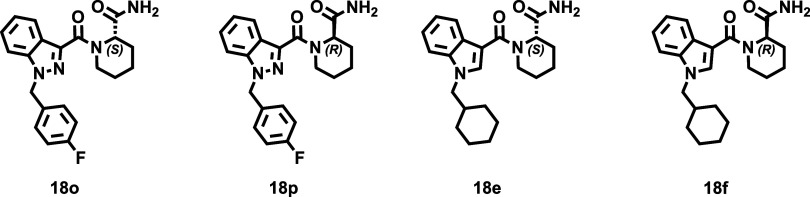
Evaluation of the BBB Permeability and P-gp Interaction of Selected CB_2_R

ligand	*P*_app_BA (nm/sec)	*P*_app_AB (nm/sec)	*P*_app_ (BA/AB)	P-gp interaction EC_50_ (μM) or % at 100 μM
**18o (ISAM-CG557)**	290	727	0.39	49%
**18p (ISAM-CG549)**	219	632	0.35	56.9 μM
**18e (ISAM-CG585)**	506	995	0.51	30%
**18f (ISAM-CG586)**	393	792	0.49	61.8 μM

The overall analysis of the experimental data (Table 5) reveals that the four tested compounds—**18p**, **18o**, **18e**, and **18f**—demonstrate good potential as CNS-targeted therapeutics based on their BBB permeability and P-glycoprotein (P-gp) interactions. All four compounds showed a noticeable ability to cross membranes, with particularly rapid transport from the apical compartment, where efflux pumps are expressed following polarization, to the basolateral compartment (*P*_app_AB). Because these derivatives exhibit low (EC_50_ ranging from 56.9 to 61.8 μM) or negligible (30 and 49% inhibition at 100 μM) interaction with P-gp, they are expected to readily cross the BBB and reach their CNS target, avoiding P-gp-mediated efflux. In the permeability assay, **18e** exhibited the most promising profile, with high *P*_app_BA (506 nm/sec) and *P*_app_AB (995 nm/sec) values and minimal P-gp interaction (30% inhibition at 1 μM), indicating strong BBB penetration and minimal efflux from the brain. This is further supported by its BA/AB ratio (0.51), indicative of balanced bidirectional permeability, thereby positioning it as a promising candidate for CNS applications, such as in neuroinflammation and neurodegenerative diseases. **18f** also performed well, exhibiting a slightly lower *P*_app_BA value of 393 nm/sec and a high *P*_app_AB value of 792 nm/sec, along with low P-gp interaction (61.8 μM). Compounds **18e**, **18p**, and **18o** all demonstrated predicted ability to cross the blood–brain barrier, with varying degrees of permeability and P-gp interaction. While **18e** showed higher passive influx, **18p** and **18o** also exhibited acceptable permeability profiles and moderate P-gp-mediated efflux. These results support the potential of all three compounds as viable candidates for CNS applications.

### Assessment of CB_1_R Activity to Evaluate Central Safety of CB_2_R-Selective Ligands

Given their excellent CB_2_R functional activity and ability to cross the blood–brain barrier, compounds **18p** and **18o** were selected for CB_1_R functional evaluation to assess potential risks of CB_1_R-mediated central effects. In a Gi protein–mediated cAMP inhibition assay, performed according to our previously described protocol,^29^ both compounds showed weak CB_1_R activation. Compound **18p** showed an EC_50_ of 755 ± 31 nM and *E*_max_ of 45%, while **18o** displayed even lower efficacy (36%) and potency (EC_50_ = 1071 ± 57 nM). In contrast, both ligands demonstrated markedly superior CB_2_R activity, with **18p** showing EC_50_ = 6.80 ± 0.3 nM and *E*_max_ = 58%, and **18o** achieving EC_50_ = 0.60 ± 0.09 nM and *E*_max_ = 60%, resulting in functional selectivity ratios of 111 (**18p**) and >1780 (**18o**). Full CB_1_R dose–response curves are provided in the Supporting Information (Figure S6). These results confirm that both compounds exhibit limited CB_1_R activity and are unlikely to trigger CB_1_R-mediated psychotropic effects at therapeutically relevant concentrations. Notably, compound **18o** stands out for its exceptional CB_2_R potency and functional selectivity, reinforcing its profile as a highly promising CB_2_R agonist with minimal risk of central side effects despite effective CNS penetration.

### Anti-Inflammatory Profiling of CB_2_R Agonists: Insights into Cytokine Production

The CB_2_R plays a crucial role in regulating immune and inflammatory responses.^17^ Unlike CB_1_R, which is predominantly expressed in the CNS system and associated with psychoactive effects, CB_2_R is primarily found in peripheral tissues, including immune cells (e.g., monocytes, macrophages, and microglia). Its activation has been linked to anti-inflammatory and immunomodulatory effects, making it a promising target in conditions where chronic inflammation plays a central role in disease progression, including cancer, neurodegenerative diseases, autoimmune diseases, and obesity.^15,40,56^ Some selective CB_2_R agonists have shown promise as therapeutic agents due to their ability to modulate cytokine profiles and reduce inflammation.^19,20^

Six representative CB_2_R agonists (**16i**, **16j**, **18e**, **18f**, **18o**, and **18p**) were evaluated for their ability to modulate the production of key pro- (e.g., TNF-α, IFN-γ, IL-1β, IL-6) and anti-inflammatory (e.g., IL-4, IL-10) cytokines in human monocytes and macrophages, where CB_2_R is abundantly expressed.^12^ The experiments were performed both in basal and LPS-activated states and would allow to gain insight into how these compounds affect circulating leukocytes (monocytes) and tissue-resident cells (macrophages). To mimic an inflammatory environment, both cell types were also treated with LPS, enabling an assessment of the anti-inflammatory potential of the compounds in both resting and activated states. Alongside the selected ligands, the study included HU308,^57^ a well-established CB_2_R-selective agonist known for its anti-inflammatory properties, and SR144528,^58^ a CB_2_R-selective antagonist. SR144528 was used alone and in combination with the test compounds to confirm that the observed effects were specifically mediated through CB_2_R activation.

As illustrated in Figure 8, all tested compounds (**16i**, **16j**, **18e**, **18f**, **18o**, and **18p**) significantly reduced the production of pro-inflammatory cytokines (TNF-α, IFN-γ, IL-1β, and IL-6) in unstimulated monocytes and macrophages, demonstrating comparable or even superior efficacy to the reference CB_2_R agonist HU308 (Figure 8, panel A and C, respectively). Consistently, selected ligands also enhanced the production of anti-inflammatory cytokines (IL-4 and IL-10) to levels like those observed with HU308 (Figure 8, panel A and C). The effects were even more pronounced in LPS-activated monocytes (Figure 8, panel B) and macrophages (Figure 8, panel D), where a greater modulation of both pro- and anti-inflammatory cytokine levels was detected. As expected, the CB_2_R antagonist SR144528 reversed these effects across most tested conditions, confirming CB_2_R-mediated activity.

**Figure 8:**
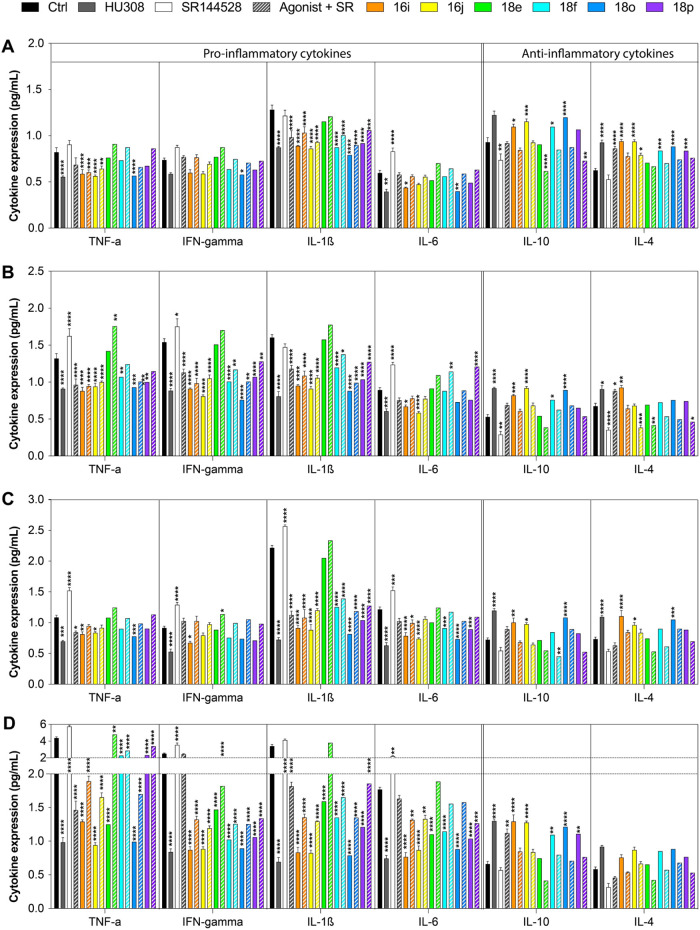
Cytokines levels in human monocytes (A, B) and macrophages (C, D) treated with CB_2_R ligands. THP-1 cells either untreated (A-B) or treated (C, D) with 0.01 μM PMA for 48 h to differentiate them into macrophages, were incubated for additional 24 h in the absence (A, C) or presence of 10 μg/mL LPS (B, D), without (Ctrl) or with the CB_2_R reference agonist HU308, the reference antagonist SR144528 and the CB_2_R ligands **16i**, **16j**, **18e**, **18f**, **18o**, **18p** at 100 nM. All the compounds were tested alone (solid bars) and in the presence of the CB_2_R antagonist SR144528 (striped bars). Cytokine levels were measured by ELISA coupled with qRT-PCR. Data are means ± SD (*n* = 3). Two-way ANOVA followed by Tukey’s posthoc test was applied. *p < 0.05; **p < 0.01; ***p < 0.001; ****p < 0.0001; * indicates vs Ctrl.

To further investigate the efficacy of our tested compounds, we conducted dose–response experiments on macrophages stimulated with LPS, comparing their effects to the known agonist HU308. The compounds were evaluated at concentrations of 1, 10, and 100 nM (Figure S4), showing a clear dose–response effect in all cases.

These findings collectively highlight the anti-inflammatory properties of the selected CB_2_R agonists. As observed (Figure 8, panel B and D), the maximum decrease in pro-inflammatory cytokines and the enhanced release of anti-inflammatory cytokines in LPS-stimulated cells indicate that these compounds are more effective in managing strong inflammatory responses, both in circulating immune cells (monocytes) and tissue-resident cells (macrophages). The cytokine modulation profiles observed (Figure 8) support the hypothesis that these ligands promote a shift toward an anti-inflammatory immune response, reducing the detrimental effects of chronic inflammation. This makes them promising candidates for the development of new therapies addressing severe inflammatory conditions, such as systemic infections, sepsis, neuroinflammation, and certain types of cancer or scenarios where conventional anti-inflammatory treatments are insufficient. Moreover, the selective targeting of CB_2_R without activating CB_1_R ensures a favorable safety profile, minimizing the risk of psychoactive side effects, and positioning these ligands as promising candidates for neurodegenerative and oncological therapies. To ensure the safety and viability of our tested compounds, we evaluated their cytotoxic effects on monocytes and macrophages under the same conditions as those used for assessing anti-inflammatory activity (Figure 8). Each compound was tested at a concentration of 100 nM. The cytotoxicity assays confirmed that the compounds did not adversely affect cell viability, supporting their potential as therapeutic agents (Figure S5).

Among the six evaluated CB_2_R ligands, distinct cytokine modulation profiles were observed, possibly reflecting their unique signaling bias. **16i** and **16j**, with a pronounced bias toward cAMP-mediated G-protein signaling, and **18o** and **18p**, strongly biased toward MAPK signaling, demonstrated a significant reduction in pro-inflammatory cytokines such as TNF-α, IFN-γ, and IL-1β, while also increasing anti-inflammatory cytokines like IL-10 and IL-4 (Figure 8). In contrast, **18e** and **18f**, which are predominantly biased toward β-arrestin, showed limited efficacy in reducing pro-inflammatory cytokines and promoting anti-inflammatory ones. The stronger recruitment of β-arrestin may underlie this suboptimal profile, as it appears less effective in shifting the immune balance toward an anti-inflammatory state. These findings suggest that excessive β-arrestin signaling might not be ideal for resolving inflammatory responses and could contribute to pro-inflammatory conditions under certain circumstances, as previously reported in studies exploring the impact of β-arrestin-biased ligands at CB_2_R.^59,60^

Given the subtype selectivity and biased signaling profiles of the herein developed CB_2_R agonists, we investigated how their ΔΔlog­(*E*_max_/EC_50_) values relative to β-arrestin correlate with cytokine modulation. Given the complex nature of biased signaling, we selected cAMP vs β-arrestin as a representative comparison to simplify the interpretation of the data. This approach allows us to clearly delineate the pro- and anti-inflammatory profiles of the biased ligands without introducing additional layers of complexity inherent to three-way comparisons. Additional analyses, including cAMP vs MAPK and β-arrestin vs MAPK, are provided in Figure S6 for further exploration of these signaling axes. As shown in Figure 9, the cytokine expression profiles for both pro-inflammatory (TNF-α, IFN-γ, IL-1β, IL-6) and anti-inflammatory (IL-10, IL-4) cytokines vary distinctly along the bias axis from cAMP to β-arrestin. Ligands with a stronger bias toward β-arrestin [positive ΔΔlog­(*E*_max_/EC_50_) values] are associated with an increase in pro-inflammatory cytokines (TNF-α, IFN-γ) and a reduction in anti-inflammatory cytokines (IL-10, IL-4). Conversely, ligands biased toward cAMP [negative ΔΔlog­(*E*_max_/EC_50_) values] show a decrease in pro-inflammatory cytokines and an increase in anti-inflammatory cytokines, highlighting the beneficial effects of cAMP signaling in suppressing inflammation and promoting resolution. This trend is consistent across both monocyte and macrophage populations, under basal and LPS-stimulated conditions.

**Figure 9:**
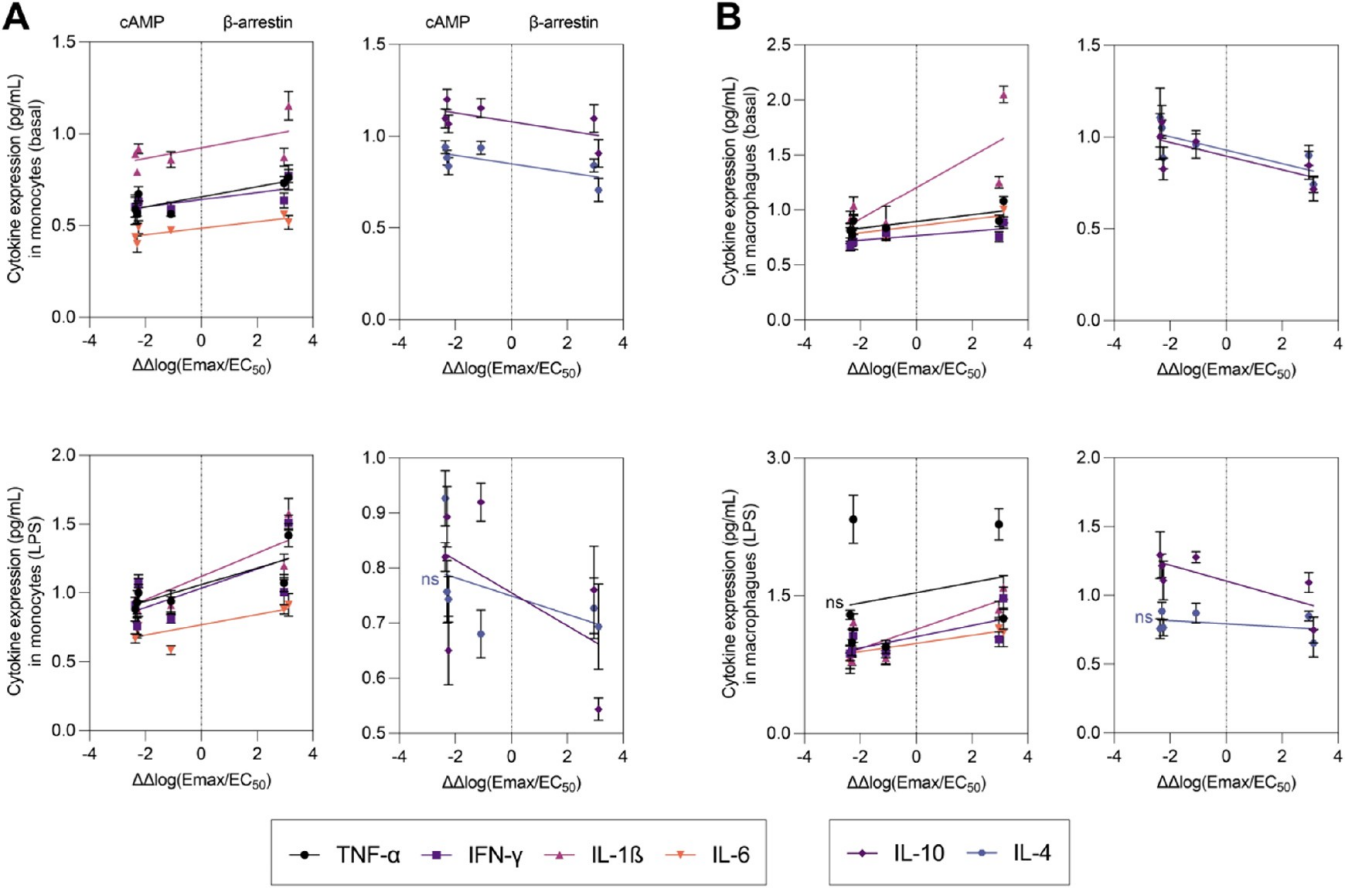
(A) Linear correlation between Δlog­(*E*_max_/EC_50_) for cAMP and β-arrestin pathways and cytokine expression (TNF-α, IFN-γ, IL-1β, IL-6, IL-10, IL-4) for **16i**, **16j**, **18e**, **18f**, **18o** and **18p** in (A) monocytes and (B) macrophages. Slopes are significantly different from 0 (*p* < 0.005) except those marked as ns. Data are presented as mean ± SEM.

These results suggest that β-arrestin-biased CB_2_R agonists exhibit a less favorable anti-inflammatory cytokine profile, thereby limiting their effectiveness in promoting an anti-inflammatory response. In contrast, CB_2_R agonists biased toward cAMP-mediated signaling demonstrate a more favorable profile in reducing inflammation. The key challenge seems to lie in mitigating β-arrestin dominance. This effect may be attributed to enhanced internalization and desensitization of CB_2_R, resulting in reduced signaling activity and diminished surface receptor levels.^61^ Thus, the development of cAMP-biased CB_2_R agonists, characterized by minimal β-arrestin recruitment, may represent a more effective strategy for achieving robust and sustained anti-inflammatory effects.

### Evaluation of the Neuroprotective Effect of Selected CB_2_R Agonists

Activation of the CB_2_R by selective small molecules offers significant neuroprotective potential, particularly for neurodegenerative diseases.^62^ Primarily expressed in immune cells and microglia in the CNS, CB_2_R activation reduces neuroinflammation by lowering pro-inflammatory cytokines and promoting an anti-inflammatory state.^63^ This effect is critical in pathologies such as Alzheimer’s, Parkinson’s, and Multiple Sclerosis, where inflammation plays a key role.^16,64^ Additionally, CB_2_R activation has been shown to decrease oxidative stress and apoptosis in neurons, supporting cell survival. Unlike CB_1_R, CB_2_R modulation avoids psychoactive effects, making it an attractive target for neuroprotective therapies.

A mouse primary cell experiment was performed to examine the neuroprotective effects of CB_2_R agonists selected for in-depth study (**16i**, **16j**, **18e**, **18f**, **18o**, and **18p**). First, to exclude any intrinsic toxicity of the selected candidates, their potential effects on primary cell cultures were assessed. The results (Figure 10A, colored bars) unequivocally confirmed that the selected CB_2_R agonists did not affect primary cell survival. This finding is particularly noteworthy, and not self-evident, given the significant neurotoxicity observed during our earlier study with structurally related, classical abused [CB_1_R/CB_2_R promiscuous] synthetic cannabinoids (SCRAs).^29^ For comparative purposes, Figure 10A also depicts cell viability data obtained for some abused SCRAs (Figure 10A, gray bars) under identical conditions. These data underscore that the conformational restriction strategy employed here successfully achieves a complete reversal of the neurotoxicity signatures associated with promiscuous (CB_1_R and CB_2_R) abused SCRAs, which exhibited reductions in cell viability of up to 70%.^29^ Notably, the strikingly different toxicological profiles observed between these two subsets (Figure 10A), despite their high structural similarity, strongly suggest that the neurotoxicity associated with abused SCRAs is predominantly driven by CB_1_R activation rather than being an intrinsic property of the molecular scaffold.

**Figure 10:**
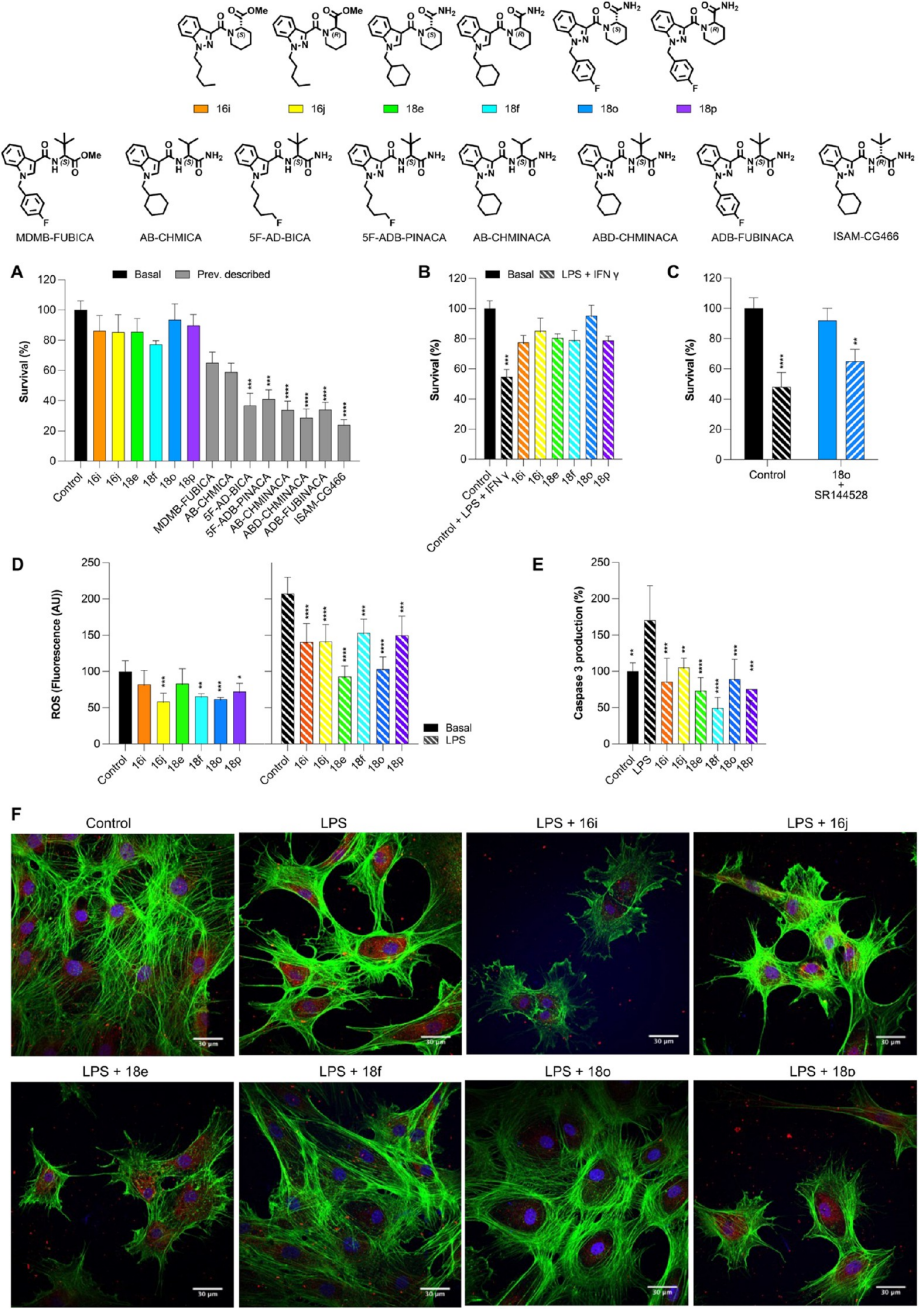
Neuroprotective effects of compounds **16i**, **16j**, **18e**, **18f**, **18o**, and **18p** in primary cultures in (A) basal conditions compared to previously reported SCRAs^30^ (gray bars) and (B) following LPS + IFN-γ stimulation. Asterisks (*) indicate statistical significance vs the respective control group. (C) The CB_2_ receptor antagonist SR144528 reversed the protective effect of **18o**. Data represent mean ± SEM (*n* = 9 per group). Statistical analysis was performed using Two-Way ANOVA followed by Sidak’s multiple comparisons test. Asterisks (*) indicate statistical significance vs basal conditions. (D) Reduction in ROS levels in both basal and LPS-stimulated conditions. Asterisks (*) indicate statistical significance vs LPS treatment. (E) Decrease in caspase activity under the same conditions. Asterisks (*) indicate statistical significance vs LPS treatment. (F) Representative immunofluorescence images showing cellular morphology changes. Data represent mean ± SEM (*n* = 9 per group). Statistical analysis was performed using Two-Way ANOVA followed by Sidak’s multiple comparisons test.

Following the examination of the neuroprotective effects of herein developed CB_2_R agonists (Figure 10), the selected derivatives (**16i**, **16j**, **18e**, **18f**, **18o**, and **18p**) were evaluated in primary cultures exposed to LPS + IFN-γ, which caused a significant reduction (approximately 50%) in cell viability due to the induced inflammatory conditions. As shown in Figure 10B, the addition of the studied compounds produced a marked recovery in cell viability (ranging from 77 to 93%), indicating their strong neuroprotective properties. Among the tested compounds, ligand **18o** emerged as the most promising neuroprotective candidate, demonstrating an exceptional ability to nearly fully restore cell viability. These findings demonstrate the potential of these novel CB_2_R agonists as promising therapeutic candidates for neuroinflammatory conditions. To unequivocally confirm that the observed neuroprotective effect was indeed mediated by CB_2_R activation, an additional experiment was conducted (Figure 10C). In this experiment, primary cultures treated with LPS + IFN-γ, which exhibited reduced cell viability, were treated with compound **18o** in one set and with **18o** alongside the selective CB_2_R antagonist SR144528 in another. As illustrated in Figure 10C, the addition of ligand **18o** almost fully restored cell viability to near 100%. However, the presence of the antagonist (SR144528) effectively blocked the neuroprotective action of **18o**, preserving the reduced viability (∼60%) observed with LPS + IFN-γ treatment alone. These results unequivocally demonstrate the significant neuroprotective effects of the CB_2_R agonists are mediated through CB_2_R activation. Taken together, these results exemplify the beneficial outcomes of applying conformational restriction design criteria, transforming promiscuous, neurotoxic ligands into highly selective and promising neuroprotective agents.

To further elucidate the mechanisms underlying these effects, we assessed markers of oxidative stress and apoptosis, two central pathways involved in neurodegeneration. Excessive production of reactive oxygen species (ROS) is a hallmark of neuroinflammatory and neurodegenerative conditions, contributing to cellular damage by promoting lipid peroxidation, protein oxidation, and mitochondrial dysfunction. Additionally, activation of executioner caspases such as caspase-3 plays a pivotal role in the progression of apoptosis, ultimately leading to neuronal loss. As shown in Figure 10D, treatment with the compounds resulted in a significant reduction in intracellular ROS levels under both basal and LPS-stimulated conditions, indicating a potent antioxidant effect. Furthermore, as illustrated in Figure 10E,F, a marked decrease in caspase activity was observed, consistent with an attenuation of apoptotic signaling. Together, these findings confirm the compounds’ capacity to mitigate oxidative damage and prevent programmed cell death, supporting their neuroprotective potential through dual modulation of redox balance and apoptosis.

Finally, to further validate these effects, a neuron-like *in vitro* model using differentiated SH-SY5Y cells was employed (Figure 11). This model reproduces key features of neuronal dysfunction, most notably neurite shortening, by recapitulating cellular stress pathways relevant to neurodegeneration. The SH-SY5Y cells were sequentially differentiated with retinoic acid and GLP-1 to induce a mature neuronal phenotype exhibiting dopaminergic and glutamatergic traits.^65^ To simulate neurodegenerative conditions, cells were transiently transfected with expression plasmids encoding the MAPT P301L and APP V717I mutations. The MAPT P301L mutation promotes pathological tau hyperphosphorylation and aggregation, leading to microtubule destabilization, impaired axonal transport, and neurite retraction.^66,67^ Meanwhile, the APP V717I mutation increases the production of long, aggregation-prone amyloidogenic peptides, disrupting cellular homeostasis through oxidative stress and cytoskeletal disorganization.^68,69^

**Figure 11:**
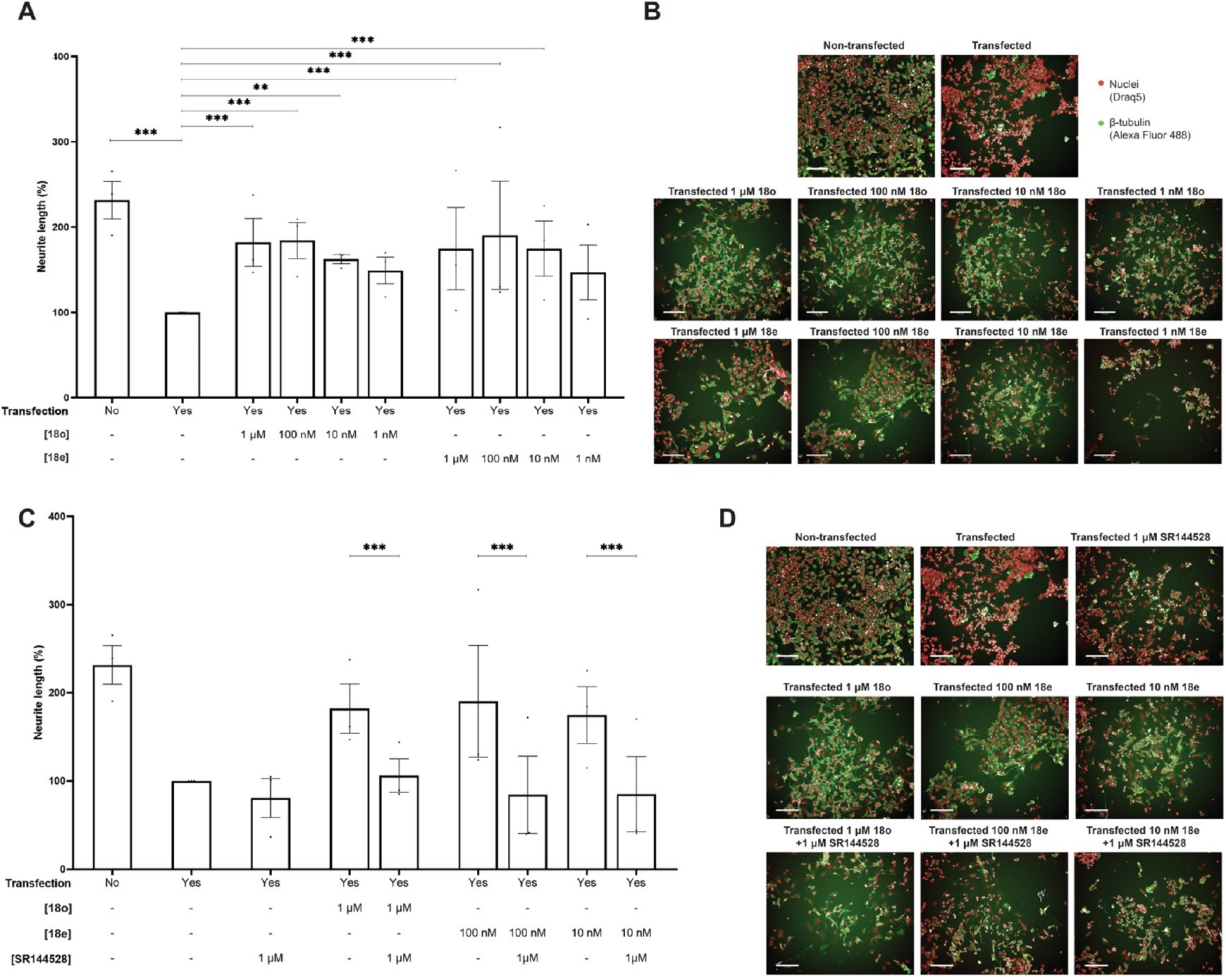
**18o** and **18e** elicit a concentration-dependent neuroprotective effect on neurite length in transfected SH-SY5Y cells. (A) Bar graph showing mean neurite length in nontransfected cells, transfected cells (*MAPT* P301L + *APP* V717I), and transfected cells treated with **18o** or **18e** at concentrations ranging from 1 nM to 1 μM. Each data point represents the mean of technical replicates from an independent biological experiment (*N* = 3). Bars represent mean ± SEM across experiments. ***p < 0.001, **p < 0.01 (two-way ANOVA followed by Sidak’s post hoc test). (B) Representative images of each condition acquired with a 20× objective. Scale bar = 100 μm. (C) Bar graph showing neurite length in SH-SY5Y cells transfected with *MAPT* P301L and *APP* V717I, treated with 18o (1 μM) or 18e (100  and 10 nM), in the presence or absence of the CB_2_R antagonist SR144528 (1 μM). Each data point represents the mean of technical replicates from an independent biological experiment (*n* = 3). Bars represent mean ± SEM across experiments. Bars represent mean ± SEM across experiments. *p* < 0.001 (two-way ANOVA followed by Sidak’s post hoc test). (D) Representative images of each condition acquired with a 20× objective. Scale bar = 100 μm.

The combined expression of these mutations in differentiated SH-SY5Y cells results in a reduction in neurite complexity, which is quantifiable through high-content imaging of neurite length and arborization, thereby establishing a phenotypic readout for compound screening. This model has been validated using well-known neuroprotective agents such as melatonin and resveratrol.^70^ In our study, a significant concentration-dependent protective effect against neurite shortening induced by plasmid transfection was observed for both **18o** and **18e** at concentrations ranging from 10 nM to 1 μM (Figure 10A,B). At 1 nM, neither compound produced a statistically significant effect. Notably, the neuroprotective effect of **18o** and **18e** at both 100 and 10 nM was reversed by the CB_2_R antagonist SR144528 (Figure 11C,D), reinforcing that the neuroprotection is mediated through CB_2_R activation.

## Conclusions

Herein, we documented the development of a new series of structurally simple, potent, subtype-selective and biased CB_2_R agonists with notable anti-inflammatory and neuroprotective effect. Building on the promiscuous (CB_1_R & CB_2_R) profiles of abused synthetic cannabinoid receptor agonists (SCRAs), a conformational restriction approach was applied to effectively eliminate CB_1_R binding. Affinity and functional data of the newly synthesized library highlighted key structure–activity relationships and identified specific signaling pathway biases, either favoring β-arrestin and MAPK or G-protein pathways, likely contributing to the compounds’ robust anti-inflammatory and neuroprotective effects. The neuroprotective profile was further supported by functional data showing that representative compounds significantly reduced intracellular ROS levels and caspase activation, mitigating oxidative stress and apoptosis in neuroinflammatory settings. In a physiologically relevant neuron-like model using differentiated SH-SY5Y cells expressing MAPT and APP mutations, the compounds effectively prevented neurite shortening—a hallmark of early neuronal dysfunction—in a CB_2_R-dependent manner. These cellular and phenotypic findings corroborate the compounds’ ability to preserve neuronal integrity in pathologically relevant contexts. Moreover, this series demonstrated favorable pharmacokinetic properties, including solubility, strong blood–brain barrier (BBB) permeability, low P-glycoprotein interaction, and microsomal stability, reinforcing their potential for CNS-targeted applications. Altogether, these results support the therapeutic promise of these CB2R agonists as safer, targeted treatments for neuroinflammatory and neurodegenerative diseases, devoid of CB_1_R-mediated psychoactive side effects.

## Experimental Section

### Chemistry

All starting materials, reagents and solvents were purchased and used without further purification. After extraction from aqueous phases, the organic solvents were dried over anhydrous magnesium sulfate. Unless stated otherwise, UV light and/or iodine vapor were used to detect compounds. The amide couplings and the ammonolysis reactions were performed in coated Kimble vials on a PLS (6 × 4) Organic Synthesizer with orbital stirring. The purity and identity of all tested compounds were established by a combination of HPLC, mass spectrometry and NMR spectroscopy. Purification of isolated products was carried out by column chromatography (Kieselgel 0.040–0.063 mm, E. Merck) or medium pressure liquid chromatography (MPLC) on a Combi Flash Companion (Teledyne ISCO) with RediSep prepacked normal-phase silica gel (35–60 μm) columns. The NMR spectra were recorded on Bruker AM300 and XM500 spectrometers. Chemical shifts are given as δ values against tetramethylsilane as internal standard and *J* values are given in Hz. Mass spectra were obtained on a Varian MAT-711 instrument. High-resolution mass spectra were obtained on an Autospec Micromass spectrometer.

The purity of all tested compounds was determined to be >95% using analytical HPLC [Water Breeze 2 system (binary pump 1525, detector UV/Visible 2489, 7725i Manual Injector Kit 1500 Series)] using a Luna 5 μm Silica (2) 100 Å, LC Column 150 × 4.6 mm column with gradient elution using the mobile phases dichloromethane, isopropanol, and a flow rate of 1 mL/min. The stereochemical purity of described compounds was analyzed and confirmed (e.e. >97%) by chiral HPLC. A detailed description of the experimental protocols, equipment and techniques used in the synthesis of targeted ligands, as well as the structural and spectroscopic data obtained for all the compounds described is given in the Supporting Information.

#### General Procedure for the Synthesis of Esters **15a**–**p** and **16a**–**p**

A mixture of the 1-substituted indole (or indazole)-3-carboxylic acid (1 mmol) (**19**), the corresponding cyclic amino ester (**20**) (1.5 mmol), HATU (1.5 mmol) and DIPEA (4 mmol) in CH_2_Cl_2_ (2 mL) was stirred with orbital stirring at room temperature for 24 h

After completion of the reaction, water was added, and the mixture was extracted with CH_2_Cl_2_. The organic phase was dried over MgSO_4_, filtered, and concentrated. The resulting product was purified by column chromatography on silica gel. The purity of all prepared compounds was established by high-performance liquid chromatography (HPLC) and showed to be >95%.

#### General Procedures for the Synthesis of Amides **17a–p** and **18a–sp**

A mixture of the corresponding ester (**15** or **16**), and a solution 4 M of ammonia (4 mmol) in MeOH (2 mL) was stirred at room temperature for 24 h. After completion of the reaction, solvent was evaporated. The resulting product was purified by column chromatography on silica gel using MeOH/CH_2_Cl_2_. The purity of all prepared compounds was established by high-performance liquid chromatography (HPLC) and showed to be >95%.

### Circular Dichroism (CD)

CD spectra of ligands (>98%) were recorded on a Jasco-815 system equipped with a Peltier-type thermostatic accessory (CDF-426S, Jasco). Measurements were carried out at 20 °C using a 1 mm quartz cell in a volume of 300–350 μL. Compounds (0.1 mg) were dissolved in MeOH (1.0 mL). The instrument settings were bandwidth, 1.0 nm; data pitch, 1.0 nm; speed, 500 nm/min; accumulation, 10; wavelengths, 400–190 nm.

### Binding Assays

Radioligand binding competition assays of CB_1_ receptors were carried out in polypropylene 96-well plates by incubating 5 μg of membranes from Chinese hamster ovary (CHO)-CB_1_ C3 cell line (PerkinElmer) with 1 nM [^3^H]-CP55940 (PerkinElmer) and test compounds in binding buffer (50 mM Tris-HCl, 5 mM MgCl_2_, 1 mM EDTA, 0.5% BSA. pH: 7.4). Nonspecific binding was determined in the presence of 10 μM Surinabant. The reaction mixture was incubated at 30 °C for 60 min, then 200 μL were transferred to GF/C 96-well plate (Millipore, Madrid, Spain) and washed four times with 250 μL wash buffer (50 mM Tris-HCl, 5 mM MgCl_2_, 1 mM EDTA, 0.5% BSA. pH: 7.4). Radioactivity was detected in a microplate β scintillation counter (Microbeta Trilux, PerkinElmer, Madrid, Spain). Radioligand binding competition assays of CB_2_ receptors were carried out in polypropylene 96-well plates by incubating 5 μg of membranes from Human embryonic kidney 293 cells (HEK)-CB_2_ cell line with 0.2 nM [^3^H]-CP55940 (PerkinElmer) and test compounds in binding buffer (50 mM Tris-HCl, 5 mM MgCl_2_, 2.5 mM EGTA, 0.1% BSA. pH: 7,4). Nonspecific binding was determined in the presence of 10 μM GW405833. The reaction mixture was incubated at 30 °C for 90 min, then 200 μL were transferred to GF/C 96-well plate (Millipore, Madrid, Spain) and washed four times with 250 μL wash buffer (50 mM Tris-HCl, 5 mM MgCl_2_, 2.5 mM EGTA, 1% BSA. pH: 7,4). Data was fitted to 4-parameter logistic equation by employing GraphPad Prism software (V7.0) and *K*_*i*_ data was calculated with the equation: *K*_*i*_ = IC_50_/(1 + (*F*/*K*_D_)); where IC_50_ was the concentration of the ligand that displaced the specific binding of the radioligand in a 50%; *K*_D_ is the dissociation constant of the radioligand, and *F* is the concentration of the radioligand employed in the assay.

### Functional Experiments

#### Cell Culture and Transient Transfection

HEK-293T cells were grown in Dulbecco’s Modified Eagle’s Medium (DMEM) medium (Gibco, Paisley, Scotland, United Kingdom) supplemented with 2 mM l-glutamine, 100 U/mL penicillin/streptomycin, MEM Non-Essential Amino Acids Solution (1/100) and 5% (v/v) heat inactivated Fetal Bovine Serum (FBS) (Invitrogen, Paisley, Scotland, United Kingdom). Cells were maintained in a humid atmosphere of 5% CO_2_ at 37 °C. Cells were transiently transfected with the PEI (Polyethylenimine, Sigma, St. Louis, MO, United States) method as previously described^71^ and used for functional assays 48 h later.

#### Neuronal Primary Cultures

To prepare primary neurons, brains from fetuses of pregnant CD1 mice were removed (gestational age: 19 days). Neurons were isolated as described in Hradsky et al.^72^ Briefly, after removal of the meninges, samples were dissected and digested with 0.25% trypsin (20 min at 37 °C). The effect of trypsin was stopped by adding an equal volume of culture medium (supplemented DMEM). A single-cell suspension was obtained by repeated pipetting followed by passage through a 100 μm-pore mesh. Pelleted (7 min, 200*g*) cells were resuspended in 2 mL of supplemented DMEM and seeded at a density of 3.5 × 10^5^ cells/mL in 6-well plates. After 24 h, the medium was replaced by neurobasal medium supplemented with 2 mM l-glutamine, 100 U/mL penicillin/streptomycin and 2% (v/v) B27 medium (GIBCO, Waltham, MA, USA). Primary neurons were assayed after 14 days in culture. Using NeuN as a marker, the percentage of neurons in the culture was >90%.

#### cAMP Determination

Signaling experiments have been performed as previously described.^73^ Two hours before initiating the experiment, HEK-293T cell-culture medium was replaced by serum-starved DMEM medium. Then, cells were detached, resuspended in growing medium containing 50 mM zardaverine (Tocris, Bristol, U.K.) and placed in 384-well microplates (2500 cells/well). Cells were pretreated (15 min) with cannabinoid compounds (1 nM to 100 μM) -or vehicle- before adding 0.5 mM forskolin (Tocris, Bristol, U.K.) to induce cAMP accumulation. Readings were performed after 15 min incubation at 25 °C. Homogeneous Time Resolved Fluorescence (HTRF) energy transfer measures were performed using the Lance Ultra cAMP kit (PerkinElmer, Waltham, MA, United States). Fluorescence at 665 nm was analyzed in a PHERA star Flagship microplate reader equipped with an HTRF optical module (BMG Lab Technologies, Offenburg, Germany).

#### Viability Assay

Primary cultures of striatal neurons were treated for 24 h with selected SCRAs (100 nM). Afterward, cells were scrapped from the plate and resuspended in neurobasal medium supplemented with 2 mM l-glutamine, 100 U/mL penicillin/streptomycin and 2% (v/v) B27 (GIBCO). Trypan blue staining was performed mixing 1 part of 0.4% trypan blue and 1 part of cell suspension in a plastic tube. After ∼3 min of incubation at room temperature, 10 μL of the mixture were sampled in a Neubauer chamber and counted with a Countess II FL (Life Technologies, California, CA, USA). The unstained (viable) and stained (nonviable) cells were counted separately, and the percentage of viability was calculated as total number of viable cells/total number of cells × 100.

#### Human Microsomal Stability

The human microsomal stability of selected compounds was evaluated by following a previously described method.^74^ The human microsomes employed were purchased from Tebu-Xenotech. The compound was incubated with the microsomes at 37 °C in a 50 mM phosphate buffer (pH = 7.4) containing 3 mM MgCl_2_, 1 mM NADP, 10 mM glucose-6-phosphate and 1 U/mL glucose-6-phosphate-dehydrogenase. Samples (75 μL) were taken from each well at 0, 10, 20, 40, and 60 min and transferred to a plate containing 4 °C 75 μL acetonitrile and 30 μL of 0.5% formic acid in water were added for improving the chromatographic conditions. The plate was centrifuged (46,000*g*, 30 min) and supernatants were taken and analyzed in an UPLC-MS/MS (Xevo-TQD, Waters) by employing a BEH C18 column and an isocratic gradient of 0.1% formic acid in water: 0.1% formic acid acetonitrile (60:40). The metabolic stability of the compounds was calculated from the logarithm of the remaining compounds at each of the time point studied.

#### Solubility Determinations

The solubility of selected compounds was evaluated by following a previously described method.^74^ A 10 mM stock solution of the compound was serially diluted in 100% DMSO and 2.5 μL of this solution was added to a 384-well UV-transparent plate (Greiner) containing 47.5 μL of PBS (pH = 7). The plate was incubated at 37 °C for 4 h and the light scattering was measured in a Nephelostar plus reader (BMG LABTECH). The data was fitted to a segmented linear regression for measuring the compound solubility.

#### Drug Transport Experiments

Caco-2 cells were grown in Dulbecco’s high-glucose modified eagle medium, composed of 10% fetal bovine serum, 2 mM of glutamine, 100 U/mL of penicillin, and 0.1 mg/mL of streptomycin (all components purchased from Corning, Milan, Italy) in a humidified incubator at 37 °C with a 5% CO_2_ atmosphere. The experiment started with the preparation of the Caco-2 monolayer, which occurred by seeding the cells (20,000/well) in Millicell plates (Millipore, Milan, Italy). Its growth was followed for 21 days by changing the medium occasionally and measuring its transepithelial electrical resistance (TEER) daily using an epithelial voltohmmeter (Millicell-ERS) until a TEER value >240 ohms × cm^2^ was reached. After 21 days, the plate was washed twice with Hank’s balanced salt solution (HBSS) (Invitrogen). After the second wash, the wells were filled with buffer, and the plate was kept at 37 °C for 30 min. After the incubation time, the HBSS buffer was replaced with the solutions of the compounds to be tested at a concentration of 1 × 10^–4^ M. The plates were placed in an incubator at 37 °C for 120 min. The apparent permeability (*P*_app_), in units of nm s^–1^, was calculated as previously reported.^75,76^

#### Calcein-AM Experiment

This experiment was carried out as described by Contino et al. with minor modifications.^77^ Each cell line (30,000 cells per well) was seeded into black CulturePlate 96/wells plate with 100 μL medium and allowed to become confluent overnight. 100 μL of test compounds were solubilized in culture medium and added to monolayers, with final concentrations ranging from 0.1 to 100 μM. 96/Wells plate was incubated at 37 °C for 30 min. Calcein-AM was added in 100 μL of Phosphate Buffered Saline (PBS) to yield a final concentration of 2.5 μM and plate was incubated for 30 min. Each well was washed 3 times with ice cold PBS. Saline buffer was added to each well and the plate was read with Victor3 (PerkinElmer) at excitation and emission wavelengths of 485 and 535 nm, respectively. In these experimental conditions Calcein cell accumulation in the absence and in the presence of tested compounds was evaluated and fluorescence basal level was estimated with untreated cells. In treated wells the increase of fluorescence with respect to basal level was measured. EC_50_ values were determined by fitting the fluorescence increase percentage versus log­[dose].

#### Anti-Inflammatory and Pro-Inflammatory Cytokine Detection

The amount of cytokines was measured in 5 μL of supernatants, derived from 1 × 104 cells, using the ProQuantum immunoassays kits for TNF-α, IFN-γ, IL-1β, IL-6, IL-10, and IL-4 [all from ThermoFisher Scientific (Waltham, MA)] according to the manufacturer’s instructions. The results were expressed as pg/mL based on the titration curved of each kits.

#### Cell Culture and Differentiation of SH-SY5Y Cells

SH-SY5Y human neuroblastoma-derived cells (ECACC, 94030304) were cultured and differentiated in black 384-well plates (Revvity, 6057300). Plates were treated with laminin (1:500 dilution in PBS; Merck, L2020) by adding 25 μL per well and incubated for 2 h at room temperature. Subsequently, plates were washed three times with PBS. Cells were seeded at a density of 6.6 × 10^3^ cells per well (in 50 μL) in culture medium. The base culture medium consisted of RPMI-1640 (Gibco, 61870) supplemented with 2% (v/v) fetal bovine serum (FBS; Merck, F7524), 1% (v/v) penicillin-streptomycin (P/S; Merck, P0781), and 0.1% (v/v) gentamicin (Merck, G1397).

Differentiation was initiated 24 h after plating. The culture medium was replaced with a differentiation medium composed of RPMI-1640 supplemented with 2% (v/v) FBS, 1% (v/v) P/S, 0.1% (v/v) gentamicin, and 10 μM all-trans retinoic acid (RA; Merck, R2625). Medium changes were performed every 3 days, and the plates were kept covered with aluminum foil to protect retinoic acid from light exposure. Six days later, cells were transfected with expression plasmids encoding *MAPT* P301L and *APP* V717I mutations employing Lipofectamine LTX (Thermo Fisher, 15338100) at a 1:2 proportion in Opti-MEM (Gibco, 11058-021) following the instructions of the manufacturer. After 6 h, the transfection medium was replaced with a differentiation medium composed of RPMI-1640, B27 supplement (1:50; Gibco, 17504), 1% (v/v) P/S, 0.1% (v/v) gentamicin, and 100 nM GLP-1 (Merck, G9416). 24 h later, test compounds were added using an Echo 650T acoustic dispenser (Labcyte) from a 10 mM stock solution in DMSO (Merck, D8418).

#### Fixation and Immunostaining

48 h after compound dispensation, cells were fixed with 4% paraformaldehyde (50 μL per well, Santa Cruz BT, sc-281692) for 20 min at 4 °C. Wells were then washed twice with HBSS (50 μL per wash, Merck, H8264) and incubated with 50 μL of blocking buffer for 30 min at room temperature. The blocking buffer consisted of HBSS supplemented with 5% BSA (w/v) (Merck, 10775835001) and 0.1% (v/v) Triton X-100 (Merck, T8787). After blocking, cells were incubated with an antibeta-tubulin antibody conjugated to Alexa Fluor 488 (1:500; BD Biosciences, 558605) and Draq5 nuclear stain (2.5 μM; Abcam, ab108410) diluted in blocking buffer. After incubation in the dark at room temperature, wells were washed once with 50 μL of HBSS and subsequently filled with 50 μL of blocking buffer prior to imaging.Images were acquired using an Operetta CLS High Content Screening System (Revvity) equipped with a 20× water-immersion objective. Fluorescent signals were detected using specific excitation/emission filter settings for each fluorophore: Alexa Fluor 488 (λ_ex_ = 460–490 nm; λ_em_ = 500–550 nm) and Draq5 (λ_ex_ = 615–645 nm; λ_em_ = 655–760 nm). For each well, nine nonoverlapping fields were captured under identical acquisition settings. Neurite length and complexity were quantified using Harmony software (version 4.9.2137.273; Revvity), applying a predefined analysis algorithm optimized for β-tubulin immunostaining. For each experiment, data were normalized to the average neurite length measured in the double-transfected condition (*MAPT* P301L + *APP* V717I) treated with vehicle, which was assigned a reference value of 100%. All other conditions were expressed as percentage relative to this baseline. Normalization was performed independently for each experimental replicate to control for interassay variability.

#### Data and Statistical Analysis

*K*_*i*_ and EC_50_ values were obtained by fitting the data with nonlinear regression using Prism 9 software (GraphPad, San Diego, CA). Results are the mean of four experiments (*n* = 4), each performed in duplicate. Data are represented as mean ± standard error of mean (SEM) with statistical significance set at *P* < 0.05. The number of samples (*n*) in each experimental condition is indicated in the corresponding figure legend. Outliers were assessed by the ROUT method,^57^ thus any sample was excluded assuming a *Q* value of 1% in GraphPad Prism 9. Comparisons among experimental groups were performed by Student’s *t* test or one-way analysis of variance (ANOVA) followed by Tukey’s multiple comparisons posthoc test using GraphPad Prism 9, as indicated.

#### Molecular Docking

Compounds **11**, **12**, **17e**, **18e**, **18f**, and **18o** were docked onto the recently published X-ray structure of CB_2_R in complex with the agonist AM12033 (co-x) (3.20 Å resolution, PDB code: 6KPC).^46^ The retrieved pdb file was prepared using the Protein Preparation Workflow from the Schrodinger Suite 2024-4,^78^ which added missing hydrogen atoms, reconstructed incomplete side chains, assigned favorable protonation states at physiological pH, and performed a force field-based minimization of the three-dimensional (3D) protein structure. Ligands were prepared using the LigPrep tool^78^ generating all the possible ionization states and tautomers at a pH of 7.0 ± 2.0. These prepared files were used for docking simulations carried out with the Grid-based Ligand Docking with Energetics (GLIDE) tool.^78^ Docking simulations employed SP mode with default settings, constructing a cubic grid centered on co-x, with an inner box size of 10.5 Å × 10.5 Å × 10.5 Å and an outer box size of 24.7 Å × 24.7 Å × 24.7 Å. To ensure thorough sampling of the ligands’ conformational space, we retained 50,000 poses per ligand during the initial docking phase (default value: 5000) and selected the top 4000 poses (default value: 400) per ligand for energy minimization. This protocol was evaluated by redocking the cognate ligand into the binding site. Satisfactorily, it returned to the original positions with a root-mean-square deviation (RMSD) of 0.71 Å, considering only heavy atoms. MM-GBSA Calculations. To account for potential conformational rearrangements in the protein binding site during molecular recognition and improve the accuracy of protein–ligand binding energy predictions, the top-ranked docking complexes were subjected to MM-GBSA calculations.^47^ The MM-GBSA method calculates the protein–ligand binding free energy for the obtained docking poses using the following equation:

$$\Delta G_{\mathrm{Bind}}\mathrm{MMGBSA}=E_{\mathrm{Complex}}-\left(E_{\mathrm{Receptor}}+E_{\mathrm{Ligand}}\right)$$

Where *E*_Complex_ represents the energy contribution of the optimized ligand–receptor complex, and *E*_Receptor_ and *E*_Ligand_ are the energy contributions of the optimized free receptor and free ligand, respectively. More negative Δ*G*_Bind_ values indicate stronger binding affinities. This method was applied using default dielectric constants, the OPLS4 force field,^79^ and the VSGB solvation model^80^ and allowed flexibility for residues within 3 Å of the ligand.

## Supplementary Material







## Notes

Spectroscopic and analytical data for all compounds described, HPLC enantiomeric separations, X-ray crystallography assays, supplementary figures, supplementary tables and HPLC traces for lead compounds (PDF)
Molecular formula strings (CSV)
Atomic coordinates and experimental data upon article publication (ZIP)

## Abbreviations Used

ABFE: absolute binding free energies
ADMET: absorption, distribution, metabolism, excretion and toxicity
Ado: adenosine
AGT: O^6^-alkylguanine-DNA alkyltransferase
AR: adenosine receptor
AUC: area under curve
cAMP: cyclic adenosine monophosphate
CD: circular dichroism
CFSE: carboxyfluorescein succinimidyl ester
CHO: Chinese hamster ovary
Cmpd: compound
Cmpds: compounds
DMEM: Dulbecco’s modified Eagle’s medium
DMF: dimethylformamide
DPCPX: Dipropylcyclopentyl-xanthine
FEP: Free Energy Perturbation
GPCRs: G protein-coupled receptors
MD: molecular dynamics
MFI: median fluorescence intensities
MPLC: medium pressure liquid chromatography
NECA: N-ethoxycarbonyl adenosine
NMR: nuclear magnetic resonance
P_1_: class 1 purinergic receptors
PAINS: pan-assay interference compounds
PBMCs: peripheral blood mononuclear cells
R-PIA: R-phenylisopropyl-adenosine
RBFE: relative binding free energies
ROESY: Rotating frame Overhauser Effect Spectroscopy
SAR: structure–activity relationships
SBC: spherical boundary conditions
SCAAS: surface-constrained all-atom solvent
SSR: structure-selectivity relationships
THF: tetrahydrofuran
Veh: vehicle
