# Supplementary material for: Conformational Restriction of Designer Drugs Reveals Subtype-Selective and Biased CB2 Agonists with Neuroprotective Effects
Source: J Med Chem. 2025 Aug 12;68(16):17103–29. doi: 10.1021/acs.jmedchem.5c00604 (PMC12406204; doi:10.1021/acs.jmedchem.5c00604)
Supplement: Supplementary file 1 [file jm5c00604_si_001.pdf]

SUPPORTING INFORMATION

Conformational Restriction of Designer Drugs Reveals Subtype-Selective and Biased CB_2 Agonists with Neuroprotective Effects

Claudia Gioé-Gallo,[1Ψ] Sandra Ortigueira,[1Ψ] Rubén Prieto-Díaz,[1] Marialessandra Contino,[2] Jhonny Azuaje,[1] Maria Grazia Perrone,[2] Chiara Riganti,[3] Domenico Alberga,[4] Giuseppe Felice Mangiatordi,[4] Antonio Andújar-Arias,[1] Aitor García-Rey,[1] Giovanni Graziano,[1] Angela Stefanachi,[2] Cristina Val,[5] Antón Leandro-Martínez,[5] Joan Biel Rebassa,[6] David Reza,[1] Asier Selas,[1] Fabio Francavilla,[1] M. Rita Paleo,[1] Xerardo García-Mera,[1] M. Isabel Loza,[5] Gemma Navarro,[6*] José Brea,[5*] and Eddy Sotelo[1*]

[1]Centro Singular de Investigación en Química Biolóxica e Materiais Moleculares (CiQUS), Universidade de Santiago de Compostela, 15782. Santiago de Compostela, Spain. [2]Dipartimento di Farmacia-Scienze del Farmaco, Università degli Studi di Bari ALDO MORO, via Orabona 4, 70125, Bari, Italy. [3]Dipartimento di Oncologia, Università degli Studi di Torino, 10124 Torino, Italy. [4]CNR – Institute of Crystallography, Via Giovanni Amendola, 122/O, 70126, Bari, Italy. [5]Centro Singular de Investigación en Medicina Molecular y Enfermedades Crónicas (CiMUS). Universidade de Santiago de Compostela, 15782. Santiago de Compostela, Spain. [6]Department of Biochemistry and Physiology, School of Pharmacy and Food Science, Universitat de Barcelona, Barcelona 08028, Spain.

*To whom correspondence should be addressed:
(ES) +34 881815732, e-mail: e.sotelo@usc.es
(JMB) +34 881815459, e-mail: pepo.brea@usc.es
(GN) +34 934024505, e-mail: g.navarro@ub.edu
[Ψ]These authors contributed equally to this work and share first authorship.

TABLE OF CONTENTS

Molecular docking simulations on CB_1R **S3**
Prediction and comparative analysis of molecular descriptors impacting drug-like properties. **S4**
General procedures for synthesis. **S5**
Spectroscopic and analytical data for compounds described. **S7**
Circular Dichroism **S27**
HPLC traces of representative enantiopure esters and amides. **S28**
HPLC purity analysis **S43**
Supplementary Figures **S57**
References **S62**

Chemistry. General Information.

All starting materials, reagents and solvents were purchased (Sigma Aldrich) and used without further purification. After extraction from aqueous phases, the organic solvents were dried over anhydrous magnesium sulphate. The reactions were monitored by thin-layer chromatography (TLC) on 2.5 mm Merck silica gel GF 254 strips, and the purified compounds each showed a single spot. Unless stated otherwise, UV light, phosphomolybdic acid or iodine vapor were used to detect compounds. The amide coupling reactions were performed in coated Kimble vials on a PLS (6×4) Organic Synthesizer with orbital stirring. The purity and identity of all tested compounds were established by a combination of high-performance liquid chromatography (HPLC), mass spectrometry and nuclear magnetic resonance (NMR) spectroscopy. Purification of isolated products was carried out by column chromatography (Kieselgel 0.040–0.063 mm, E. Merck) or preparative chromatography (Uniplate™ Techware 20x20 cm Silica G 1000mm Thick 15µm).

The NMR spectra were recorded on Bruker AM300 and XM500 spectrometers. Chemical shifts are given as δ values against tetramethylsilane as internal standard and *J* values are given in Hz. The signal multiplicity shown with the abbreviatures s, singlet; d, doublet; t, triplet; dd, doublet of doublets; dt, doublet of triplets; td, triplet of doublets; and m, multiplet, followed by number of protons (deduced by integration). The coupling constants (*J*) are deduced in Hertz (Hz).

Mass spectra were obtained on a Varian MAT-711 instrument. High-resolution mass spectra were obtained on an Autospec Micromass spectrometer. Analytical HPLC and Low-resolution mass spectra were performed on a Waters™ Acquity™ Arc™ UHPLC System using a XBridge® C18, 4.6 mm × 50 mm, 3.5 µm column with gradient elution using the mobile phases (A) H_2O containing 0.1% HCOOH and (B) MeCN containing 0.1% HCOOH and a flow rate of 1 mL/min. The purity of all tested compounds was determined to be >95%.

The chiral analysis was performed using an Agilent 1100 Series HPLC Value System (G1322A Degasser; G1311A Quaternary Pump; G1314A VWD Detector; G1313A ALS Autosampler). Enantiomers were evaluated with 250 mm x 20 mm Chiralpak® 5µm IE-3 (DAICEL) using an isocratic mobile phase *n*-hexane/*i*-propanol 80:20.

CD spectra were recorded on a Jasco-815 system equipped with a Peltier-type thermostatic accessory (CDF-426S, Jasco). Measurements were carried out at 20 ºC using a 1-mm quartz cell in a volume of 300–350 ml. Compounds (0.1 mg) were dissolved in MeOH (1.0 ml). The instrument settings were bandwidth, 1.0 nm; data pitch, 1.0 nm; speed, 500 nm/min; accumulation, 10; wavelengths, 400–190 nm). A detailed description of analytical and spectroscopic data for all described compounds is described below.

Molecular docking simulations on CB_1R

Compound **13** was docked onto the X-ray crystal structure of CB_1R in complex with the agonist AM11542 (2.80 Å resolution, PDB code: 5XRA,[1] The protein structure was prepared using the Protein Preparation Workflow from the Schrödinger Suite 2024-4, which added missing hydrogen atoms, rebuilt incomplete side chains, assigned the most favorable protonation states at physiological pH, and applied a force field-based minimization to optimize the 3D conformation. Ligand preparation was performed with the LigPrep tool[2] generating all relevant ionization states and tautomers within a pH range of 7.0 ± 2.0. These processed structures were then used in docking simulations conducted with the Grid-based Ligand Docking with Energetics (GLIDE) tool.[2] Docking simulations were carried out in SP mode with default settings, using a cubic grid centered on co-x, with an inner box dimension of 10.3 Å × 10.3 Å × 10.3 Å and an outer box of 24.3 Å × 24.3 Å × 24.3 Å. To ensure comprehensive exploration of ligand conformations, the initial docking phase retained 50,000 poses per ligand (default: 5,000), selecting the top 4,000 (default: 400) for energy minimization. The accuracy of this protocol was assessed by redocking the cognate ligand into its binding site, yielding an RMSD of 0.27 Å for heavy atoms, confirming an accurate pose reproduction. To account for potential conformational rearrangements in the protein binding site and improve the reliability of binding energy predictions, the top-ranked docking complex underwent MM-GBSA calculations. The computed binding free energy of -61.37 kcal/mol aligned well with the experimentally measured CB_1R affinity (K_i = 268 nM). **Figure S3** shows the complex obtained from the MM-GBSA refinement of the highest-ranked pose of compound **13**, which is predicted to interact through well-oriented π-π interactions with F170 and F174. Interestingly, hydrogen bonding interactions are also predicted with the backbone of K376 and the side chain of H178. Further visual inspection suggests that the conformational restriction applied in this study may introduce steric hindrance due to the proximity of F177 to the non-terminal amide group. To

evaluate this hypothesis, we applied the same protocol used applied to **18e**. It is worth noting that the two compounds differ only in the applied conformational restriction and in the presence of a second nitrogen atom in the core structure. Consistent with our hypothesis, the application of the described protocol did not yield any docking poses. Specifically, to assess whether **18e** could bind CB_1R in the same conformation as **13**, the conformational space exploration was restricted to poses with an RMSD lower than 0.8 Å relative to the pose obtained for **13** (based on the overlay of all heavy atoms). In conclusion, the obtained data strongly suggest that the applied conformational restriction prevents **18e** from efficiently binding CB_1R, in agreement with experimental findings.

Prediction and comparative analysis of molecular descriptors impacting drug-like properties.

Pharmacokinetic properties were assessed using a combination of computational tools and visualized through radar and BOILED-Egg plots. Furthermore, the CNS multiparameter optimization (CNS MPO) score was calculated using ChemAxon InstantJChem,[3] evaluating the compounds' potential for central nervous system exposure based on factors such as LogP, molecular weight, TPSA, and hydrogen bond donors. The radar plots were generated using the Matplotlib library in Python, based on the bioavailability criteria provided by SwissADME.[4] These plots include key parameters such as lipophilicity (LogP), molecular weight, topological polar surface area (TPSA), number of rotatable bonds, number of heavy atoms, and solubility. The blue hexagon in the radar plots represents the optimal bioavailability zone, allowing for an easy comparison of the compounds' pharmacokinetic profiles to ideal ranges. In addition, the BOILED-Egg diagram was constructed using the parameters outlined in the original BOILED-Egg publication,[5] combined with the specific properties of the studied compounds. This plot predicts passive gastrointestinal absorption and brain penetration based on the compounds' positions within predefined regions, providing a straightforward visualization of their potential for oral absorption and CNS exposure.

General procedures for synthesis.

General procedure for the synthesis of esters 15a-p and **16a-p**: A mixture of the 1-substituted indole (or indazole)-3-carboxylic acid (1 mmol) (**19**), the corresponding cyclic amino ester (**20**) (1.5 mmol), HATU (1.5 mmol) and DIPEA (4 mmol) in CH_2Cl_2 (2 mL) was stirred with orbital stirring at room temperature for 24h. After completion of the reaction, water was added, and the mixture was extracted with CH_2Cl_2. The organic phase was dried over $MgSO_4$, filtered, and concentrated. The resulting product was purified by column chromatography on silica gel. The purity of all prepared compounds was established by high-performance liquid chromatography (HPLC) and showed to be >95%.

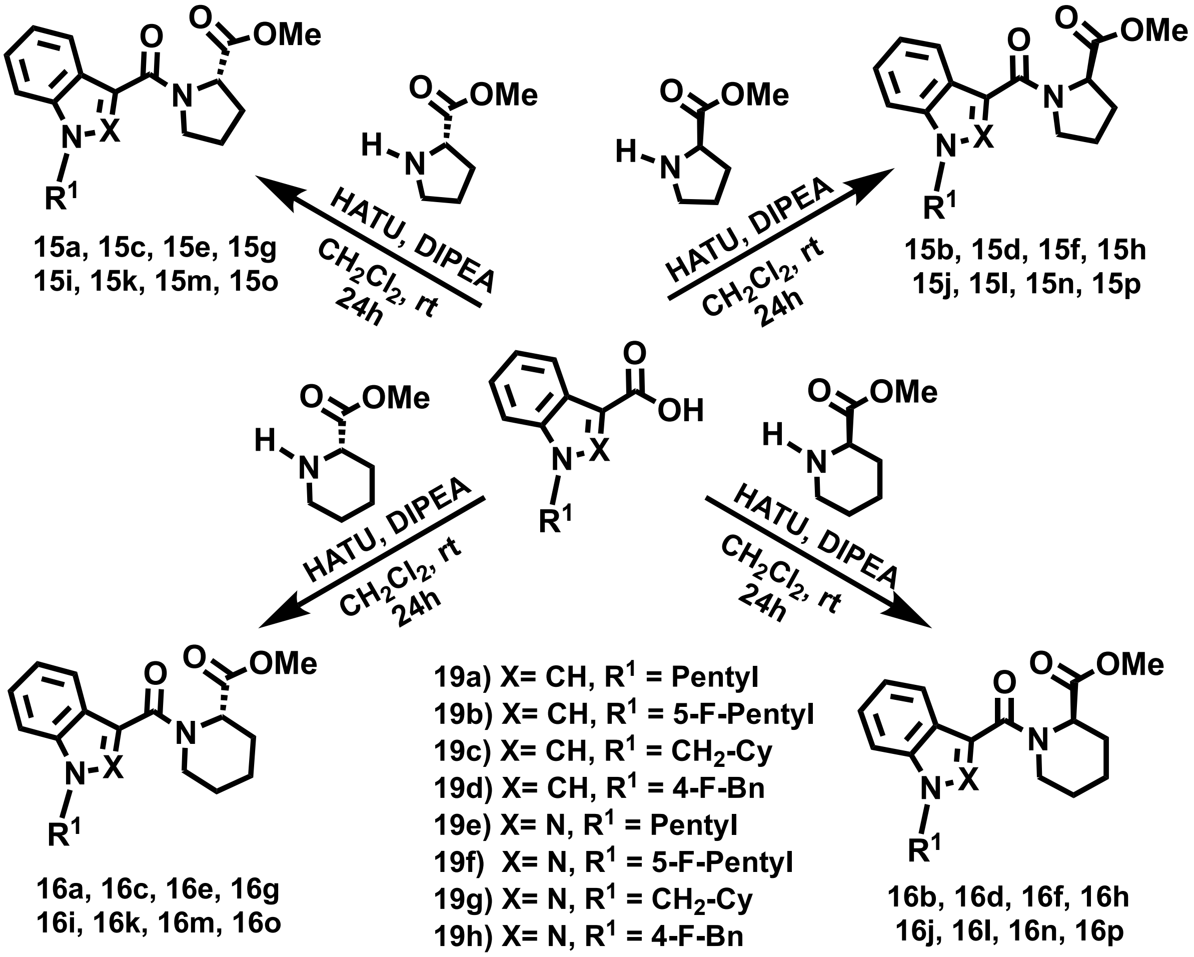

General procedures for the synthesis of amides 17a-p and 18a-p. A mixture of the corresponding ester (**15** or **16**), and a solution 4 molar of ammonia (4 mmol) in MeOH (2 mL) was stirred at room temperature for 4h. After completion of the reaction, solvent was evaporated. The resulting product was purified by column chromatography on silica gel using MeOH/CH_2Cl_2. The purity of all prepared compounds was established by high-performance liquid chromatography (HPLC) and showed to be >95%.

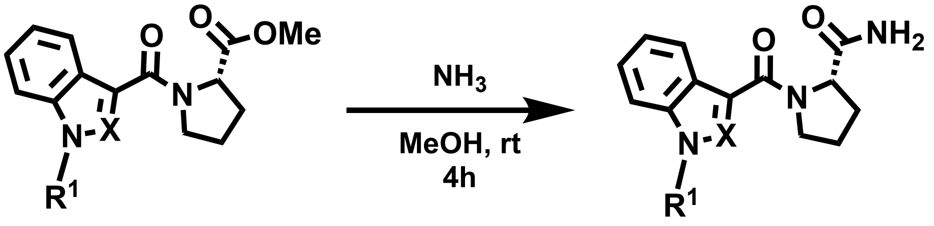

15a, 15c, 15e, 15g
15i, 15k, 15m, 15o

17a, 17c, 17e, 17g
17i, 17k, 17m, 17o

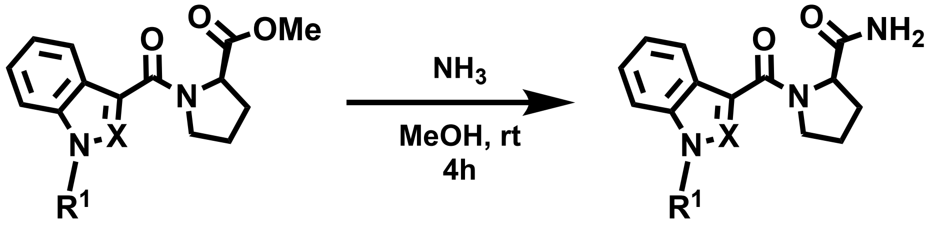

15b, 15d, 15f, 15h
15j, 15l, 15n, 15p

17b, 17d, 17f, 17h
17j, 17l, 17n, 17p

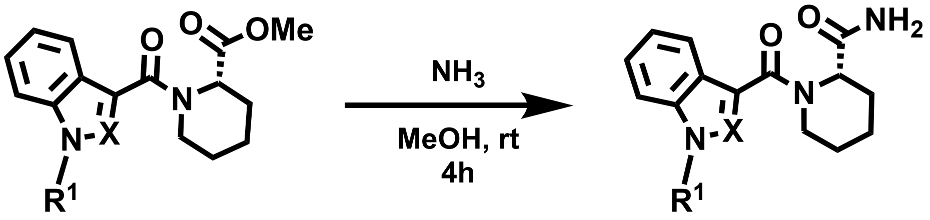

16a, 16c, 16e, 16g
16i, 16k, 16m, 16o

18a, 18c, 18e, 18g
18i, 18k, 18m, 18o

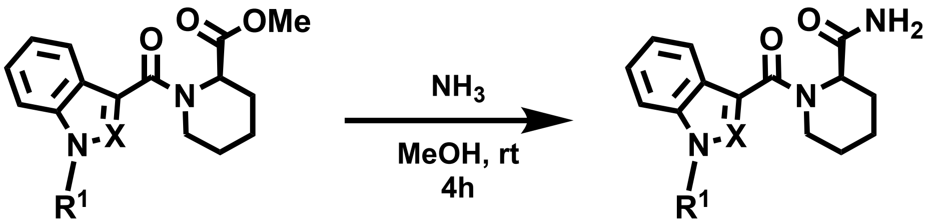

16b, 16d, 16f, 16h
16j, 16l, 16n, 16p

18b, 18d, 18f, 18h
18j, 18l, 18n, 18p

Spectroscopic and analytical data for compounds described.

(*S*)-Methyl (1-pentyl-1*H*-indole-3-carbonyl)prolinate (15a). Yield: 79%. ^{1}H NMR (300 MHz, $CDCl_3$) δ (ppm): 8.14 (d, *J* = 7.7 Hz, 1H, Ar-H), 7.44 (s, 1H, Ar-H), 7.34 – 7.13 (m, 3H, Ar-H), 4.74 (dd, *J* = 8.1, 5.1 Hz, 1H, CH), 4.10 (t, *J* = 7.2 Hz, 2H, $CH_2CH_2CH_2CH_2CH_3$), 3.92 – 3.77 (m, 2H, Pro), 3.73 (s, 3H, OCH_3), 2.36 – 2.21 (m, 1H, Pro), 2.10 – 1.91 (m, 2H, Pro), 1.89 – 1.77 (m, 3H, Pro, $CH_2CH_2CH_2CH_2CH_3$), 1.38 – 1.23 (m, 4H, $CH_2CH_2CH_2CH_2CH_3$, $CH_2CH_2CH_2CH_2CH_3$), 0.87 (t, *J* = 6.8 Hz, 3H, $CH_2CH_2CH_2CH_2CH_3$). LRMS (ESI) *m/z* calcd. for $C_{20}H_{27}N_2O_3$ $[M+H]^+$: 343.2; found: 343.2.

(*R*)-Methyl (1-pentyl-1*H*-indole-3-carbonyl)prolinate (15b). Yield: 75%. ^{1}H NMR (300 MHz, $CDCl_3$) δ (ppm): 8.17 (d, *J* = 7.8 Hz, 1H, Ar-H), 7.48 (s, 1H, Ar-H), 7.39 – 7.18 (m, 3H, Ar-H), 4.78 (dd, *J* = 8.0, 5.1 Hz, 1H, CH), 4.15 (t, *J* = 7.2 Hz, 2H, $CH_2CH_2CH_2CH_2CH_3$), 3.95 – 3.82 (m, 2H, Pro), 3.77 (s, 3H, OCH_3), 2.40 – 2.27 (m, 1H, Pro), 2.18 – 1.97 (m, 2H, Pro), 1.95 – 1.78 (m, 3H, Pro, $CH_2CH_2CH_2CH_2CH_3$), 1.45 – 1.29 (m, 4H, $CH_2CH_2CH_2CH_2CH_3$, $CH_2CH_2CH_2CH_2CH_3$), 0.91 (t, *J* = 6.7 Hz, 3H, $CH_2CH_2CH_2CH_2CH_3$). LRMS (ESI) *m/z* calcd. for $C_{20}H_{27}N_2O_3$ $[M+H]^+$: 343.2; found: 343.2.

(*S*)-Methyl (1-(5-fluoropentyl)-1*H*-indole-3-carbonyl)prolinate (15c). Yield: 72%. ^{1}H NMR (300 MHz, $CDCl_3$) δ (ppm): 8.16 (d, *J* = 7.7 Hz, 1H, Ar-H), 7.46 (s, 1H, Ar-H), 7.36 – 7.16 (m, 3H, Ar-H), 4.75 (dd, *J* = 8.1, 5.0 Hz, 1H, CH), 4.50 (t, *J* = 5.9 Hz, 1H, $CH_2CH_2CH_2CH_2CH_2F$), 4.34 (t, *J* = 5.9 Hz, 1H, $CH_2CH_2CH_2CH_2CH_2F$), 4.15 (t, *J* = 7.0 Hz, 2H, $CH_2CH_2CH_2CH_2CH_2F$), 3.93 – 3.79 (m, 2H, Pro), 3.75 (s, 3H, OCH_3), 2.39 – 2.22 (m, 2H, $CH_2CH_2CH_2CH_2CH_2F$), 2.15 – 1.83 (m, 4H, Pro, $CH_2CH_2CH_2CH_2CH_2F$), 1.81 – 1.60 (m, 2H, Pro), 1.54 – 1.38 (m, 2H, $CH_2CH_2CH_2CH_2CH_2F$). LRMS (ESI) *m/z* calcd. for $C_{20}H_{26}FN_2O_3$ $[M+H]^+$: 361.2; found: 361.2.

(*R*)-Methyl (1-(5-fluoropentyl)-1*H*-indole-3-carbonyl)prolinate (15d). Yield: 74%. ^{1}H NMR (300 MHz, $CDCl_3$) δ (ppm): 8.18 (d, *J* = 7.4 Hz, 1H, Ar-H), 7.47 (s, 1H, Ar-H), 7.38 – 7.17 (m, 3H, Ar-H), 4.77 (dd, *J* = 8.1, 5.2 Hz, 1H, CH), 4.51 (t, *J* = 5.9 Hz, 1H, $CH_2CH_2CH_2CH_2CH_2F$), 4.36 (t, *J* = 5.9 Hz, 1H, $CH_2CH_2CH_2CH_2CH_2F$), 4.17 (t, *J* = 7.1 Hz, 2H, $CH_2CH_2CH_2CH_2CH_2F$), 3.96 – 3.81 (m, 2H, Pro), 3.77 (s, 3H, OCH_3), 2.41 – 2.24 (m, 2H, $CH_2CH_2CH_2CH_2CH_2F$), 2.17 – 1.88 (m, 4H, Pro, $CH_2CH_2CH_2CH_2CH_2F$), 1.83 – 1.61 (m, 2H, pro), 1.55 – 1.40 (m, 2H, $CH_2CH_2CH_2CH_2CH_2F$). LRMS (ESI) *m/z* calcd. for $C_{20}H_{26}FN_2O_3$ $[M+H]^+$: 361.2; found: 361.2.

(*S*)-Methyl (1-(cyclohexylmethyl)-1*H*-indole-3-carbonyl)prolinate (15e). Yield: 84%. ^{1}H NMR (300 MHz, $CDCl_3$) δ (ppm): 8.12 (d, *J* = 7.6 Hz, 1H, Ar-H), 7.37 (s, 1H, Ar-H), 7.31 – 7.10 (m, 3H, Ar-H), 4.70 (dd, *J* = 7.9, 4.9 Hz, 1H, CH), 3.90 (d, *J* = 6.8 Hz, 2H, C*H*$_2$-Cy), 3.86 – 3.75 (m, 2H, Pro), 3.70 (s, 3H, OCH_3), 2.32 – 2.16 (m, 1H, C*H*-Cy), 2.13 – 1.74 (m, 4H, Cy, Pro), 1.71 – 1.49 (m, 4H, Cy), 1.25 – 1.04 (m, 4H, Cy, Pro), 1.03 – 0.85 (m, 2H, Cy). LRMS (ESI) *m/z* calcd. for $C_{22}H_{29}N_2O_3$ $[M+H]^+$: 369.2; found: 369.2.

(*R*)-Methyl (1-(cyclohexylmethyl)-1*H*-indole-3-carbonyl)prolinate (15f). Yield: 77%. ^{1}H NMR (300 MHz, $CDCl_3$) δ (ppm): 8.12 (d, *J* = 7.6 Hz, 1H, Ar-H), 7.37 (s, 1H, Ar-H), 7.31 – 7.10 (m, 3H, Ar-H), 4.70 (dd, *J* = 7.9, 4.8 Hz, 1H, CH), 3.89 (d, *J* = 7.1, 2H, C*H*$_2$-Cy), 3.85 – 3.75 (m, 2H, Pro), 3.71 (s, 3H, OCH_3), 2.34 – 2.19 (m, 1H, C*H*-Cy), 2.16 – 1.74 (m, 4H, Cy, Pro), 1.74 – 1.52 (m, 4H, Cy), 1.24 – 1.06 (m, 4H, Cy, Pro), 1.05 – 0.83 (m, 2H, Cy). ^{13}C NMR (75 MHz, $CDCl_3$) δ (ppm): 173.55, 165.53, 136.24, 130.67, 127.71, 122.64, 122.40, 121.12, 110.13, 109.83, 59.82, 53.44, 52.28, 49.04, 38.74, 31.13, 29.55, 26.32, 25.76, 25.42. HRMS (ESI) *m/z* calcd. for $C_{22}H_{29}N_2O_3$ $[M+H]^+$: 369.2173; found: 369.2173.

(*S*)-Methyl (1-(4-fluorobenzyl)-1*H*-indole-3-carbonyl)prolinate (15g). Yield: 88%. ^{1}H NMR (300 MHz, $CDCl_3$) δ (ppm): 8.21 – 8.11 (m, 1H, Ar-H), 7.47 (s, 1H, Ar-H), 7.25 – 7.20 (m, 3H, Ar-H), 7.14 – 7.06 (m, 2H, Ar-H), 7.04 – 6.96 (m, 2H, Ar-H), 5.30 (s, 2H, C*H*$_2$-Ph), 4.74 (dd, *J* = 8.0, 5.3 Hz, 1H, CH), 3.88 – 3.77 (m, 2H, Pro), 3.74 (s, 3H, OCH_3), 2.39 – 2.24 (m, 1H, Pro), 2.17 – 1.88 (m, 3H, Pro). LRMS (ESI) *m/z* calcd. for $C_{22}H_{22}FN_2O_3$ $[M+H]^+$: 381.2; found: 381.2.

(*R*)-Methyl (1-(4-fluorobenzyl)-1*H*-indole-3-carbonyl)prolinate (15h). Yield: 81%. ^{1}H NMR (300 MHz, $CDCl_3$) δ (ppm): 8.27 – 8.18 (m, 1H, Ar-H), 7.49 (s, 1H, Ar-H), 7.25 – 7.20 (m, 3H, Ar-H), 7.14 – 7.06 (m, 2H, Ar-H), 7.06 – 6.97 (m, 2H, Ar-H), 5.30 (s, 2H, CH_2-Ph), 4.74 (dd, *J* = 8.1, 4.9 Hz, 1H, CH), 3.89 – 3.80 (m, 2H, Pro), 3.78 (s, 3H, OCH_3), 2.44 – 2.24 (m, 1H, Pro), 2.23 – 1.88 (m, 3H, Pro). ^{13}C NMR (75 MHz, $CDCl_3$) δ (ppm): 173.48, 165.33, 162.55 (d, *J* = 246.7 Hz), 136.11, 132.29 (d, *J* = 3.2 Hz), 130.05, 128.74 (d, *J* = 8.2 Hz), 127.89, 123.17, 122.54, 121.59, 116.01 (d, *J* = 21.7 Hz), 111.30, 109.81, 59.84, 52.33, 49.90, 49.07, 29.54, 25.47. HRMS (ESI) *m/z* calcd. for $C_{22}H_{22}FN_2O_3$ $[M+H]^+$: 381.1609; found: 381.1609.

(*S*)-Methyl (1-pentyl-1*H*-indazole-3-carbonyl)prolinate (15i). Yield: 72%. ^{1}H NMR (300 MHz, $CDCl_3$) δ (ppm): 8.41 (d, *J* = 7.9 Hz, 1H, Ar-H), 7.46 – 7.33 (m, 2H, Ar-H), 7.32 – 7.17 (m, 1H, Ar-H), 5.32 (dd, *J* = 8.4, 3.1 Hz, 1H, CH), 4.37 (t, *J* = 6.9 Hz, 1H, $CH_2CH_2CH_2CH_2CH_3$), 4.29 (t, *J* = 6.4 Hz, 1H, $CH_2CH_2CH_2CH_2CH_3$), 4.26 – 4.13 (m, 1H, Pro), 4.01 – 3.81 (m, 1H, Pro), 3.78 (s, 3H, OCH_3), 2.44 – 2.12 (m, 1H, Pro), 2.10 – 1.83 (m, 5H, Pro, $CH_2CH_2CH_2CH_2CH_3$), 1.44 – 1.18 (m, 4H, $CH_2CH_2CH_2CH_2CH_3$, $CH_2CH_2CH_2CH_2CH_3$), 1.00 – 0.79 (m, 3H, $CH_2CH_2CH_2CH_2CH_3$). LRMS (ESI) *m/z* calcd. for $C_{19}H_{26}N_3O_3$ $[M+H]^+$: 344.2; found: 344.2.

(*R*)-Methyl (1-pentyl-1*H*-indazole-3-carbonyl)prolinate (15j). Yield: 80%. ^{1}H NMR (300 MHz, $CDCl_3$) δ (ppm): 8.40 (d, *J* = 8.2 Hz, 1H, Ar-H), 7.47 – 7.31 (m, 2H, Ar-H), 7.31 – 7.16 (m, 1H, Ar-H), 5.32 (dd, *J* = 8.5, 3.0 Hz, 1H, CH), 4.37 (t, *J* = 7.0 Hz, 1H, $CH_2CH_2CH_2CH_2CH_3$), 4.29 (t, *J* = 6.6 Hz, 1H, $CH_2CH_2CH_2CH_2CH_3$), 4.25 – 4.12 (m, 1H, Pro), 4.00 – 3.80 (m, 1H, Pro), 3.77 (s, 3H, OCH_3), 2.44 – 2.09 (m, 1H, Pro), 2.11 – 1.76 (m, 5H, Pro, $CH_2CH_2CH_2CH_2CH_3$), 1.47 – 1.16 (m, 4H, $CH_2CH_2CH_2CH_2CH_3$, $CH_2CH_2CH_2CH_2CH_3$), 1.01 – 0.79 (m, 3H, $CH_2CH_2CH_2CH_2CH_3$). ^{13}C NMR (75 MHz, $CDCl_3$) δ (ppm): 173.51, 162.58, 139.07, 129.58, 126.65, 124.75, 123.55, 122.37, 108.95, 60.71, 52.19, 49.45, 47.62, 32.01, 29.33, 28.96, 25.59, 22.21, 14.00. HRMS (ESI) *m/z* calcd. for $C_{19}H_{26}N_3O_3$ $[M+H]^+$: 344.1969; found: 344.1969.

(*S*)-Methyl (1-(5-fluoropentyl)-1*H*-indazole-3-carbonyl)prolinate (15k). Yield: 78%. ^{1}H NMR (300 MHz, $CDCl_3$) δ (ppm): 8.35 (d, *J* = 8.0 Hz, 1H, Ar-H), 7.43 – 7.33 (m, 2H, Ar-H), 7.29 – 7.18 (m, 1H, Ar-H), 5.30 (dd, *J* = 8.8, 2.7 Hz, 1H, CH), 4.47 (t, *J* = 6.0 Hz, 1H, $CH_2CH_2CH_2CH_2C\mathit{H_2}F$), 4.40 (t, *J* = 6.9 Hz, 1H, $CH_2CH_2CH_2CH_2C\mathit{H_2}F$), 4.32 (t, *J* = 6.2 Hz, 2H, $C\mathit{H_2}CH_2CH_2CH_2CH_2F$), 4.27 – 4.06 (m, 1H, Pro), 3.99 – 3.80 (m, 1H, Pro), 3.76 (s, 3H, OCH_3), 2.42 – 2.09 (m, 2H, $CH_2CH_2CH_2C\mathit{H_2}CH_2F$), 2.09 – 1.89 (m, 4H, Pro, $CH_2C\mathit{H_2}CH_2CH_2CH_2F$), 1.83 – 1.59 (m, 2H, Pro), 1.53 – 1.32 (m, 2H, $CH_2CH_2C\mathit{H_2}CH_2CH_2F$). LRMS (ESI) *m/z* calcd. for $C_{19}H_{25}FN_3O_3$ $[M+H]^+$: 362.2; found: 362.2.

(*R*)-Methyl (1-(5-fluoropentyl)-1*H*-indazole-3-carbonyl)prolinate (15l). Yield: 73%. ^{1}H NMR (300 MHz, $CDCl_3$) δ (ppm): 8.41 (d, *J* = 8.2 Hz, 1H, Ar-H), 7.48 – 7.33 (m, 2H, Ar-H), 7.32 – 7.14 (m, 1H, Ar-H), 5.31 (dd, *J* = 8.7, 2.7 Hz, 1H, CH), 4.48 (t, *J* = 6.0 Hz, 1H, $CH_2CH_2CH_2CH_2C\mathit{H_2}F$), 4.40 (t, *J* = 6.9 Hz, 1H, $CH_2CH_2CH_2CH_2C\mathit{H_2}F$), 4.32 (t, *J* = 6.2 Hz, 2H, $C\mathit{H_2}CH_2CH_2CH_2CH_2F$), 4.28 – 4.11 (m, 1H, Pro), 4.00 – 3.80 (m, 1H, Pro), 3.77 (s, 3H, OCH_3), 2.44 – 2.08 (m, 2H, Pro), 2.08 – 1.89 (m, 4H, Pro, $CH_2C\mathit{H_2}CH_2CH_2CH_2F$), 1.84 – 1.58 (m, 2H, Pro), 1.56 – 1.29 (m, 2H, $CH_2CH_2C\mathit{H_2}CH_2CH_2F$). ^{13}C NMR (75 MHz, $CDCl_3$) δ (ppm): 173.50, 162.53, 139.17, 126.80, 124.76, 123.62, 122.48, 108.85, 83.78 (d, *J* = 164.7 Hz), 60.72, 52.21, 49.33, 47.63, 32.03, 30.03 (d, *J* = 19.7 Hz), 29.12, 25.59, 22.78 (d, *J* = 5.1 Hz), 22.10. HRMS (ESI) *m/z* calcd. for $C_{19}H_{25}FN_3O_3$ $[M+H]^+$: 362.1874; found: 362.1877.

(*S*)-Methyl (1-(cyclohexylmethyl)-1*H*-indazole-3-carbonyl)prolinate (15m). Yield: 82%. ^{1}H NMR (300 MHz, $CDCl_3$) δ (ppm): 8.39 (d, *J* = 8.2 Hz, 1H, Ar-H), 7.44 – 7.29 (m, 2H, Ar-H), 7.29 – 7.14 (m, 1H. Ar-H), 4.73 (dd, *J* = 8.3, 4.0 Hz, 1H, CH), 4.14 – 4.04 (m, 2H, $C\mathit{H_2}$-Cy), 3.88 – 3.78 (m, 2H, Pro), 3.64 (s, 3H, OCH_3), 2.36 – 2.18 (m, 1H, C*H*-Cy), 2.10 – 1.86 (m, 4H, Cy, Pro), 1.75 – 1.47 (m, 4H, Cy, Pro), 1.29 – 1.09 (m, 4H, Cy, Pro), 1.09 – 0.90 (m, 2H, Cy). LRMS (ESI) *m/z* calcd. for $C_{21}H_{28}N_3O_3$ $[M+H]^+$: 370.2; found: 370.2.

(*R*)-Methyl (1-(cyclohexylmethyl)-1*H*-indazole-3-carbonyl)prolinate (15n). Yield: 90%. ^{1}H NMR (500 MHz, DMSO-d_6) δ (ppm): 8.13 (d, *J* = 8.3 Hz, 1H, Ar-H), 7.71 – 7.61 (m, 1H, Ar-H), 7.41 – 7.32 (m, 1H, Ar-H), 7.22 – 7.14 (m, 1H, Ar-H), 4.53 (dd, *J* = 8.6, 4.9 Hz, 1H, CH), 4.16 (d, *J* = 7.1 Hz, 2H, CH_2-Cy), 3.63 (dd, *J* = 8.5, 5.5 Hz, 2H, Pro), 3.55 (s, 3H, OCH_3), 2.35 – 2.14 (m, 1H, C*H*-Cy), 2.08 – 1.71 (m, 4H, Cy, Pro), 1.63 – 1.37 (m, 4H, Cy, Pro), 1.15 – 1.03 (m, 4H, Cy, Pro), 1.02 – 0.91 (m, 2H, Cy). ^{13}C NMR (75 MHz, $CDCl_3$) δ (ppm): 173.79, 162.65, 140.55, 126.71, 126.57, 123.54, 122.43, 122.26, 109.20, 60.73, 55.77, 52.22, 48.58, 38.74, 32.06, 31.03, 26.36, 25.71, 22.10. HRMS (ESI) *m/z* calcd. for $C_{21}H_{28}N_3O_3$ $[M+H]^+$: 370.2125; found: 370.2128.

(*S*)-Methyl (1-(4-fluorobenzyl)-1*H*-indazole-3-carbonyl)prolinate (15o). Yield: 89%. ^{1}H NMR (300 MHz, $CDCl_3$) δ (ppm): 8.41 (d, *J* = 8.2 Hz, 1H, Ar-H), 7.39 – 7.10 (m, 5H, Ar-H), 7.02 – 6.90 (m, 2H, Ar-H), 5.46 (s, 2H, CH_2-Ph), 4.74 (dd, *J* = 8.5, 4.1 Hz, 1H, CH), 4.02 – 3.79 (m, 2H, Pro), 3.76 (s, 3H, OCH_3), 2.40 – 1.88 (m, 4H, Pro). LRMS (ESI) *m/z* calcd. for $C_{21}H_{21}FN_3O_3$ $[M+H]^+$: 382.2; found: 382.2.

(*R*)-Methyl (1-(4-fluorobenzyl)-1*H*-indazole-3-carbonyl)prolinate (15p). Yield: 81%. ^{1}H NMR (300 MHz, $CDCl_3$) δ (ppm): 8.42 (d, *J* = 8.2 Hz, 1H, Ar-H), 7.45 – 7.09 (m, 5H, Ar-H), 7.09 – 6.88 (m, 2H, Ar-H), 5.47 (s, 2H, CH_2-Ph), 4.75 (dd, *J* = 8.3, 4.1 Hz, 1H, CH), 4.05 – 3.81 (m, 2H, Pro), 3.77 (s, 3H, OCH_3), 2.43 – 1.85 (m, 4H, Pro). ^{13}C NMR (75 MHz, $CDCl_3$) δ (ppm): 173.46, 162.56 (d, *J* = 246.8 Hz), 162.35, 139.44, 132.10 (d, *J* = 3.3 Hz), 129.23, 129.09 (d, *J* = 8.4 Hz), 127.15, 125.00, 123.74, 122.72, 115.86 (d, *J* = 21.6 Hz), 109.08, 60.75, 52.85, 52.22, 48.63, 30.47, 23.85. HRMS (ESI) *m/z* calcd. for $C_{21}H_{21}FN_3O_3$ $[M+H]^+$: 382.1561; found: 382.1566.

(*S*)-Methyl 1-(1-pentyl-1*H*-indole-3-carbonyl)piperidine-2-carboxylate (16a). Yield: 79%. ^{1}H NMR (300 MHz, $CDCl_3$) δ (ppm): 7.77 (d, *J* = 7.0 Hz, 1H, Ar-H), 7.47 (s, 1H, Ar-H), 7.36 (d, *J* = 7.4 Hz, 1H, Ar-H), 7.30 – 7.16 (m, 2H, Ar-H), 5.50 – 5.33 (m, 1H, CH), 4.13 (t, *J* = 7.2 Hz, 2H, $CH_2CH_2CH_2CH_2CH_3$), 3.80 (s, 3H, OCH_3), 3.38 – 3.22 (m, 1H, Pip), 2.33 (d, *J* = 13.5 Hz, 1H, Pip), 1.97 – 1.73 (m, 4H, Pip, $CH_2CH_2CH_2CH_2CH_3$), 1.71 – 1.43 (m, 3H, Pip), 1.43 – 1.25 (m, 5H, $CH_2CH_2CH_2CH_2CH_3$, $CH_2CH_2CH_2CH_2CH_3$, Pip), 0.89 (t, *J* = 6.8 Hz, 3H, $CH_2CH_2CH_2CH_2CH_3$). ^{13}C NMR (75 MHz, $CDCl_3$) δ (ppm): 172.36, 168.01, 135.93, 130.53, 126.44, 122.38, 120.92, 120.84, 110.55, 110.04, 55.02, 52.32, 46.88, 44.96, 29.87, 29.23, 27.33, 25.81, 22.41, 21.53, 14.04. LRMS (ESI) *m/z* calcd. for $C_{21}H_{29}N_2O_3$ $[M+H]^+$: 357.2; found: 357.2.

(*R*)-Methyl 1-(1-pentyl-1*H*-indole-3-carbonyl)piperidine-2-carboxylate (16b). Yield: 75%. ^{1}H NMR (300 MHz, $CDCl_3$) δ (ppm): 7.77 (d, *J* = 7.8 Hz, 1H, Ar-H), 7.46 (s, 1H, Ar-H), 7.36 (d, *J* = 7.4 Hz, 1H, Ar-H), 7.31 – 7.15 (m, 2H, Ar-H), 5.48 – 5.29 (m, 1H, CH), 4.12 (t, *J* = 7.2 Hz, 2H, $CH_2CH_2CH_2CH_2CH_3$), 3.79 (s, 3H, OCH_3), 3.41 – 3.20 (m, 1H, Pip), 2.32 (d, *J* = 13.6 Hz, 1H, Pip), 1.95 – 1.72 (m, 4H, $CH_2CH_2CH_2CH_2CH_3$), 1.72 – 1.41 (m, 3H, Pip), 1.38 – 1.29 (m, 5H, $CH_2CH_2CH_2CH_2CH_3$, $CH_2CH_2CH_2CH_2CH_3$, Pip), 0.89 (t, *J* = 6.8 Hz, 3H, $CH_2CH_2CH_2CH_2CH_3$). LRMS (ESI) *m/z* calcd. for $C_{21}H_{29}N_2O_3$ $[M+H]^+$: 357.2; found: 357.2.

(*S*)-Methyl 1-(1-(5-fluoropentyl)-1*H*-indole-3-carbonyl)piperidine-2-carboxylate (16c). Yield: 83%. ^{1}H NMR (300 MHz, $CDCl_3$) δ (ppm): 7.76 (d, *J* = 7.6 Hz, 1H, Ar-H), 7.46 (s, 1H, Ar-H), 7.35 (d, *J* = 7.9 Hz, 1H, Ar-H), 7.29 – 7.17 (m, 2H, Ar-H), 5.50 – 5.26 (m, 1H, CH), 4.50 (t, *J* = 5.9 Hz, 1H, $CH_2CH_2CH_2CH_2CH_2F$), 4.34 (t, *J* = 5.9 Hz, 1H, $CH_2CH_2CH_2CH_2CH_2F$), 4.15 (t, *J* = 7.1 Hz, 2H, $CH_2CH_2CH_2CH_2CH_2F$), 3.78 (s, 3H, OCH_3), 3.39 – 3.18 (m, 1H, Pip), 2.32 (d, *J* = 13.4 Hz, 1H, Pip), 1.99 – 1.84 (m, 4H, $CH_2CH_2CH_2CH_2CH_2F$, $CH_2CH_2CH_2CH_2CH_2F$, Pip), 1.84 – 1.63 (m, 6H, $CH_2CH_2CH_2$ CH_2CH_2F, $CH_2CH_2CH_2CH_2CH_2F$, Pip) 1.55 – 1.41 (m, 2H, $CH_2CH_2CH_2CH_2CH_2F$). ^{13}C NMR (75 MHz, $CDCl_3$) δ (ppm): 172.34, 164.72, 135.88, 130.39, 126.46, 122.51, 121.03, 120.90, 110.80, 109.95, 83.80 (d, *J* = 165.0 Hz), 54.23, 52.34, 46.72, 44.85, 30.12 (d, *J* = 19.8 Hz), 29.86, 27.33, 25.81, 23.04 (d, *J* = 4.9 Hz), 21.52. LRMS (ESI) *m/z* calcd. for $C_{21}H_{28}FN_2O_3$ $[M+H]^+$: 375.2; found: 375.2.

(*R*)-Methyl 1-(1-(5-fluoropentyl)-1*H*-indole-3-carbonyl)piperidine-2-carboxylate (16d). Yield: 74%. ^{1}H NMR (300 MHz, $CDCl_3$) δ (ppm): 7.77 (d, *J* = 7.5 Hz, 1H, Ar-H), 7.46 (s, 1H, Ar-H), 7.35 (d, *J* = 7.7 Hz, 1H, Ar-H), 7.30 – 7.17 (m, 2H, Ar-H), 5.48 – 5.29 (m, 1H, CH), 4.50 (t, *J* = 5.9 Hz, 1H, $CH_2CH_2CH_2CH_2C\mathit{H_2}F$), 4.35 (t, *J* = 5.9 Hz, 1H, $CH_2CH_2CH_2CH_2C\mathit{H_2}F$), 4.15 (t, *J* = 7.1 Hz, 2H, $C\mathit{H_2}CH_2CH_2CH_2CH_2F$), 3.79 (s, 3H, OCH_3), 3.38 – 3.20 (m, 1H, Pip), 2.32 (d, *J* = 13.4 Hz, 1H, Pip), 1.99 – 1.85 (m, 4H, $CH_2CH_2CH_2C\mathit{H_2}CH_2F$, $CH_2C\mathit{H_2}CH_2CH_2CH_2F$, Pip), 1.85 – 1.59 (m, 6H, $CH_2CH_2CH_2C\mathit{H_2}$ CH_2F, $CH_2C\mathit{H_2}CH_2CH_2CH_2F$, Pip), 1.52 – 1.41 (m, 2H, $CH_2CH_2C\mathit{H_2}CH_2CH_2F$). LRMS (ESI) *m/z* calcd. for $C_{21}H_{28}FN_2O_3$ $[M+H]^+$: 375.2; found: 375.2.

(*S*)-Methyl 1-(1-(cyclohexylmethyl)-1*H*-indole-3-carbonyl)piperidine-2-carboxylate (16e). Yield: 86%. ^{1}H NMR (300 MHz, $CDCl_3$) δ (ppm): 7.78 (d, *J* = 7.6 Hz, 1H, Ar-H), 7.44 (s, 1H, Ar-H), 7.36 (d, *J* = 8.4 Hz, 1H, Ar-H), 7.32 – 7.13 (m, 2H, Ar-H), 5.49 – 5.27 (m, 1H, CH), 4.31 – 4.07 (m, 1H, Pip), 3.96 (d, *J* = 7.3 Hz, 2H, $C\mathit{H_2}$-Cy), 3.80 (s, 3H, OCH_3), 3.42 – 3.20 (m, 1H, Pip), 2.33 (d, *J* = 13.7 Hz, 1H, C*H*-Cy), 1.97 – 1.59 (m, 8H, Pip, Cy), 1.59 – 1.35 (m, 1H, Pip, Cy), 1.32 – 1.10 (m, 4H, Pip, Cy), 1.10 – 0.89 (m, 3H, Pip, Cy). LRMS (ESI) *m/z* calcd. for $C_{23}H_{31}N_2O_3$ $[M+H]^+$: 383.2; found: 383.2.

(*R*)-Methyl 1-(1-(cyclohexylmethyl)-1*H*-indole-3-carbonyl)piperidine-2-carboxylate (16f). Yield: 74%. ^{1}H NMR (300 MHz, $CDCl_3$) δ (ppm): 7.75 (d, *J* = 7.8 Hz, 1H, Ar-H), 7.41 (s, 1H, Ar-H), 7.34 (d, *J* = 7.8 Hz, 1H, Ar-H), 7.28 – 7.13 (m, 2H, Ar-H), 5.52 – 5.21 (m, 1H, CH), 4.26 – 4.02 (m, 1H, Pip), 3.93 (d, *J* = 6.9 Hz, 2H, $C\mathit{H_2}$-Cy), 3.77 (s, 3H, OCH_3), 3.38 – 3.17 (m, 1H, Pip), 2.30 (d, *J* = 13.6 Hz, 1H, C*H*-Cy), 1.93 – 1.55 (m, 9H, Pip, Cy), 1.55 – 1.32 (m, 1H, Pip, Cy), 1.28 – 1.10 (m, 4H, Pip, Cy), 1.08 – 0.88 (m, 2H, Pip, Cy). ^{13}C NMR (75 MHz, $CDCl_3$) δ (ppm): 172.33, 167.99, 136.24, 131.18, 126.35, 122.30, 120.84, 120.76, 110.32, 110.26, 64.75, 53.36, 52.28, 38.72, 32.41, 31.16, 27.30, 26.34, 25.77, 24.04, 21.49. HRMS (ESI) *m/z* calcd. for $C_{23}H_{31}N_2O_3$ $[M+H]^+$: 383.2329; found: 383.2323.

(*S*)-Methyl 1-(1-(4-fluorobenzyl)-1*H*-indole-3-carbonyl)piperidine-2-carboxylate (16g). Yield: 72%. ^{1}H NMR (300 MHz, $CDCl_3$) δ (ppm): 7.80 (dd, *J* = 6.3, 2.8 Hz, 1H, Ar-H), 7.48 (s, 1H, Ar-H), 7.32 – 7.20 (m, 3H, Ar-H), 7.19 – 7.10 (m, 2H, Ar-H), 7.06 – 6.97 (m, 2H, Ar-H), 5.30 (s, 2H, CH_2-Ph), 4.24 – 4.07 (m, 1H, CH), 3.79 (s, 3H, OCH_3), 2.34 (d, *J* = 13.4 Hz, 1H, Pip), 1.91 – 1.73 (m, 2H, Pip), 1.72 – 1.32 (m, 5H, Pip). LRMS (ESI) *m/z* calcd. for $C_{23}H_{24}FN_2O_3$ $[M+H]^+$: 395.2; found: 395.2.

(*R*)-Methyl 1-(1-(4-fluorobenzyl)-1*H*-indole-3-carbonyl)piperidine-2-carboxylate (16h). Yield: 78%. ^{1}H NMR (300 MHz, $CDCl_3$) δ (ppm): 7.78 (dd, *J* = 6.2, 3.1 Hz, 1H, Ar-H), 7.45 (s, 1H, Ar-H), 7.30 – 7.16 (m, 3H, Ar-H), 7.16 – 7.06 (m, 2H, Ar-H), 7.04 – 6.90 (m, 2H, Ar-H), 5.26 (s, 2H, CH_2-Ph), 4.31 – 3.92 (m, 1H, CH), 3.77 (s, 3H, OCH_3), 2.31 (d, *J* = 13.5 Hz, 1H, Pip), 1.87 – 1.70 (m, 2H, Pip), 1.69 – 1.31 (m, 5H, Pip). ^{13}C NMR (75 MHz, $CDCl_3$) δ (ppm): 172.23, 167.70, 162.56 (d, *J* = 246.8 Hz), 136.02, 132.20 (d, *J* = 3.1 Hz), 130.55, 128.95 (d, *J* = 8.3 Hz), 126.62, 122.82, 121.31, 120.92, 115.98 (d, *J* = 21.8 Hz), 111.41, 110.26, 54.43, 52.32, 49.94, 45.17, 27.28, 25.76, 21.46. HRMS (ESI) *m/z* calcd. for $C_{23}H_{24}FN_2O_3$ $[M+H]^+$: 395.1765; found: 395.1773.

(*S*)-Methyl 1-(1-pentyl-1*H*-indazole-3-carbonyl)piperidine-2-carboxylate (16i). Yield: 81%. ^{1}H NMR (300 MHz, $CDCl_3$) δ (ppm): 8.20 (d, *J* = 7.9 Hz, 1H, Ar-H), 7.46 – 7.33 (m, 2H, Ar-H), 7.29 – 7.17 (m, 1H, Ar-H), 5.66 – 5.55 (m, 1H, CH), 4.39 (t, *J* = 7.2 Hz, 2H, C$H_2$$CH_2CH_2CH_2CH_3$), 3.78 (s, 3H, OCH_3), 3.41 – 3.23 (m, 1H, Pip), 2.34 (t, *J* = 16.5 Hz, 1H, Pip), 2.00 – 1.85 (m, 3H, Pip, CH_2C$H_2$$CH_2CH_2CH_3$), 1.83 – 1.73 (m, 3H, Pip), 1.70 – 1.42 (m, 2H, Pip), 1.39 – 1.20 (m, 4H, CH_2CH_2C$H_2$$CH_2CH_3$, $CH_2CH_2CH_2$C$H_2$$CH_3$), 0.87 (t, *J* = 6.8 Hz, 3H, $CH_2CH_2CH_2CH_2$CH_3). ^{13}C NMR (75 MHz, $CDCl_3$) δ (ppm): 172.00, 164.37, 138.99, 126.64, 123.10, 122.60, 122.23, 122.07, 109.15, 57.58, 52.59, 49.32, 42.73, 29.52, 29.33, 29.03, 27.34, 25.32, 21.89, 14.01. HRMS (ESI) *m/z* calcd. for $C_{20}H_{28}N_3O_3$ $[M+H]^+$: 358.2125; found: 358.2128.

(*R*)-Methyl 1-(1-pentyl-1*H*-indazole-3-carbonyl)piperidine-2-carboxylate (16j). Yield: 77%. ^{1}H NMR (300 MHz, $CDCl_3$) δ (ppm): 8.20 (d, *J* = 8.2 Hz, 1H, Ar-H), 7.47 – 7.34 (m, 2H, Ar-H), 7.33 – 7.15 (m, 1H, Ar-H), 5.68 – 5.54 (m, 1H, CH), 4.39 (t, *J* = 7.1 Hz, 2H, $CH_2CH_2CH_2CH_2CH_3$), 3.78 (s, 3H, OCH_3), 3.41 – 3.22 (m, 1H, Pip), 2.33 (t, *J* = 16.6 Hz, 1H, Pip), 2.02 – 1.85 (m, 3H, Pip, $CH_2CH_2CH_2CH_2CH_3$), 1.85 – 1.72 (m, 3H, Pip), 1.71 – 1.53 (m, 2H, Pip), 1.52 – 1.17 (m, 4H, $CH_2CH_2CH_2CH_2CH_3$, $CH_2CH_2CH_2CH_2CH_3$), 0.86 (t, *J* = 6.8 Hz, 3H, $CH_2CH_2CH_2CH_2CH_3$). ^{13}C NMR (75 MHz, $CDCl_3$) δ (ppm): 172.30, 164.35, 138.96, 126.65, 123.06, 122.56, 122.20, 122.04, 109.09, 57.55, 52.56, 49.28, 42.69, 29.48, 29.30, 29.00, 27.30, 25.28, 21.85, 13.98. HRMS (ESI) *m/z* calcd. for $C_{20}H_{28}N_3O_3$ $[M+H]^+$: 358.2125; found: 358.2124.

(*S*)-Methyl 1-(1-(5-fluoropentyl)-1*H*-indazole-3-carbonyl)piperidine-2-carboxylate (16k). Yield: 79%. ^{1}H NMR (300 MHz, $CDCl_3$) δ (ppm): 8.18 (d, *J* = 8.2 Hz, 1H, Ar-H), 7.43 – 7.31 (m, 2H, Ar-H), 7.26 – 7.15 (m, 1H, Ar-H), 5.64 – 5.51 (m, 1H, CH), 4.45 (t, *J* = 5.9 Hz, 1H, $CH_2CH_2CH_2CH_2CH_2F$), 4.29 (t, *J* = 5.9 Hz, 1H, $CH_2CH_2CH_2CH_2CH_2F$), 4.07 (t, *J* = 6.9 Hz, 2H, $CH_2CH_2CH_2CH_2CH_2F$), 3.76 (s, 3H, OCH_3), 3.38 – 3.23 (m, 1H, Pip), 2.41 – 2.20 (m, 1H, Pip), 1.99 – 1.87 (m, 2H, $CH_2CH_2CH_2CH_2CH_2F$, $CH_2CH_2CH_2CH_2CH_2F$, Pip), 1.87 – 1.53 (m, 6H, $CH_2CH_2CH_2CH_2CH_2F$, $CH_2CH_2CH_2CH_2CH_2F$, Pip), 1.51 – 1.33 (m, 4H, $CH_2CH_2CH_2CH_2CH_2F$, Pip). LRMS (ESI) *m/z* calcd. for $C_{20}H_{27}FN_3O_3$ $[M+H]^+$: 376.2; found: 376.2.

(*R*)-Methyl 1-(1-(5-fluoropentyl)-1*H*-indazole-3-carbonyl)piperidine-2-carboxylate (16l). Yield: 80%. ^{1}H NMR (300 MHz, $CDCl_3$) δ (ppm): 8.19 (d, *J* = 8.1 Hz, 1H, Ar-H), 7.46 – 7.33 (m, 2H, Ar-H), 7.29 – 7.15 (m, 1H, Ar-H), 5.64 – 5.53 (m, 1H, CH), 4.46 (t, *J* = 6.0 Hz, 1H, $CH_2CH_2CH_2CH_2CH_2F$), 4.31 (t, *J* = 6.0 Hz, 1H, $CH_2CH_2CH_2CH_2CH_2F$), 4.11 (t, *J* = 7.0 Hz, 2H, $CH_2CH_2CH_2CH_2CH_2F$) 3.77 (s, 3H, OCH_3), 3.40 – 3.23 (m, 1H, Pip), 2.44 – 2.23 (m, 1H, Pip), 2.05 – 1.89 (m, 2H, $CH_2CH_2CH_2CH_2CH_2F$, $CH_2CH_2CH_2CH_2CH_2F$, Pip), 1.89 – 1.57 (m, 6H, $CH_2CH_2CH_2CH_2CH_2F$, $CH_2CH_2CH_2CH_2CH_2F$, Pip), 1.56 – 1.31 (m, 4H, CH_2 $CH_2CH_2CH_2CH_2F$, Pip). ^{13}C NMR (75 MHz, $CDCl_3$) δ (ppm): 172.29, 164.18, 139.07, 126.81, 124.33, 123.12, 122.60, 122.22, 109.00, 83.79 (d, *J* = 165.1 Hz), 57.57, 52.58, 49.04, 42.71, 30.00 (d, *J* = 19.7 Hz), 29.30, 27.32, 25.28, 22.76 (d, *J* = 5.1 Hz), 21.40. HRMS (ESI) *m/z* calcd. for $C_{20}H_{27}FN_3O_3$ $[M+H]^+$: 376.2031; found: 376.2029.

(*S*)-Methyl 1-(1-(cyclohexylmethyl)-1*H*-indazole-3-carbonyl)piperidine-2-carboxylate (16m). Yield: 85%. ^{1}H NMR (300 MHz, $CDCl_3$) δ (ppm): 8.20 (d, *J* = 8.2 Hz, 1H, Ar-H), 7.43 – 7.33 (m, 2H, Ar-H), 7.28 – 7.16 (m, 1H, Ar-H), 5.64 – 5.54 (m, 1H, CH), 4.21 (d, *J* = 7.1 Hz, 2H, CH_2-Cy), 3.78 (s, 3H, OCH_3), 3.39 – 3.25 (m, 2H, Pip), 2.43 – 2.24 (m, 1H, C*H*-Cy), 2.11 – 1.34 (m, 12H, Pip, Cy), 1.30 – 0.89 (m, 4H, Pip, Cy). LRMS (ESI) *m/z* calcd. for $C_{22}H_{30}N_3O_3$ $[M+H]^+$: 384.2; found: 384.2.

(*R*)-Methyl 1-(1-(cyclohexylmethyl)-1*H*-indazole-3-carbonyl)piperidine-2-carboxylate (16n). Yield: 80%. ^{1}H NMR (500 MHz, DMSO-d_6) δ (ppm): 7.99 (d, *J* = 8.2 Hz, 1H, Ar-H), 7.68 (dd, *J* = 8.5, 1.0 Hz, 1H, Ar-H), 7.43 (ddd, *J* = 8.3, 6.8, 1.1 Hz, 1H, Ar-H), 7.23 (ddd, *J* = 7.9, 6.8, 0.8 Hz, 1H, Ar-H), 4.60 – 4.53 (m, 1H, CH), 4.30 (d, *J* = 6.9 Hz, 2H, CH_2-Cy), 3.71 (s, 3H, OCH_3), 2.24 – 2.17 (m, 1H, Pip), 2.03 – 1.93 (m, 1H, C*H*-Cy), 1.85 – 1.76 (m, 1H, Pip), 1.76 – 1.36 (m, 12H, Pip, Cy), 1.24 – 1.01 (m, 4H, Pip, Cy). ^{13}C NMR (75 MHz, $CDCl_3$) δ (ppm): 172.35, 164.39, 139.22, 126.60, 124.49, 122.80, 122.10, 109.34, 56.56, 52.85, 52.35, 42.73, 38.76, 31.02, 29.82, 27.33, 26.35, 25.73, 24.92, 21.47. HRMS (ESI) *m/z* calcd. for $C_{22}H_{30}N_3O_3$ $[M+H]^+$: 384.2282; found: 384.2288.

(*S*)-Methyl 1-(1-(4-fluorobenzyl)-1*H*-indazole-3-carbonyl)piperidine-2-carboxylate (16o). Yield: 92%. ^{1}H NMR (300 MHz, $CDCl_3$) δ (ppm): 8.22 (d, *J* = 8.2 Hz, 1H, Ar-H), 7.41 – 7.30 (m, 2H, Ar-H), 7.29 – 7.11 (m, 3H, Ar-H), 6.98 (t, *J* = 8.3 Hz, 2H, Ar-H), 5.58 (s, 2H, CH_2-Ph), 4.84 – 4.63 (m, 1H, CH), 3.79 (s, 3H, OCH_3), 3.42 – 3.25 (m, 2H, Pip), 2.45 – 2.24 (m, 1H, Pip), 1.93 – 1.35 (m, 5H, Pip). LRMS (ESI) *m/z* calcd. for $C_{22}H_{23}FN_3O_3$ $[M+H]^+$: 396.2; found: 396.2.

(*R*)-Methyl 1-(1-(4-fluorobenzyl)-1*H*-indazole-3-carbonyl)piperidine-2-carboxylate (16p). Yield: 87%. ^{1}H NMR (300 MHz, $CDCl_3$) δ (ppm): 8.22 (d, *J* = 8.1 Hz, 1H, Ar-H), 7.41 – 7.31 (m, 2H, Ar-H), 7.29 – 7.12 (m, 3H, Ar-H), 6.98 (t, *J* = 8.4 Hz, 2H, Ar-H), 5.58 (s, 2H, CH_2-Ph), 4.85 – 4.63 (m, 1H, CH), 3.79 (s, 3H, OCH_3), 3.43 – 3.23 (m, 2H, Pip), 2.46 – 2.26 (m, 1H, Pip), 1.93 – 1.34 (m, 5H, Pip). ^{13}C NMR (75 MHz, $CDCl_3$) δ (ppm): 172.24, 164.21, 162.57 (d, *J* = 246.5 Hz), 139.38, 132.00 (d, *J* = 3.2 Hz), 129.26, 129.15 (d, *J* = 8.2 Hz), 127.16, 124.73, 122.96, 122.48, 115.83 (d, *J* = 21.6 Hz), 109.27, 57.59, 52.83, 52.39, 42.77, 27.33, 25.28, 21.39. HRMS (ESI) *m/z* calcd. for $C_{22}H_{23}FN_3O_3$ $[M+H]^+$: 396.1718; found: 396.1719.

(*S*)-1-(1-pentyl-1*H*-indole-3-carbonyl)pyrrolidine-2-carboxamide (17a). Yield: 77%. ^{1}H NMR (300 MHz, $CDCl_3$) δ (ppm): 8.12 (d, *J* = 7.7 Hz, 1H, Ar-H), 7.45 (s, 1H, Ar-H), 7.36 – 7.12 (m, 3H, Ar-H), 5.88 (s, 2H, NH_2), 4.96 – 4.67 (m, 1H, CH), 4.19 – 3.92 (m, 2H, Pro), 3.79 (t, *J* = 6.6 Hz, 2H, $CH_2CH_2CH_2CH_2CH_3$), 2.39 – 2.21 (m, 1H, Pro), 2.16 – 1.96 (m, 2H, Pro), 1.96 – 1.66 (m, 3H, Pro, $CH_2CH_2CH_2CH_2CH_3$), 1.40 – 1.19 (m, 4H, CH_2CH_2 $CH_2CH_2CH_3$, $CH_2CH_2CH_2CH_2CH_3$), 0.85 (t, *J* = 6.8 Hz, 3H, $CH_2CH_2CH_2CH_2CH_3$). LRMS (ESI) *m/z* calcd. for $C_{19}H_{26}N_3O_2$ $[M+H]^+$: 328.2; found: 328.2.

(*R*)-1-(1-pentyl-1*H*-indole-3-carbonyl)pyrrolidine-2-carboxamide (17b). Yield: 73%. ^{1}H NMR (300 MHz, $CDCl_3$) δ (ppm): 8.14 (d, *J* = 7.8 Hz, 1H, Ar-H), 7.46 (s, 1H, Ar-H), 7.38 – 7.12 (m, 3H, Ar-H), 5.67 (s, 2H, NH_2), 4.97 – 4.76 (m, 1H, CH), 4.20 – 3.98 (m, 2H, Pro), 3.81 (t, *J* = 6.7 Hz, 2H, $CH_2CH_2CH_2CH_2CH_3$), 2.46 – 2.29 (m, 1H, Pro), 2.17 – 1.98 (m, 2H, Pro), 1.98 – 1.73 (m, 3H, Pro, $CH_2CH_2CH_2CH_2CH_3$), 1.42 – 1.18 (m, 4H, CH_2CH_2 $CH_2CH_2CH_3$, $CH_2CH_2CH_2CH_2CH_3$), 0.87 (t, *J* = 6.7 Hz, 3H, $CH_2CH_2CH_2CH_2CH_3$). ^{13}C NMR (75 MHz, $CDCl_3$) δ (ppm): 174.62, 166.84, 135.94, 130.41, 127.80, 122.92, 122.21, 121.45, 109.80, 60.21, 54.61, 49.65, 47.01, 29.81, 29.11, 27.35, 25.47, 22.35, 14.00. LRMS (ESI) *m/z* calcd. for $C_{19}H_{26}N_3O_2$ $[M+H]^+$: 328.2; found: 328.2.

(*S*)-1-(1-(5-fluoropentyl)-1*H*-indole-3-carbonyl)pyrrolidine-2-carboxamide (17c). Yield: 75%. ^{1}H NMR (300 MHz, $CDCl_3$) δ (ppm): 8.12 (d, *J* = 7.8 Hz, 1H, Ar-H), 7.45 (s, 1H, Ar-H), 7.36 – 7.11 (m, 3H, Ar-H), 5.88 (s, 2H, NH_2), 4.95 – 4.66 (m, 1H, CH), 4.45 (t, *J* = 5.9 Hz, 1H, $CH_2CH_2CH_2CH_2CH_2F$), 4.29 (t, *J* = 5.9 Hz, 1H, $CH_2CH_2CH_2CH_2CH_2F$), 4.19 – 3.96 (m, 2H, Pro), 3.78 (t, *J* = 6.4 Hz, 2H, $CH_2CH_2CH_2CH_2CH_2F$), 2.41 – 2.21 (m, 1H, Pro), 2.18 – 1.53 (m, 7H, Pro, $CH_2CH_2CH_2CH_2CH_2F$, $CH_2CH_2CH_2CH_2CH_2F$), 1.50 – 1.21 (m, 2H, $CH_2CH_2CH_2CH_2CH_2F$). LRMS (ESI) *m/z* calcd. for $C_{19}H_{25}FN_3O_2$ $[M+H]^+$: 346.2; found: 346.2.

(*R*)-1-(1-(5-fluoropentyl)-1*H*-indole-3-carbonyl)pyrrolidine-2-carboxamide (17d). Yield: 79%. ^{1}H NMR (300 MHz, $CDCl_3$) δ (ppm): 8.14 (d, *J* = 7.7 Hz, 1H, Ar-H), 7.46 (s, 1H, Ar-H), 7.37 – 7.04 (m, 3H, Ar-H), 5.79 (s, 2H, NH_2), 5.02 – 4.65 (m, 1H, CH), 4.47 (t, *J* = 5.9 Hz, 1H, $CH_2CH_2CH_2CH_2C\mathit{H_2}F$), 4.32 (t, *J* = 5.9 Hz, 1H, $CH_2CH_2CH_2CH_2C\mathit{H_2}F$), 4.22 – 3.95 (m, 2H, Pro), 3.80 (t, *J* = 6.5 Hz, 2H, $C\mathit{H_2}CH_2CH_2CH_2CH_2F$), 2.45 – 2.19 (m, 1H, Pro), 2.17 – 1.53 (m, 7H, Pro, $CH_2CH_2CH_2C\mathit{H_2}CH_2F$, $CH_2C\mathit{H_2}CH_2CH_2CH_2F$), 1.51 – 1.20 (m, 2H, $CH_2CH_2C\mathit{H_2}CH_2CH_2F$). LRMS (ESI) *m/z* calcd. for $C_{19}H_{25}FN_3O_2$ $[M+H]^+$: 346.2; found: 346.2.

(*S*)-1-(1-(cyclohexylmethyl)-1*H*-indole-3-carbonyl)pyrrolidine-2-carboxamide (17e). Yield: 73%. ^{1}H NMR (300 MHz, $CDCl_3$) δ (ppm): 8.16 (dd, *J* = 7.1, 1.9 Hz, 1H, Ar-H), 7.40 (s, 1H, Ar-H), 7.36 – 7.15 (m, 3H, Ar-H), 5.78 (s, 2H, NH_2), 5.01 – 4.71 (m, 1H, CH), 3.92 (d, *J* = 7.2 Hz, 2H, $C\mathit{H_2}$-Cy), 3.85 – 3.70 (m, 2H, Pro), 2.49 – 2.28 (m, 1H, C*H*-Cy), 2.22 – 1.77 (m, 4H, Cy, Pro), 1.77 – 1.44 (m, 4H, Cy, Pro), 1.36 – 1.07 (m, 4H, Cy, Pro), 1.06 – 0.92 (m, 2H, Cy). ^{13}C NMR (75 MHz, $CDCl_3$) δ (ppm): 174.60, 166.79, 136.25, 131.05, 127.75, 122.86, 122.21, 121.40, 110.03, 109.66, 60.30, 53.49, 49.60, 38.66, 31.08, 27.44, 26.27, 25.82, 25.72. LRMS (ESI) *m/z* calcd. for $C_{21}H_{28}N_3O_2$ $[M+H]^+$: 354.2; found: 354.2.

(*R*)-1-(1-(cyclohexylmethyl)-1*H*-indole-3-carbonyl)pyrrolidine-2-carboxamide (17f). Yield: 80%. ^{1}H NMR (300 MHz, $CDCl_3$) δ (ppm): 8.15 (dd, *J* = 7.3, 1.8 Hz, 1H, Ar-H), 7.42 (s, 1H, Ar-H), 7.37 – 7.16 (m, 3H, Ar-H), 5.68 (s, 2H, NH_2), 5.02 – 4.73 (m, 1H, CH), 3.93 (d, *J* = 7.3 Hz, 2H, $C\mathit{H_2}$-Cy), 3.87 – 3.74 (m, 2H, Pro), 2.51 – 2.30 (m, 1H, C*H*-Cy), 2.19 – 1.76 (m, 4H, Cy, Pro), 1.76 – 1.51 (m, 4H, Cy, Pro), 1.24 – 1.10 (m, 4H, Cy, Pro), 1.07 – 0.91 (m, 2H, Cy). LRMS (ESI) *m/z* calcd. for $C_{21}H_{28}N_3O_2$ $[M+H]^+$: 354.2; found: 354.2.

(*S*)-1-(1-(4-fluorobenzyl)-1*H*-indole-3-carbonyl)pyrrolidine-2-carboxamide (17g). Yield: 72%. ^{1}H NMR (300 MHz, $CDCl_3$) δ (ppm): 8.22 – 8.10 (m, 1H, Ar-H), 7.48 (s, 1H, Ar-H), 7.27 – 7.13 (m, 3H, Ar-H), 7.12 – 7.02 (m, 2H, Ar-H), 7.02 – 6.91 (m, 2H, Ar-H), 5.86 (s, 2H, NH_2), 5.24 (s, 2H, $C\mathit{H_2}$-Ph), 4.95 – 4.71 (m, 1H, CH), 3.86 – 3.68 (m, 2H, Pro), 2.41 – 2.24 (m, 1H, Pro), 2.24 – 1.99 (m, 2H, Pro) 1.97 – 1.79 (m, 1H, Pro). LRMS (ESI) *m/z* calcd. for $C_{21}H_{21}FN_3O_2$ $[M+H]^+$: 366.2; found: 366.2.

(*R*)-1-(1-(4-fluorobenzyl)-1*H*-indole-3-carbonyl)pyrrolidine-2-carboxamide (17h). Yield: 76%. ^1H NMR (300 MHz, $CDCl_3$) δ (ppm): 8.23 – 8.09 (m, 1H, Ar-H), 7.48 (s, 1H, Ar-H), 7.25 – 7.13 (m, 3H, Ar-H), 7.11 – 7.00 (m, 2H, Ar-H), 7.00 – 6.89 (m, 2H, Ar-H), 5.96 (s, 2H, NH_2), 5.22 (s, 2H, CH_2-Ph), 4.92 – 4.67 (m, 1H, CH), 3.90 – 3.63 (m, 2H, Pro), 2.41 – 2.18 (m, 1H, Pro), 2.17 – 1.96 (m, 2H, Pro), 1.96 – 1.73 (m, 1H, Pro). LRMS (ESI) *m/z* calcd. for $C_{21}H_{21}FN_3O_2$ $[M+H]^+$: 366.2; found: 366.2.

(*S*)-1-(1-pentyl-1*H*-indazole-3-carbonyl)pyrrolidine-2-carboxamide (17i). Yield: 80%. ^1H NMR (300 MHz, $CDCl_3$) δ (ppm): 8.32 (d, J = 8.2 Hz, 1H, Ar-H), 7.38 (s, 2H, Ar-H), 7.29 – 7.19 (m, 1H, Ar-H), 5.86 (s, 2H, NH_2), 5.02 – 4.77 (m, 1H, CH), 4.37 (t, J = 7.0 Hz, 2H, $CH_2CH_2CH_2CH_2CH_3$), 4.26 – 3.73 (m, 2H, Pro), 2.46 – 2.29 (m, 1H, Pro), 2.24 – 1.77 (m, 5H, Pro, $CH_2CH_2CH_2CH_2CH_3$), 1.47 – 1.12 (m, 4H, $CH_2CH_2CH_2CH_2CH_3$, CH_2CH_2 $CH_2CH_2CH_3$), 0.86 (t, J = 6.9 Hz, 3H, $CH_2CH_2CH_2CH_2CH_3$). LRMS (ESI) *m/z* calcd. for $C_{18}H_{25}N_4O_2$ $[M+H]^+$: 329.2; found: 329.2.

(*R*)-1-(1-pentyl-1*H*-indazole-3-carbonyl)pyrrolidine-2-carboxamide (17j). Yield: 72%. ^1H NMR (300 MHz, $CDCl_3$) δ (ppm): 8.31 (d, J = 8.1 Hz, 1H, Ar-H), 7.39 (s, 2H, Ar-H), 7.30 – 7.19 (m, 1H, Ar-H), 5.84 (s, 2H, NH_2), 4.98 – 4.82 (m, 1H, CH), 4.37 (t, J = 7.1 Hz, 2H, $CH_2CH_2CH_2CH_2CH_3$), 4.26 – 3.75 (m, 2H, Pro), 2.44 – 2.30 (m, 1H, Pro), 2.26 – 1.81 (m, 5H, Pro, $CH_2CH_2CH_2CH_2CH_3$), 1.44 – 1.14 (m, 4H, $CH_2CH_2CH_2CH_2CH_3$, CH_2CH_2 $CH_2CH_2CH_3$), 0.86 (t, J = 6.7 Hz, 3H, $CH_2CH_2CH_2CH_2CH_3$). LRMS (ESI) *m/z* calcd. for $C_{18}H_{25}N_4O_2$ $[M+H]^+$: 329.2; found: 329.2.

(*S*)-1-(1-(5-fluoropentyl)-1*H*-indazole-3-carbonyl)pyrrolidine-2-carboxamide (17k). Yield: 77%. ^1H NMR (300 MHz, $CDCl_3$) δ (ppm): 8.32 (d, J = 9.2 Hz, 1H, Ar-H), 7.39 (s, 2H, Ar-H), 7.30 – 7.17 (m, 1H, Ar-H), 5.68 (s, 2H, NH_2), 5.03 – 4.77 (m, 1H, CH), 4.48 (t, J = 5.9 Hz, 1H, $CH_2CH_2CH_2CH_2CH_2F$), 4.40 (t, J = 7.4 Hz, 2H, $CH_2CH_2CH_2$ CH_2CH_2F), 4.32 (t, J = 5.9 Hz, 1H, $CH_2CH_2CH_2CH_2CH_2F$), 4.27 – 3.77 (m, 2H, Pro), 2.50 – 2.28 (m, 1H, Pro), 2.11 – 1.85 (m, 5H, Pro, $CH_2CH_2CH_2CH_2CH_2F$, $CH_2CH_2CH_2CH_2CH_2F$), 1.83 – 1.55 (m, 2H, $CH_2CH_2CH_2CH_2CH_2F$, $CH_2CH_2CH_2CH_2CH_2F$), 1.53 – 1.29 (m, 2H, $CH_2CH_2CH_2CH_2CH_2F$). LRMS (ESI) *m/z* calcd. for $C_{18}H_{24}FN_4O_2$ $[M+H]^+$: 347.2; found: 347.2.

(*R*)-1-(1-(5-fluoropentyl)-1*H*-indazole-3-carbonyl)pyrrolidine-2-carboxamide (17l). Yield: 79%. ^{1}H NMR (300 MHz, $CDCl_3$) δ (ppm): 8.31 (d, *J* = 8.5 Hz, 1H, Ar-H), 7.39 (s, 2H, Ar-H), 7.31 – 7.20 (m, 1H, Ar-H), 5.80 (s, 2H, NH_2), 4.92 – 4.84 (m, 1H, CH), 4.47 (t, *J* = 6.0 Hz, 1H, $CH_2CH_2CH_2CH_2C\mathit{H_2}F$), 4.40 (t, *J* = 7.0 Hz, 2H, $C\mathit{H_2}CH_2$ $CH_2CH_2CH_2F$), 4.32 (t, *J* = 6.0 Hz, 1H, $CH_2CH_2CH_2CH_2C\mathit{H_2}F$), 4.24 – 3.78 (m, 2H, Pro), 2.44 – 2.31 (m, 1H, Pro), 2.29 – 1.88 (m, 5H, Pro, $CH_2CH_2CH_2C\mathit{H_2}CH_2F$, $CH_2C\mathit{H_2}CH_2CH_2CH_2F$), 1.83 – 1.57 (m, 2H, $CH_2CH_2CH_2C\mathit{H_2}CH_2F$, $CH_2C\mathit{H_2}CH_2CH_2CH_2F$), 1.54 – 1.29 (m, 2H, CH_2 $CH_2C\mathit{H_2}CH_2CH_2F$). ^{13}C NMR (75 MHz, $CDCl_3$) δ (ppm): 175.11, 163.80, 139.09, 126.92, 124.77, 123.26, 122.71, 109.08, 83.86 (d, *J* = 164.8 Hz), 61.98, 49.57, 47.85, 32.09, 29.99 (d, *J* = 19.8 Hz), 29.14, 26.43, 22.79 (d, *J* = 4.9 Hz), 22.10. LRMS (ESI) *m/z* calcd. for $C_{18}H_{24}FN_4O_2$ $[M+H]^+$: 347.2; found: 347.2.

(*S*)-1-(1-(cyclohexylmethyl)-1*H*-indazole-3-carbonyl)pyrrolidine-2-carboxamide (17m). Yield: 73%. ^{1}H NMR (300 MHz, $CDCl_3$) δ (ppm): 8.33 (d, *J* = 8.1 Hz, 1H, Ar-H), 7.40 (s, 2H, Ar-H), 7.30 – 7.18 (m, 1H, Ar-H), 5.76 (s, 2H, NH_2), 5.05 – 4.78 (m, 1H, CH), 4.32 – 4.13 (m, 2H, $C\mathit{H_2}$-Cy), 4.14 – 3.73 (m, 2H, Pro), 2.51 – 2.29 (m, 1H, C*H*-Cy), 2.21 – 1.89 (m, 4H, Cy, Pro), 1.80 – 1.46 (m, 4H, Cy, Pro), 1.34 – 0.91 (m, 6H, Cy, Pro). LRMS (ESI) *m/z* calcd. for $C_{20}H_{27}N_4O_2$ $[M+H]^+$: 355.2; found: 355.2.

(*R*)-1-(1-(cyclohexylmethyl)-1*H*-indazole-3-carbonyl)pyrrolidine-2-carboxamide (17n). Yield: 73%. ^{1}H NMR (300 MHz, $CDCl_3$) δ (ppm): 8.31 (d, *J* = 8.6 Hz, 1H, Ar-H), 7.39 (s, 2H, Ar-H), 7.30 – 7.11 (m, 1H, Ar-H), 5.74 (s, 2H, NH_2), 4.99 – 4.80 (m, 1H, CH), 4.30 – 4.13 (m, 2H, $C\mathit{H_2}$-Cy), 4.12 – 3.75 (m, 2H, Pro), 2.49 – 2.30 (m, 1H, C*H*-Cy), 2.26 – 1.88 (m, 4H, Cy, Pro), 1.84 – 1.44 (m, 4H, Cy, Pro), 1.36 – 0.87 (m, 6H, Cy, Pro). LRMS (ESI) *m/z* calcd. for $C_{20}H_{27}N_4O_2$ $[M+H]^+$: 355.2; found: 355.2.

(*S*)-1-(1-(4-fluorobenzyl)-1*H*-indazole-3-carbonyl)pyrrolidine-2-carboxamide (17o). Yield: 85%. ^{1}H NMR (300 MHz, $CDCl_3$) δ (ppm): 8.36 (d, J = 8.0 Hz, 1H, Ar-H), 7.45 – 7.22 (m, 5H, Ar-H), 7.07 – 6.93 (m, 2H, Ar-H), 5.59 (s, 2H, NH_2), 5.51 (s, 2H, CH_2-Ph), 5.03 – 4.82 (m, 1H, CH), 4.34 – 3.81 (m, 2H, Pro), 2.55 – 2.28 (m, 1H, Pro), 2.27 – 1.65 (m, 3H, Pro). ^{13}C NMR (75 MHz, $CDCl_3$) δ (ppm): 175.04, 163.60, 162.60 (d, J = 246.9 Hz), 140.01, 131.90 (d, J = 3.2 Hz), 129.57, 129.20 (d, J = 8.2 Hz), 127.25, 125.10, 123.37, 122.91, 115.89 (d, J = 21.7 Hz), 109.29, 61.96, 52.98, 48.91, 29.66, 23.89. LRMS (ESI) *m/z* calcd. for $C_{20}H_{20}FN_4O_2$ $[M+H]^+$: 367.2; found: 367.2.

(*R*)-1-(1-(4-fluorobenzyl)-1*H*-indazole-3-carbonyl)pyrrolidine-2-carboxamide (17p). Yield: 78%. ^{1}H NMR (300 MHz, $CDCl_3$) δ (ppm): 8.34 (d, J = 8.2 Hz, 1H, Ar-H), 7.44 – 7.33 (m, 2H, Ar-H), 7.33 – 7.06 (m, 3H, Ar-H), 7.06 – 6.91 (m, 2H, Ar-H), 5.58 (s, 2H, NH_2), 5.47 (s, 2H, CH_2-Ph), 4.96 – 4.86 (m, 1H, CH), 4.30 – 3.80 (m, 2H, Pro), 2.50 – 2.29 (m, 1H, Pro), 2.29 – 1.72 (m, 3H, Pro). LRMS (ESI) *m/z* calcd. for $C_{20}H_{20}FN_4O_2$ $[M+H]^+$: 367.2; found: 367.2.

(*S*)-1-(1-pentyl-1*H*-indole-3-carbonyl)piperidine-2-carboxamide (18a). Yield: 81%. ^{1}H NMR (300 MHz, $CDCl_3$) δ (ppm): 7.77 (d, J = 7.7 Hz, 1H, Ar-H), 7.43 (s, 1H, Ar-H), 7.36 (d, J = 8.0 Hz, 1H, Ar-H), 7.30 – 7.16 (m, 2H, Ar-H), 6.75 (s, 1H, NH_2), 5.56 (s, 1H, NH_2), 5.26 – 5.13 (m, 1H, CH), 4.28 – 4.15 (m, 1H, Pip), 4.10 (t, J = 7.2 Hz, 2H, $CH_2CH_2CH_2CH_2CH_3$), 3.17 – 3.00 (m, 1H, Pip), 2.35 (d, J = 13.0 Hz, 1H, Pip), 1.94 – 1.55 (m, 7H, Pip, $CH_2CH_2CH_2CH_2CH_3$), 1.43 – 1.22 (m, 4H, $CH_2CH_2CH_2CH_2CH_3$, $CH_2CH_2CH_2CH_2CH_3$), 0.88 (t, J = 6.5 Hz, 3H, $CH_2CH_2CH_2CH_2CH_3$). ^{13}C NMR (75 MHz, $CDCl_3$) δ (ppm): 173.96, 168.48, 136.11, 130.74, 126.66, 122.69, 121.25, 120.76, 110.19, 109.67, 54.18, 46.96, 45.52, 29.84, 29.18, 26.04, 25.77, 22.38, 20.98, 14.02. LRMS (ESI) *m/z* calcd. for $C_{20}H_{28}N_3O_2$ $[M+H]^+$: 342.2; found: 342.2.

(*R*)-1-(1-pentyl-1*H*-indole-3-carbonyl)piperidine-2-carboxamide (18b). Yield: 83%. ^{1}H NMR (300 MHz, $CDCl_3$) δ (ppm): 7.78 (d, *J* = 7.7 Hz, 1H, Ar-H), 7.44 (s, 1H, Ar-H), 7.37 (d, *J* = 7.8 Hz, 1H, Ar-H), 7.31 – 7.17 (m, 2H, Ar-H), 6.78 (s, 1H, NH_2), 5.64 (s, 1H, NH_2), 5.28 – 5.16 (m, 1H, CH), 4.27 – 4.16 (m, 1H, Pip), 4.11 (t, *J* = 7.2 Hz, 2H, $CH_2CH_2CH_2$ CH_2CH_3), 3.17 – 3.03 (m, 1H, Pip), 2.36 (d, *J* = 13.1 Hz, 1H, Pip), 1.94 – 1.54 (m, 7H, Pip, $CH_2CH_2CH_2CH_2CH_3$), 1.42 – 1.20 (m, 4H, $CH_2CH_2CH_2CH_2CH_3$, $CH_2CH_2CH_2CH_2CH_3$), 0.89 (t, *J* = 6.6 Hz, 3H, $CH_2CH_2CH_2CH_2CH_3$). ^{13}C NMR (75 MHz, $CDCl_3$) δ (ppm): 173.96, 168.46, 136.10, 130.71, 126.66, 122.68, 121.23, 120.77, 110.17, 109.67, 54.24, 46.95, 45.49, 29.84, 29.18, 26.03, 25.79, 22.37, 20.98, 14.01. LRMS (ESI) *m/z* calcd. for $C_{20}H_{28}N_3O_2$ $[M+H]^+$: 342.2; found: 342.2.

(*S*)-1-(1-(5-fluoropentyl)-1*H*-indole-3-carbonyl)piperidine-2-carboxamide (18c). Yield: 89%. ^{1}H NMR (300 MHz, $CDCl_3$) δ (ppm): 7.79 (d, *J* = 7.7 Hz, 1H, Ar-H), 7.46 (s, 1H, Ar-H), 7.37 (d, *J* = 7.9 Hz, 1H, Ar-H), 7.32 – 7.19 (m, 2H, Ar-H), 6.71 (s, 1H, NH_2), 5.42 (s, 1H, NH_2), 5.28 – 5.18 (m, 1H, CH), 4.51 (t, *J* = 5.9 Hz, 1H, $CH_2CH_2CH_2CH_2CH_2F$), 4.35 (t, *J* = 5.9 Hz, 1H, $CH_2CH_2CH_2CH_2CH_2F$), 4.17 (t, *J* = 7.1 Hz, 2H, $CH_2CH_2CH_2CH_2CH_2F$), 3.18 – 3.02 (m, 1H, Pip), 2.37 (d, *J* = 13.7 Hz, 1H, Pip), 1.99 – 1.85 (m, 2H, $CH_2CH_2CH_2$ CH_2CH_2F), 1.83 – 1.56 (m, 8H, $CH_2CH_2CH_2CH_2CH_2F$, Pip), 1.54 – 1.42 (m, 2H, CH_2CH_2 $CH_2CH_2CH_2F$). LRMS (ESI) *m/z* calcd. for $C_{20}H_{27}FN_3O_2$ $[M+H]^+$: 360.2; found: 360.2.

(*R*)-1-(1-(5-fluoropentyl)-1*H*-indole-3-carbonyl)piperidine-2-carboxamide (18d). Yield: 72%. ^{1}H NMR (300 MHz, $CDCl_3$) δ (ppm): 7.79 (d, *J* = 7.7 Hz, 1H, Ar-H), 7.45 (s, 1H, Ar-H), 7.37 (d, *J* = 8.0 Hz, 1H, Ar-H), 7.33 – 7.19 (m, 2H, Ar-H), 6.74 (s, 1H, NH_2), 5.56 (s, 1H, NH_2), 5.27 – 5.17 (m, 1H, CH), 4.50 (t, *J* = 5.9 Hz, 1H, $CH_2CH_2CH_2CH_2CH_2F$), 4.35 (t, *J* = 5.9 Hz, 1H, $CH_2CH_2CH_2CH_2CH_2F$), 4.15 (t, *J* = 7.1 Hz, 2H, $CH_2CH_2CH_2CH_2CH_2F$), 3.19 – 3.03 (m, 1H, Pip), 2.36 (d, *J* = 13.0 Hz, 1H, Pip), 1.99 – 1.85 (m, 2H, CH_2CH_2 $CH_2CH_2CH_2F$), 1.83 – 1.60 (m, 8H, $CH_2CH_2CH_2CH_2CH_2F$, Pip), 1.54 – 1.41 (m, 2H, $CH_2CH_2CH_2CH_2CH_2F$). ^{13}C NMR (75 MHz, $CDCl_3$) δ (ppm): 173.91, 168.37, 136.04, 130.61, 126.68, 122.80, 121.32, 120.82, 110.10, 109.87, 83.76 (d, *J* = 165.0 Hz), 54.04, 46.78, 45.47, 30.06 (d, *J* = 19.9 Hz), 29.81, 26.01, 25.81, 23.02 (d, *J* = 4.8 Hz), 20.96. LRMS (ESI) *m/z* calcd. for $C_{20}H_{27}FN_3O_2$ $[M+H]^+$: 360.2; found: 360.2.

(*S*)-1-(1-(cyclohexylmethyl)-1*H*-indole-3-carbonyl)piperidine-2-carboxamide (18e). Yield: 84%. ^{1}H NMR (300 MHz, $CDCl_3$) δ (ppm): 7.81 (dd, *J* = 8.0, 1.2 Hz, 1H, Ar-H), 7.42 (s, 1H, Ar-H), 7.40 – 7.34 (m, 1H, Ar-H), 7.32 – 7.19 (m, 2H, Ar-H), 6.80 (s, 1H, NH_2), 5.70 (s, 1H, NH_2), 5.28 – 5.18 (m, 1H, CH), 4.31 – 4.15 (m, 1H, Pip), 3.96 (d, *J* = 7.2 Hz, 2H, CH_2-Cy), 3.20 – 3.05 (m, 1H, Pip), 2.43 – 2.32 (m, 1H, C*H*-Cy), 1.96 – 1.53 (m, 11H, Pip, Cy), 1.30 – 1.13 (m, 3H, Pip, Cy), 1.11 – 0.92 (m, 2H, Pip, Cy). ^{13}C NMR (75 MHz, $CDCl_3$) δ (ppm): 173.99, 168.41, 136.42, 131.37, 126.59, 124.60, 122.61, 121.15, 120.72, 110.41, 109.46, 54.37, 53.43, 45.43, 38.68, 31.13, 26.30, 26.00, 25.74, 20.97. HRMS (ESI) *m/z* calcd. for $C_{22}H_{30}N_3O_2$ $[M+H]^+$: 368.2333; found: 368.2333.

(*R*)-1-(1-(cyclohexylmethyl)-1*H*-indole-3-carbonyl)piperidine-2-carboxamide (18f). Yield: 90%. ^{1}H NMR (300 MHz, $CDCl_3$) δ (ppm): 7.81 (dd, *J* = 7.5, 1.6 Hz, 1H, Ar-H), 7.42 (s, 1H, Ar-H), 7.40 – 7.35 (m, 1H, Ar-H), 7.33 – 7.19 (m, 2H, Ar-H), 6.83 (s, 1H, NH_2), 5.81 (s, 1H, NH_2), 5.28 – 5.19 (m, 1H, CH), 4.31 – 4.17 (m, 1H, Pip), 3.96 (d, *J* = 7.1 Hz, 2H, CH_2-Cy), 3.21 – 3.05 (m, 1H, Pip), 2.43 – 2.31 (m, 1H, C*H*-Cy), 1.83 – 1.55 (m, 11H, Pip, Cy), 1.36 – 1.11 (m, 3H, Pip, Cy), 1.10 – 0.95 (m, 2H, Pip, Cy). ^{13}C NMR (75 MHz, $CDCl_3$) δ (ppm): 173.89, 168.30, 136.30, 131.26, 126.47, 122.50, 121.04, 120.61, 110.30, 109.34, 54.01, 53.32, 45.38, 38.57, 31.02, 26.19, 25.89, 25.68, 25.63, 20.86. HRMS (ESI) *m/z* calcd. for $C_{22}H_{30}N_3O_2$ $[M+H]^+$: 368.2330; found: 368.2333.

(*S*)-1-(1-(4-fluorobenzyl)-1*H*-indole-3-carbonyl)piperidine-2-carboxamide (18g). Yield: 73%. ^{1}H NMR (300 MHz, $CDCl_3$) δ (ppm): 7.86 – 7.75 (m, 1H, Ar-H), 7.44 (s, 1H, Ar-H), 7.32 – 7.18 (m, 3H, Ar-H), 7.17 – 7.06 (m, 2H, Ar-H), 7.06 – 6.93 (m, 2H, Ar-H), 6.73 (s, 1H, NH_2), 5.70 (s, 1H, NH_2), 5.27 (s, 2H, CH_2-Ph), 5.24 – 5.15 (m, 1H, CH), 4.28 – 4.09 (m, 1H, Pip), 3.21 – 3.00 (m, 1H, Pip), 2.33 (d, *J* = 13.0 Hz, 1H, Pip), 1.94 – 1.51 (m, 5H, Pip). ^{13}C NMR (75 MHz, $CDCl_3$) δ (ppm): 173.87, 168.13, 162.57 (d, *J* = 247.1 Hz), 136.18, 132.04 (d, *J* = 3.3 Hz), 130.76, 128.88 (d, *J* = 8.1 Hz), 126.79, 123.09, 121.58, 120.84, 116.04 (d, *J* = 21.8 Hz), 110.49, 54.22, 49.95, 45.44, 40.07, 25.96, 25.87, 20.90. LRMS (ESI) *m/z* calcd. for $C_{22}H_{23}FN_3O_2$ $[M+H]^+$: 380.2; found: 380.2.

(*R*)-1-(1-(4-fluorobenzyl)-1*H*-indole-3-carbonyl)piperidine-2-carboxamide (18h). Yield: 79%. ^{1}H NMR (300 MHz, DMSO-*d6*) δ (ppm): 7.84 (s, 1H, Ar-H), 7.72 (d, *J* = 7.7 Hz, 1H, Ar-H), 7.51 (d, *J* = 7.9 Hz, 1H, Ar-H), 7.31 (dd, *J* = 9.2, 6.3 Hz, 3H, Ar-H), 7.22 – 7.08 (m, 5H, Ar-H, NH_2), 5.46 (s, 2H, CH_2-Ph), 5.07 – 4.90 (m, 1H, CH), 2.17 (d, *J* = 12.8 Hz, 1H, Pip), 1.72 – 1.54 (m, 3H, Pip), 1.53 – 1.32 (m, 2H, Pip), 1.32 – 1.19 (m, 2H, Pip). LRMS (ESI) *m/z* calcd. for $C_{22}H_{23}FN_3O_2$ $[M+H]^+$: 380.2; found: 380.2.

(*S*)-1-(1-pentyl-1*H*-indazole-3-carbonyl)piperidine-2-carboxamide (18i). Yield: 79%. ^{1}H NMR (300 MHz, $CDCl_3$) δ (ppm): 8.21 – 8.05 (m, 1H, Ar-H), 7.51 – 7.37 (m, 2H, Ar-H), 7.35 – 7.21 (m, 1H, Ar-H), 7.17 (s, 1H, NH_2), 6.57 (s, 1H, NH_2), 5.90 – 5.38 (m, 1H, CH), 4.76 (d, *J* = 13.6 Hz, 1H, Pip), 4.38 (t, *J* = 7.2 Hz, 2H, $CH_2CH_2CH_2CH_2CH_3$), 3.28 – 3.08 (m, 1H, Pip), 2.45 – 2.24 (m, 1H, Pip), 2.01 – 1.50 (m, 7H, Pip, $CH_2CH_2CH_2CH_2CH_3$), 1.44 – 1.20 (m, 4H, $CH_2CH_2CH_2CH_2CH_3$, $CH_2CH_2CH_2CH_2CH_3$), 0.86 (t, *J* = 6.8 Hz, 3H, $CH_2CH_2CH_2CH_2CH_3$). ^{13}C NMR (75 MHz, $CDCl_3$) δ (ppm): 173.65, 164.25, 138.81, 127.00, 124.51, 122.51, 109.32, 58.32, 53.15, 49.40, 45.75, 40.83, 29.44, 29.00, 26.56, 25.59, 22.29, 21.06, 14.00. HRMS (ESI) *m/z* calcd. for $C_{19}H_{27}N_4O_2$ $[M+H]^+$: 343.2129; found: 343.2123.

(*R*)-1-(1-pentyl-1*H*-indazole-3-carbonyl)piperidine-2-carboxamide (18j). Yield: 88%. ^{1}H NMR (300 MHz, $CDCl_3$) δ (ppm): 8.27 – 8.03 (m, 1H, Ar-H), 7.53 – 7.35 (m, 2H, Ar-H), 7.34 – 7.21 (m, 1H, Ar-H), 7.17 (s, 1H, NH_2), 6.57 (s, 1H, NH_2), 6.09 – 5.36 (m, 1H, CH), 4.75 (d, *J* = 13.5 Hz, 1H, Pip), 4.36 (t, *J* = 7.2 Hz, 2H, $CH_2CH_2CH_2CH_2CH_3$), 3.31 – 3.06 (m, 1H, Pip), 2.45 – 2.24 (m, 1H, Pip), 2.06 – 1.47 (m, 7H, Pip, $CH_2CH_2CH_2CH_2CH_3$), 1.44 – 1.15 (m, 4H, $CH_2CH_2CH_2CH_2CH_3$, $CH_2CH_2CH_2CH_2CH_3$), 0.85 (t, *J* = 6.7 Hz, 3H, $CH_2CH_2CH_2CH_2CH_3$). ^{13}C NMR (75 MHz, $CDCl_3$) δ (ppm): 173.65, 163.52, 138.82, 127.01, 124.52, 122.50, 109.32, 58.32, 53.13, 49.39, 45.73, 40.82, 29.44, 28.99, 26.54, 25.41, 22.28, 21.06, 14.00. HRMS (APCI) *m/z* calcd. for $C_{19}H_{27}N_4O_2$ $[M+H]^+$: 343.2129; found: 343.2127.

(*S*)-1-(1-(5-fluoropentyl)-1*H*-indazole-3-carbonyl)piperidine-2-carboxamide (18k). Yield: 77%. ^{1}H NMR (300 MHz, $CDCl_3$) δ (ppm): 8.27 – 8.04 (m, 1H, Ar-H), 7.52 – 7.37 (m, 2H, Ar-H), 7.35 – 7.21 (m, 1H, Ar-H), 7.08 (s, 1H, NH_2), 6.55 (s, 1H, NH_2), 5.82 – 5.37 (m, 1H, CH), 4.76 (d, *J* = 13.4 Hz, 1H, Pip), 4.48 (t, *J* = 5.9 Hz, 1H, $CH_2CH_2CH_2CH_2CH_2F$), 4.40 (t, *J* = 7.9 Hz, 2H, $CH_2CH_2CH_2CH_2CH_2F$), 4.33 (t, *J* = 5.9 Hz, 1H, $CH_2CH_2CH_2CH_2CH_2F$), 3.27 – 3.06 (m, 1H, Pip), 2.48 – 2.24 (m, 1H, Pip), 2.08 – 1.92 (m, 2H, $CH_2CH_2CH_2CH_2CH_2F$), 1.91 – 1.58 (m, 7H, $CH_2CH_2CH_2CH_2CH_2F$, Pip), 1.56 – 1.37 (m, 2H, $CH_2CH_2CH_2CH_2CH_2F$). LRMS (ESI) *m/z* calcd. for $C_{19}H_{26}FN_4O_2$ $[M+H]^+$: 361.2; found: 361.2.

(*R*)-1-(1-(5-fluoropentyl)-1*H*-indazole-3-carbonyl)piperidine-2-carboxamide (18l). Yield: 75%. ^{1}H NMR (300 MHz, $CDCl_3$) δ (ppm): 8.23 – 8.04 (m, 1H, Ar-H), 7.50 – 7.37 (m, 2H, Ar-H), 7.34 – 7.20 (m, 1H, Ar-H), 7.08 (s, 1H, NH_2), 6.56 (s, 1H, NH_2), 5.90 – 5.38 (m, 1H, CH), 4.75 (d, *J* = 13.5 Hz, 1H, Pip), 4.48 (t, *J* = 5.9 Hz, 1H, $CH_2CH_2CH_2CH_2CH_2F$), 4.40 (t, *J* = 7.8 Hz, 2H, $CH_2CH_2CH_2CH_2CH_2F$), 4.32 (t, *J* = 5.9 Hz, 1H, $CH_2CH_2CH_2CH_2CH_2F$), 3.28 – 3.08 (m, 1H, Pip), 2.46 – 2.24 (m, 1H, Pip), 2.06 – 1.89 (m, 2H, $CH_2CH_2CH_2CH_2CH_2F$), 1.85 – 1.57 (m, 7H, $CH_2CH_2CH_2CH_2CH_2F$, Pip), 1.53 – 1.34 (m, 2H, $CH_2CH_2CH_2CH_2CH_2F$). ^{13}C NMR (75 MHz, $CDCl_3$) δ (ppm): 173.65, 164.14, 138.88, 127.11, 124.49, 122.77, 122.55, 109.20, 83.77 (d, *J* = 164.9 Hz), 55.75, 49.10, 43.24, 29.95 (d, *J* = 19.9 Hz), 29.30, 26.59, 25.80, 25.24, 22.80 (d, *J* = 5.0 Hz), 21.02. LRMS (ESI) *m/z* calcd. for $C_{19}H_{26}FN_4O_2$ $[M+H]^+$: 361.2; found: 361.2.

(*S*)-1-(1-(cyclohexylmethyl)-1*H*-indazole-3-carbonyl)piperidine-2-carboxamide (18m). Yield: 84%. ^{1}H NMR (300 MHz, $CDCl_3$) δ (ppm): 8.23 – 8.00 (m, 1H, Ar-H), 7.49 – 7.33 (m, 2H, Ar-H), 7.32 – 7.23 (m, 1H, Ar-H), 7.18 (s, 1H, NH_2), 6.61 (s, 1H, NH_2), 5.93 – 5.35 (m, 1H, CH), 4.74 (d, *J* = 13.5 Hz, 1H, Pip), 4.20 (d, *J* = 7.5 Hz, 2H, CH_2-Cy), 3.25 – 3.10 (m, 1H, Pip), 2.50 – 2.21 (m, 1H, C*H*-Cy), 2.18 – 1.90 (m, 1H, Pip), 1.88 – 1.42 (m, 10H, Pip, Cy), 1.36 – 0.92 (m, 5H, Pip, Cy). ^{13}C NMR (75 MHz, $CDCl_3$) δ (ppm): 173.68, 163.52, 140.68, 137.34, 126.96, 124.36, 122.43, 109.55, 58.29, 55.66, 53.15, 45.73, 40.82, 38.73, 30.97, 29.79, 26.27, 25.71, 21.04. HRMS (APCI) *m/z* calc. for $C_{21}H_{29}N_4O_2$ $[M+H]^+$: 369.2285; found: 369.2288.

(*R*)-1-(1-(cyclohexylmethyl)-1*H*-indazole-3-carbonyl)piperidine-2-carboxamide (18n). Yield: 94%. ^{1}H NMR (300 MHz, $CDCl_3$) δ (ppm): 8.22 – 8.04 (m, 1H, Ar-H), 7.48 – 7.36 (m, 2H, Ar-H), 7.31 – 7.22 (m, 1H, Ar-H), 7.19 (s, 1H, NH_2), 6.58 (s, 1H, NH_2), 5.79 – 5.37 (m, 1H, CH), 4.76 (d, *J* = 13.6 Hz, 1H, Pip), 4.22 (d, *J* = 7.6 Hz, 2H, CH_2-Cy), 3.26 – 3.06 (m, 1H, Pip), 2.48 – 2.21 (m, 1H, C*H*-Cy), 2.09 – 1.85 (m, 1H, Pip), 1.84 – 1.39 (m, 10H, Pip, Cy), 1.31 – 0.93 (m, 5H, Pip, Cy). ^{13}C NMR (75 MHz, $CDCl_3$) δ (ppm): 173.67, 164.29, 140.71, 137.35, 127.00, 124.38, 122.46, 109.57, 58.30, 55.69, 53.18, 45.75, 40.82, 38.75, 30.99, 29.81, 26.28, 25.72, 21.05. LRMS (ESI) *m/z* calc. for $C_{21}H_{29}N_4O_2$ $[M+H]^+$: 369.2; found: 369.2.

(*S*)-1-(1-(4-fluorobenzyl)-1*H*-indazole-3-carbonyl)piperidine-2-carboxamide (18o). Yield: 85%. ^{1}H NMR (300 MHz, $CDCl_3$) δ (ppm): 8.25 – 8.01 (m, 1H, Ar-H), 7.47 – 7.32 (m, 2H, Ar-H), 7.31 – 7.10 (m, 3H, Ar-H), 7.06 – 6.96 (m, 2H, Ar-H), 6.95 (s, 1H, NH_2), 6.54 (s, 1H, NH_2) 5.98 – 5.68 (m, 1H, CH), 5.57 (s, 2H, CH_2-Ph), 5.54 – 5.37 (m, 1H, Pip), 4.73 (d, *J* = 13.5 Hz, 1H, Pip), 3.25 – 3.11 (m, 1H, Pip), 2.46 – 2.19 (m, 1H, Pip), 1.86 – 1.38 (m, 4H, Pip). ^{13}C NMR (75 MHz, $CDCl_3$) δ (ppm): 173.52, 167.57, 162.61 (d, *J* = 247.1 Hz), 139.21, 131.79 (d, *J* = 3.2 Hz), 129.33 (d, *J* = 8.1 Hz), 127.44, 124.80, 122.79, 115.93 (d, *J* = 21.7 Hz), 109.45, 58.35, 53.01, 45.75, 42.19, 40.83, 26.66, 25.42, 20.98. HRMS (ESI) *m/z* calc. for $C_{21}H_{22}FN_4O_2$ $[M+H]^+$: 381.1721; found: 381.1723.

(*R*)-1-(1-(4-fluorobenzyl)-1*H*-indazole-3-carbonyl)piperidine-2-carboxamide (18p). Yield: 80%. ^{1}H NMR (300 MHz, $CDCl_3$) δ (ppm): 8.22 – 8.05 (m, 1H, Ar-H), 7.47 – 7.33 (m, 2H, Ar-H), 7.31 – 7.13 (m, 3H, Ar-H), 7.05 – 6.97 (m, 2H, Ar-H), 6.95 (s, 1H, NH_2), 6.53 (s, 1H, NH_2), 5.82 – 5.63 (m, 1H, CH), 5.57 (s, 2H, CH_2-Ph), 5.55 – 5.38 (m, 1H, Pip), 4.73 (d, *J* = 13.4 Hz, 1H, Pip), 3.27 – 3.09 (m, 1H, Pip), 2.46 – 2.24 (m, 1H, Pip), 1.96 – 1.45 (m, 4H, Pip). ^{13}C NMR (75 MHz, $CDCl_3$) δ (ppm): 173.46, 166.61, 162.67 (d, *J* = 246.8 Hz), 139.25, 131.77 (d, *J* = 3.2 Hz), 129.35 (d, *J* = 8.1 Hz), 127.52, 124.85, 122.85, 115.98 (d, *J* = 21.9 Hz), 109.47, 58.38, 52.91, 45.76, 40.85, 38.76, 26.62, 25.39, 21.02. HRMS (ESI) *m/z* calc. for $C_{21}H_{22}FN_4O_2$ $[M+H]^+$: 381.1721; found: 381.1721.

Circular Dichroism

Given that the obtained derivatives contain essential features necessary for interaction with circularly polarized light, e.g. a chromophore (indole or indazole cores) and an enantiopure amino acid residue, circular dichroism (CD) spectroscopy was employed as part of the structural characterization of newly synthesized ligands. As illustrated in Supplementary Figure S1, each stereoisomer of the enantiomeric pairs exhibited symmetrical CD spectra, mirrored across the x-axis, with opposite signs (thus providing valuable stereochemical information), irrespective of the functional group (ester or amide) within the amino acid residue. The distinctive CD signal at ~240 nm allowed for an unambiguous assignment of the absolute configuration of each enantiomer: the (*S*) enantiomer displayed a negative Cotton effect (green line), whereas the (*R*) enantiomer produced a positive Cotton effect (purple line). These data provide valuable references for the unambiguous stereo-configuration assignment of analogous enantiopure series currently under development in our laboratories.

HPLC traces of representative enantiopure esters and amides.

```
=====================================================================
Injection Date  : 1/24/2024 6:19:50 AM          Seq. Line :  12
Sample Name     : 15f                            Location : Vial 64
Acq. Operator   : Sandra                              Inj :   1
Acq. Instrument : Instrument 1                 Inj Volume : 5 µl
Different Inj Volume from Sequence !     Actual Inj Volume : 10 µl
Acq. Method     : C:\HPCHEM\1\METHODS\QUIRAL\5P80L1.M
Last changed    : 3/30/2021 6:49:39 PM by ANDY
Analysis Method : C:\HPCHEM\1\METHODS\QUIRAL\APAGA.M
Last changed    : 1/29/2024 1:01:40 PM by Sandra
                  (modified after loading)
```

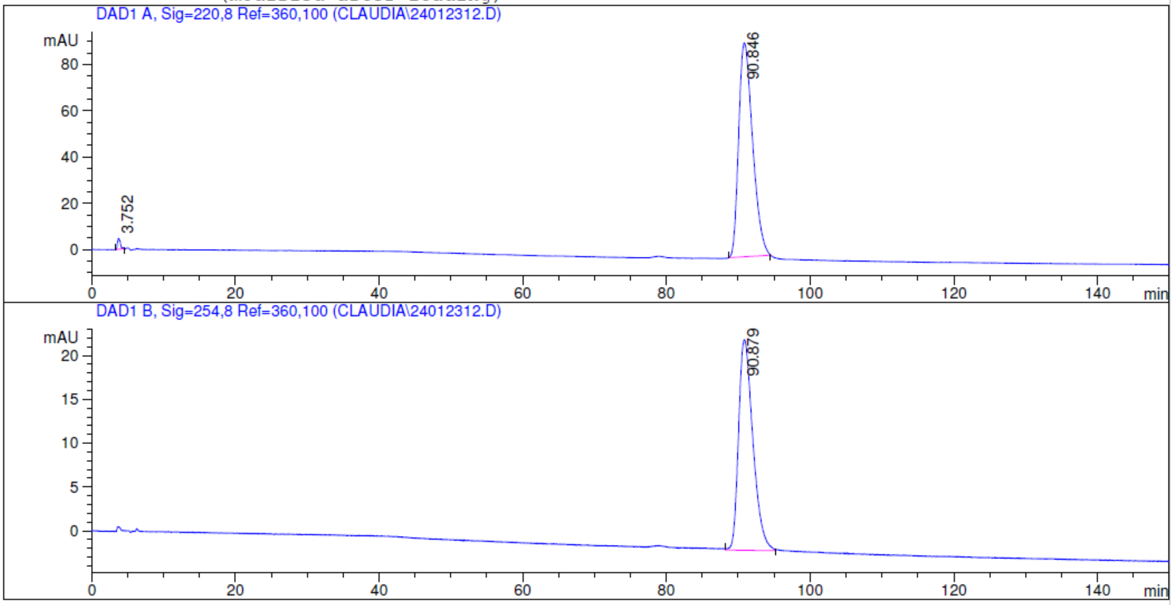


```
=====================================================================
                         Area Percent Report
=====================================================================

Sorted By             :      Signal
Multiplier            :      1.0000
Dilution              :      1.0000
Use Multiplier & Dilution Factor with ISTDs


Signal 1: DAD1 A, Sig=220,8 Ref=360,100
```

Peak #	RetTime [min]	Type	Width [min]	Area [mAU*s]	Height [mAU]	Area %
1	3.752	PB	0.3332	130.26195	4.73117	1.0338
2	90.846	BB	1.5987	1.24702e4	92.50934	98.9662

```
=====================================================================
Injection Date  : 8/22/2023 4:22:08 PM          Seq. Line :    4
Sample Name     : 15n                             Location : Vial 4
Acq. Operator   : Claudia                              Inj :    1
Acq. Instrument : Instrument 1                  Inj Volume : 5 µl
Acq. Method     : C:\HPCHEM\1\METHODS\QUIRAL\5P80M1.M
Last changed    : 8/22/2023 4:20:25 PM by Claudia
                  (modified after loading)
Analysis Method : C:\HPCHEM\1\METHODS\QUIRAL\APAGA.M
Last changed    : 1/29/2024 1:01:40 PM by Sandra
                  (modified after loading)
```

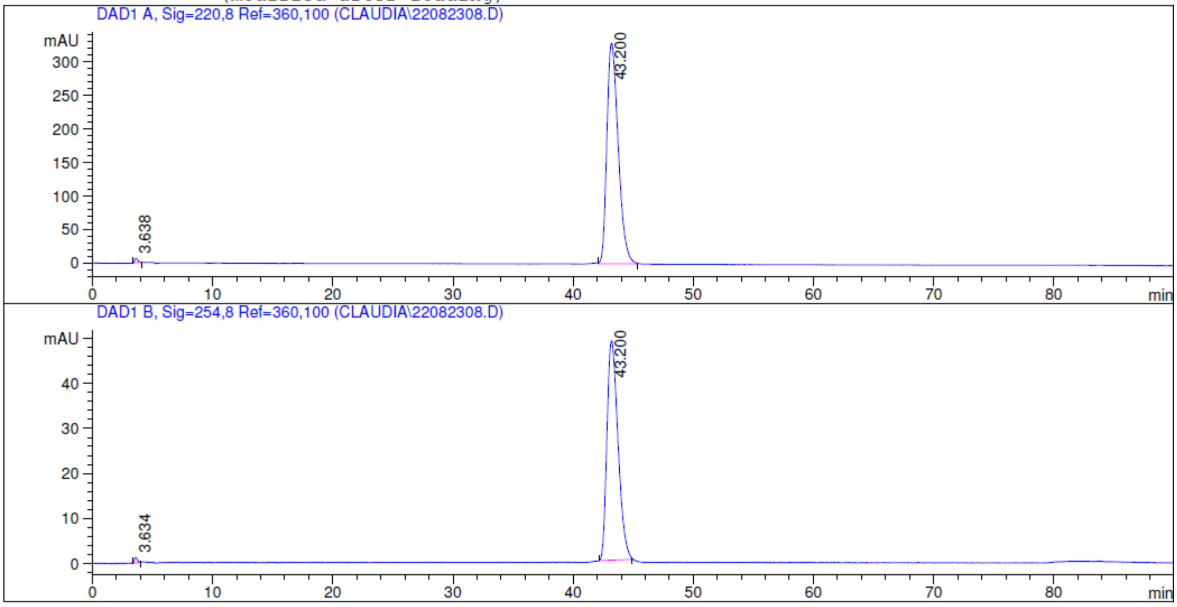


```
=====================================================================
                         Area Percent Report
=====================================================================

Sorted By             :      Signal
Multiplier            :      1.0000
Dilution              :      1.0000
Use Multiplier & Dilution Factor with ISTDs


Signal 1: DAD1 A, Sig=220,8 Ref=360,100

Peak RetTime Type  Width     Area      Height     Area
  #   [min]        [min]   [mAU*s]     [mAU]        %
----|-------|----|-------|----------|----------|--------|
   1   3.638 PB    0.2633  130.67140    6.76565   0.6013
   2  43.200 BB    0.9913 2.16011e4  329.31836  99.3987
```

```
=====================================================================
Injection Date  : 8/22/2023 2:29:11 PM          Seq. Line :    2
Sample Name     : 15m                            Location : Vial 3
Acq. Operator   : Claudia                             Inj :    1
Acq. Instrument : Instrument 1                 Inj Volume : 5 µl
Acq. Method     : C:\HPCHEM\1\METHODS\QUIRAL\5P80M1.M
Last changed    : 8/22/2023 2:27:30 PM by Claudia
                  (modified after loading)
Analysis Method : C:\HPCHEM\1\METHODS\QUIRAL\APAGA.M
Last changed    : 1/29/2024 1:01:40 PM by Sandra
                  (modified after loading)
```

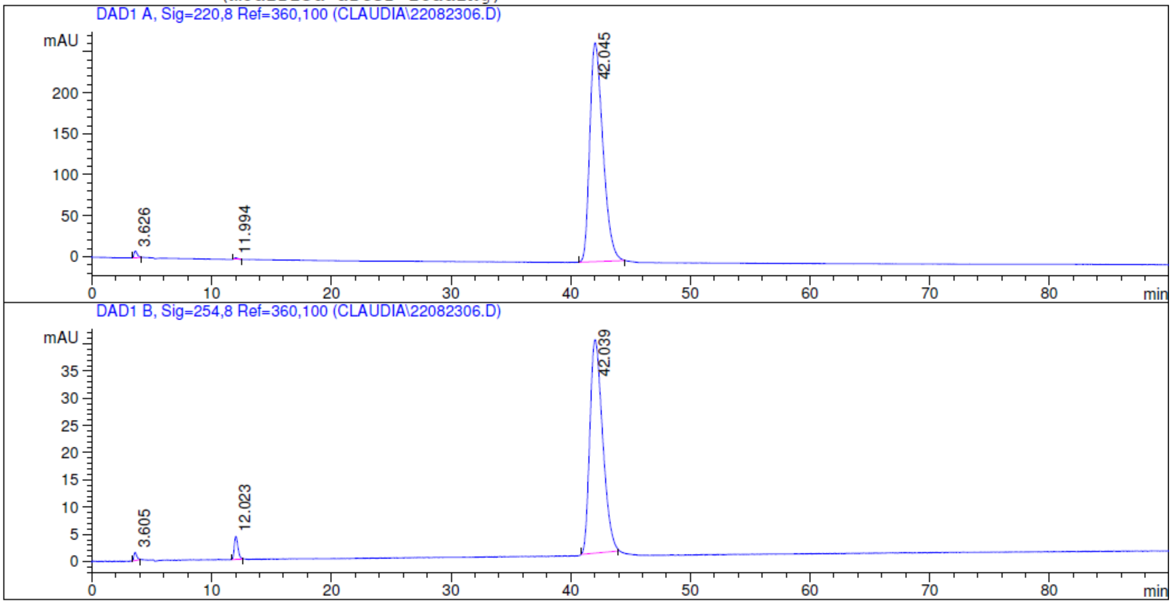


```
=====================================================================
                         Area Percent Report
=====================================================================

Sorted By             :      Signal
Multiplier            :      1.0000
Dilution              :      1.0000
Use Multiplier & Dilution Factor with ISTDs


Signal 1: DAD1 A, Sig=220,8 Ref=360,100
```

Peak #	RetTime [min]	Type	Width [min]	Area [mAU*s]	Height [mAU]	Area %
1	3.626	PB	0.2866	154.28966	8.15801	0.7491
2	11.994	BB	0.2582	39.75285	1.93097	0.1930
3	42.045	BB	1.1002	2.04030e4	266.78656	99.0579

```
=====================================================================
Injection Date  : 1/17/2024 5:45:39 AM           Seq. Line :  14
Sample Name     : 16c                             Location : Vial 67
Acq. Operator   : Sandra                               Inj :   1
Acq. Instrument : Instrument 1                  Inj Volume : 5 µl
Different Inj Volume from Sequence !      Actual Inj Volume : 10 µl
Acq. Method     : C:\HPCHEM\1\METHODS\QUIRAL\5P80M1.M
Last changed    : 1/16/2024 6:03:18 PM by Sandra
Analysis Method : C:\HPCHEM\1\METHODS\QUIRAL\APAGA.M
Last changed    : 1/29/2024 1:01:40 PM by Sandra
                  (modified after loading)
```

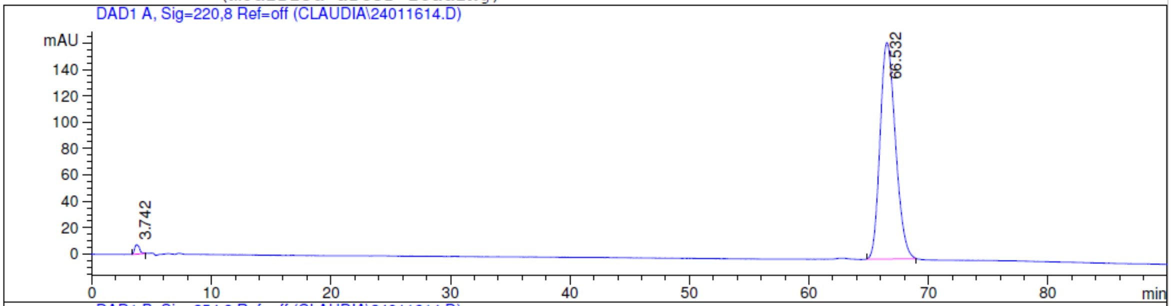

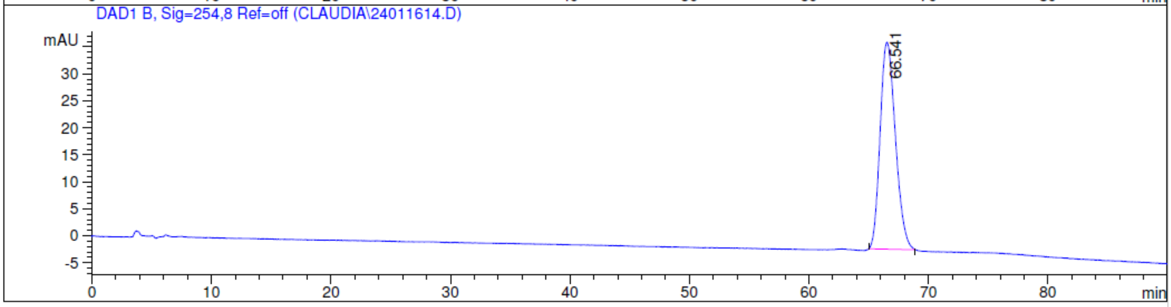


```
=====================================================================
                         Area Percent Report
=====================================================================

Sorted By             :      Signal
Multiplier            :      1.0000
Dilution              :      1.0000
Use Multiplier & Dilution Factor with ISTDs


Signal 1: DAD1 A, Sig=220,8 Ref=off
```

Peak #	RetTime [min]	Type	Width [min]	Area [mAU*s]	Height [mAU]	Area %
1	3.742	BB	0.3784	199.84081	7.10011	1.3368
2	66.532	BB	1.2688	1.47497e4	164.07025	98.6632

```
=====================================================================
Injection Date   : 1/17/2024 7:38:48 AM          Seq. Line :  16
Sample Name      : 16d                            Location : Vial 68
Acq. Operator    : Sandra                              Inj :   1
Acq. Instrument  : Instrument 1                 Inj Volume : 5 µl
Different Inj Volume from Sequence !     Actual Inj Volume : 10 µl
Acq. Method      : C:\HPCHEM\1\METHODS\QUIRAL\5P80M1.M
Last changed     : 1/16/2024 6:03:18 PM by Sandra
Analysis Method  : C:\HPCHEM\1\METHODS\QUIRAL\APAGA.M
Last changed     : 1/29/2024 1:01:40 PM by Sandra
                   (modified after loading)
```

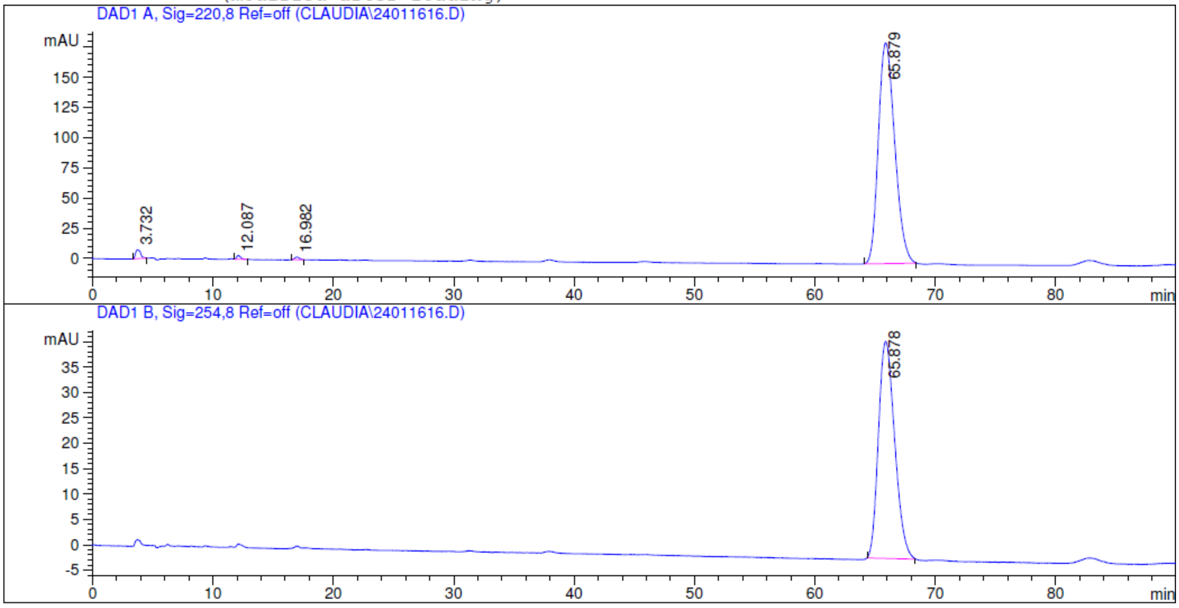


```
=====================================================================
                         Area Percent Report
=====================================================================

Sorted By             :      Signal
Multiplier            :      1.0000
Dilution              :      1.0000
Use Multiplier & Dilution Factor with ISTDs


Signal 1: DAD1 A, Sig=220,8 Ref=off
```

Peak #	RetTime [min]	Type	Width [min]	Area [mAU*s]	Height [mAU]	Area %
1	3.732	BB	0.3658	216.85683	7.44578	1.2288
2	12.087	PP	0.2823	68.82484	3.01526	0.3900
3	16.982	BB	0.3229	60.40960	2.23836	0.3423
4	65.879	BB	1.3129	1.73012e4	182.69205	98.0388

```
=====================================================================
Injection Date  : 1/18/2024 2:30:45 AM           Seq. Line :  36
Sample Name     : 16i                              Location : Vial 78
Acq. Operator   : Sandra                                Inj :   1
Acq. Instrument : Instrument 1                   Inj Volume : 5 µl
Different Inj Volume from Sequence !      Actual Inj Volume : 10 µl
Acq. Method     : C:\HPCHEM\1\METHODS\QUIRAL\5P80M1.M
Last changed    : 1/16/2024 6:03:18 PM by Sandra
Analysis Method : C:\HPCHEM\1\METHODS\QUIRAL\APAGA.M
Last changed    : 1/29/2024 1:01:40 PM by Sandra
                  (modified after loading)
```

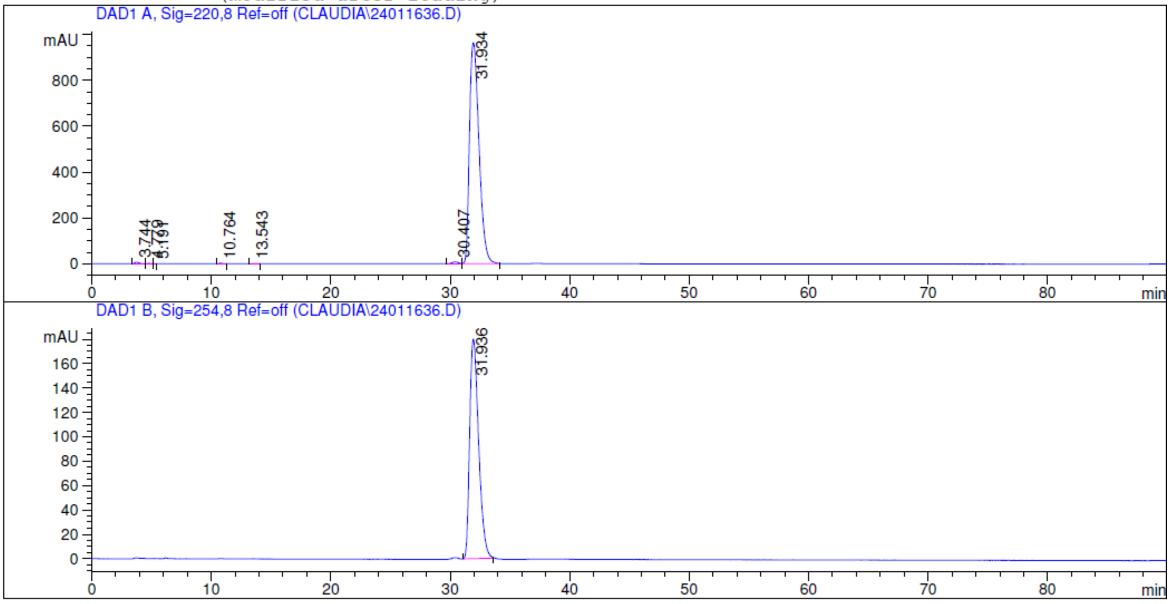


```
=====================================================================
                         Area Percent Report
=====================================================================

Sorted By             :      Signal
Multiplier            :      1.0000
Dilution              :      1.0000
Use Multiplier & Dilution Factor with ISTDs


Signal 1: DAD1 A, Sig=220,8 Ref=off
```

Peak #	RetTime [min]	Type	Width [min]	Area [mAU*s]	Height [mAU]	Area %
1	3.744	PB	0.3839	176.01288	5.97097	0.3266
2	4.779	BV	0.3251	44.19925	1.61611	0.0820
3	5.191	VB	0.1478	12.67411	1.10513	0.0235
4	10.764	BB	0.2622	53.51774	2.59954	0.0993
5	13.543	PP	0.2873	28.87478	1.25082	0.0536
6	30.407	PV	0.4692	304.15909	7.89882	0.5644
7	31.934	VB	0.8484	5.32732e4	964.50116	98.8506

```
=====================================================================
Injection Date  : 1/23/2024 6:46:57 PM           Seq. Line :    4
Sample Name     : 17g                             Location : Vial 81
Acq. Operator   : Sandra                               Inj :    1
Acq. Instrument : Instrument 1                  Inj Volume : 5 µl
Different Inj Volume from Sequence !     Actual Inj Volume : 10 µl
Acq. Method     : C:\HPCHEM\1\METHODS\QUIRAL\5P80L1.M
Last changed    : 3/30/2021 6:49:39 PM by ANDY
Analysis Method : C:\HPCHEM\1\METHODS\QUIRAL\APAGA.M
Last changed    : 1/29/2024 1:01:40 PM by Sandra
                  (modified after loading)
```

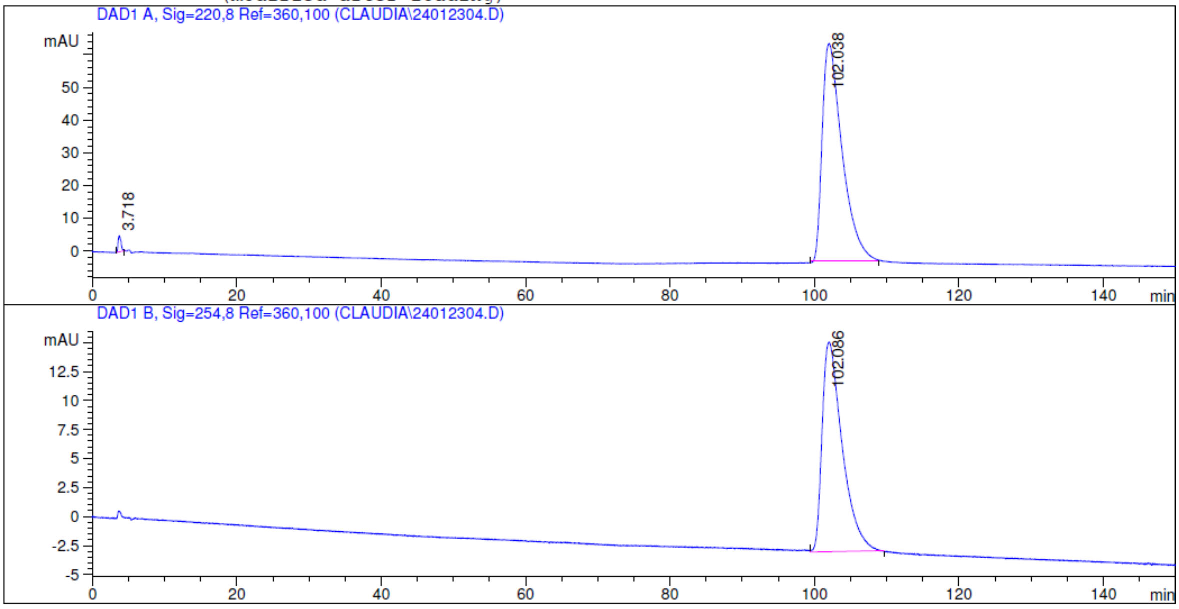


```
=====================================================================
                         Area Percent Report
=====================================================================

Sorted By             :      Signal
Multiplier            :      1.0000
Dilution              :      1.0000
Use Multiplier & Dilution Factor with ISTDs


Signal 1: DAD1 A, Sig=220,8 Ref=360,100

Peak RetTime Type  Width     Area      Height     Area
  #   [min]        [min]   [mAU*s]     [mAU]        %
----|-------|----|-------|----------|----------|--------|
   1   3.718 PB    0.3754  135.69716    4.92651   1.0551
   2 102.038 MM    3.1897 1.27259e4   66.49432  98.9449
```

```
==================================================================
Injection Date  : 1/18/2024 12:37:36 AM          Seq. Line :  34
Sample Name     : 16j                             Location : Vial 77
Acq. Operator   : Sandra                               Inj :   1
Acq. Instrument : Instrument 1                  Inj Volume : 5 µl
Different Inj Volume from Sequence !     Actual Inj Volume : 10 µl
Acq. Method     : C:\HPCHEM\1\METHODS\QUIRAL\5P80M1.M
Last changed    : 1/16/2024 6:03:18 PM by Sandra
Analysis Method : C:\HPCHEM\1\METHODS\QUIRAL\APAGA.M
Last changed    : 1/29/2024 1:01:40 PM by Sandra
                  (modified after loading)
```

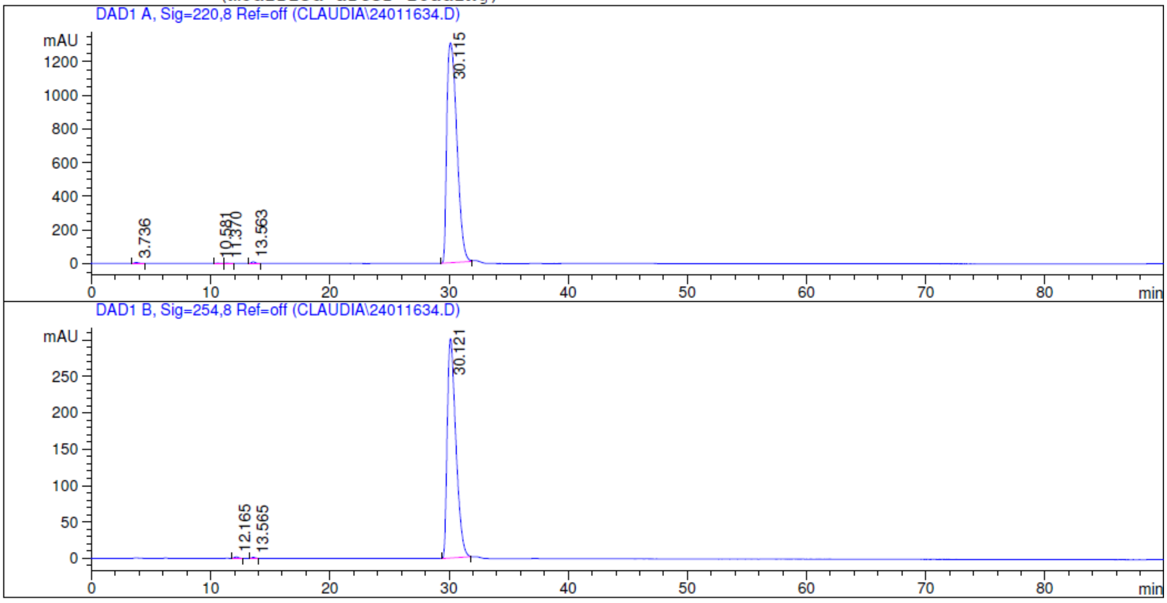


```
==================================================================
                         Area Percent Report
==================================================================

Sorted By             :      Signal
Multiplier            :      1.0000
Dilution              :      1.0000
Use Multiplier & Dilution Factor with ISTDs


Signal 1: DAD1 A, Sig=220,8 Ref=off
```

Peak #	RetTime [min]	Type	Width [min]	Area [mAU*s]	Height [mAU]	Area %
1	3.736	BP	0.3441	153.65477	5.63477	0.1997
2	10.581	PB	0.2626	46.80105	2.36814	0.0608
3	11.370	BP	0.2310	45.49364	2.51586	0.0591
4	13.563	BB	0.3383	220.16325	9.36539	0.2861
5	30.115	PB	0.6979	7.64774e4	1308.39221	99.3942

```
======================================================================
Injection Date  : 1/25/2024 4:21:12 PM           Seq. Line :    4
Sample Name     : 17h                             Location : Vial 83
Acq. Operator   : Sandra                               Inj :    1
Acq. Instrument : Instrument 1                  Inj Volume : 5 µl
Different Inj Volume from Sequence !     Actual Inj Volume : 10 µl
Acq. Method     : C:\HPCHEM\1\METHODS\QUIRAL\5P80L1.M
Last changed    : 3/30/2021 6:49:39 PM by ANDY
Analysis Method : C:\HPCHEM\1\METHODS\QUIRAL\APAGA.M
Last changed    : 1/29/2024 1:01:40 PM by Sandra
                  (modified after loading)
```

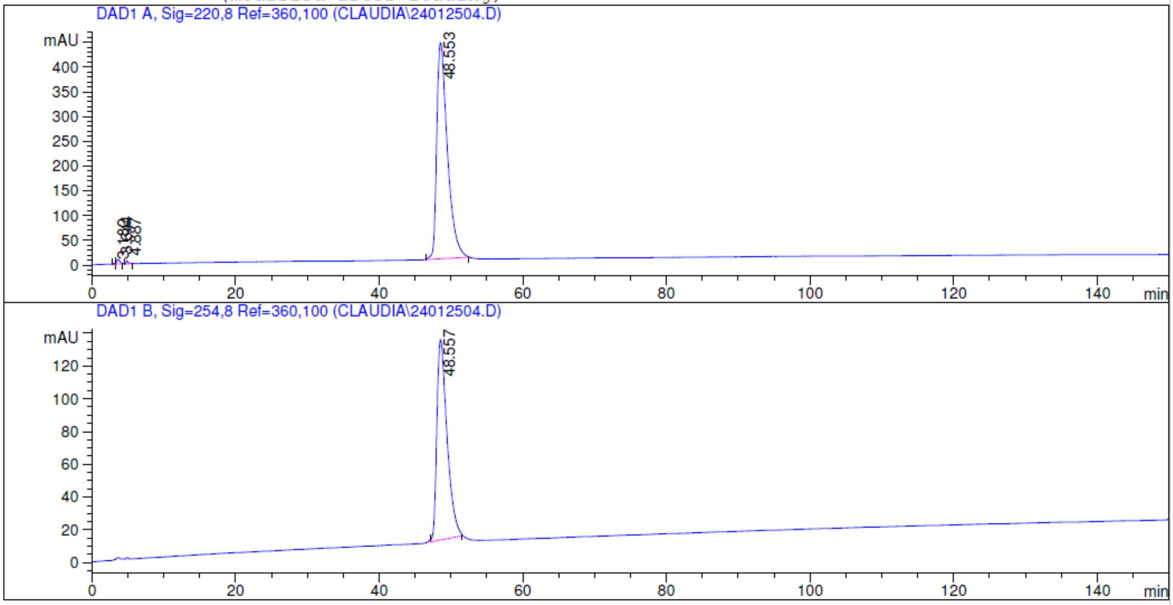


```
======================================================================
                         Area Percent Report
======================================================================

Sorted By             :      Signal
Multiplier            :      1.0000
Dilution              :      1.0000
Use Multiplier & Dilution Factor with ISTDs


Signal 1: DAD1 A, Sig=220,8 Ref=360,100

Peak RetTime Type  Width     Area      Height     Area
  #   [min]        [min]   [mAU*s]     [mAU]       %
----|-------|----|-------|----------|----------|--------|
   1   3.182 BV    0.1961   25.87353    1.62176   0.0569
   2   3.644 VV    0.3803  291.44177    9.28137   0.6407
   3   4.887 PB    0.2874  127.46597    6.26370   0.2802
   4  48.553 BB    1.4046 4.50416e4  436.95044  99.0222
```

```
=====================================================================
Injection Date  : 1/24/2024 12:33:27 AM          Seq. Line :   8
Sample Name     : 17k                             Location : Vial 62
Acq. Operator   : Sandra                               Inj :   1
Acq. Instrument : Instrument 1                  Inj Volume : 5 µl
Different Inj Volume from Sequence !     Actual Inj Volume : 10 µl
Acq. Method     : C:\HPCHEM\1\METHODS\QUIRAL\5P80L1.M
Last changed    : 3/30/2021 6:49:39 PM by ANDY
Analysis Method : C:\HPCHEM\1\METHODS\QUIRAL\APAGA.M
Last changed    : 1/29/2024 1:01:40 PM by Sandra
                  (modified after loading)
```

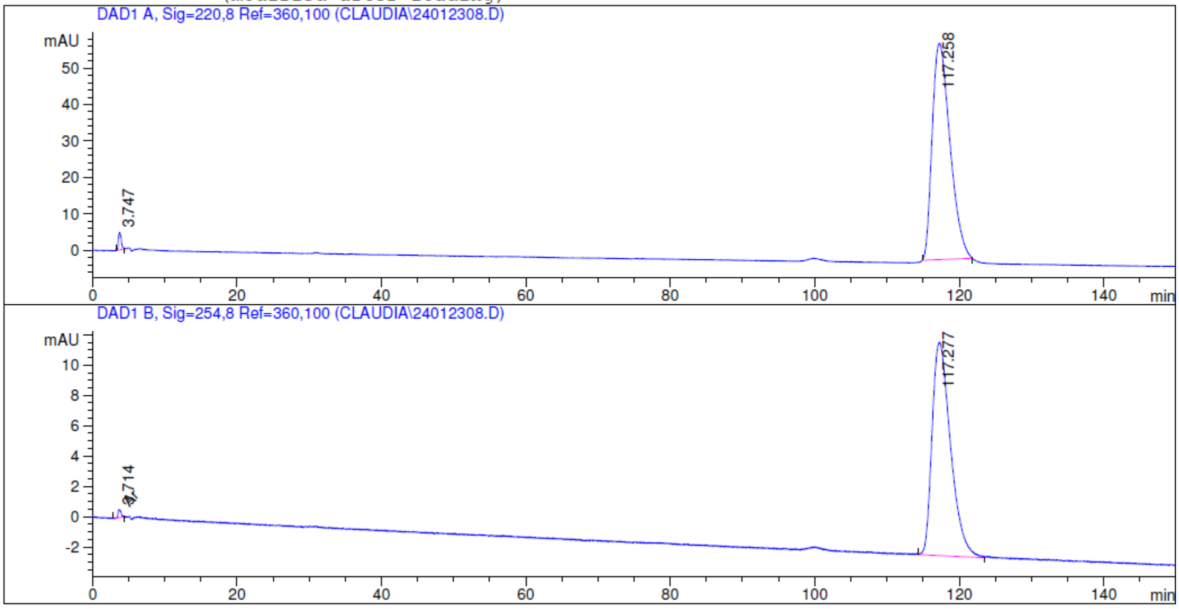


```
=====================================================================
                         Area Percent Report
=====================================================================

Sorted By             :      Signal
Multiplier            :      1.0000
Dilution              :      1.0000
Use Multiplier & Dilution Factor with ISTDs


Signal 1: DAD1 A, Sig=220,8 Ref=360,100

Peak RetTime Type  Width     Area      Height     Area
  #   [min]        [min]   [mAU*s]     [mAU]        %
----|-------|----|-------|----------|----------|--------|
   1   3.747 PB    0.3352  129.22272    4.81328   1.2583
   2 117.258 BB    2.0018 1.01403e4    59.33695  98.7417
```

```
==================================================================
Injection Date  : 1/23/2024 9:40:15 PM           Seq. Line :   6
Sample Name     : 171                             Location : Vial 61
Acq. Operator   : Sandra                               Inj :   1
Acq. Instrument : Instrument 1                  Inj Volume : 5 µl
Different Inj Volume from Sequence !     Actual Inj Volume : 10 µl
Acq. Method     : C:\HPCHEM\1\METHODS\QUIRAL\5P80L1.M
Last changed    : 3/30/2021 6:49:39 PM by ANDY
Analysis Method : C:\HPCHEM\1\METHODS\QUIRAL\APAGA.M
Last changed    : 1/29/2024 1:01:40 PM by Sandra
                  (modified after loading)
```

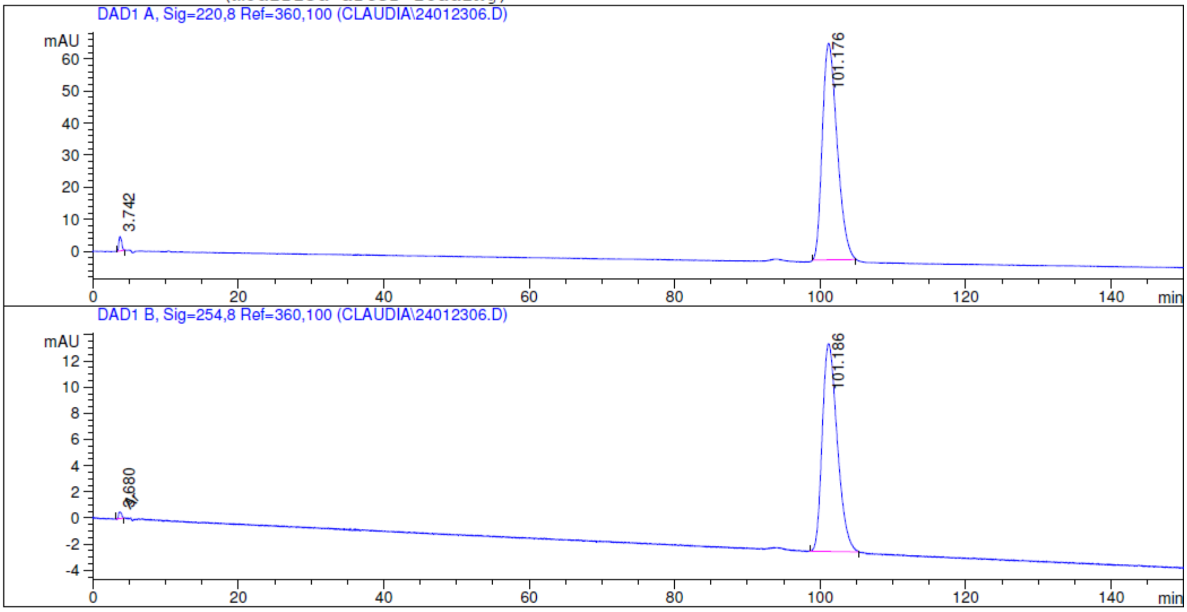


```
==================================================================
                        Area Percent Report
==================================================================

Sorted By             :      Signal
Multiplier            :      1.0000
Dilution              :      1.0000
Use Multiplier & Dilution Factor with ISTDs


Signal 1: DAD1 A, Sig=220,8 Ref=360,100
```

Peak #	RetTime [min]	Type	Width [min]	Area [mAU*s]	Height [mAU]	Area %
1	3.742	BB	0.3253	120.83051	4.55768	1.2502
2	101.176	BB	1.6810	9544.15527	67.52505	98.7498

```
====================================================================
Injection Date  : 1/17/2024 3:52:29 AM           Seq. Line :  12
Sample Name     : 18e                              Location : Vial 66
Acq. Operator   : Sandra                                Inj :   1
Acq. Instrument : Instrument 1                   Inj Volume : 5 µl
Different Inj Volume from Sequence !      Actual Inj Volume : 10 µl
Acq. Method     : C:\HPCHEM\1\METHODS\QUIRAL\5P80M1.M
Last changed    : 1/16/2024 6:03:18 PM by Sandra
Analysis Method : C:\HPCHEM\1\METHODS\QUIRAL\APAGA.M
Last changed    : 1/29/2024 1:01:40 PM by Sandra
                  (modified after loading)
```

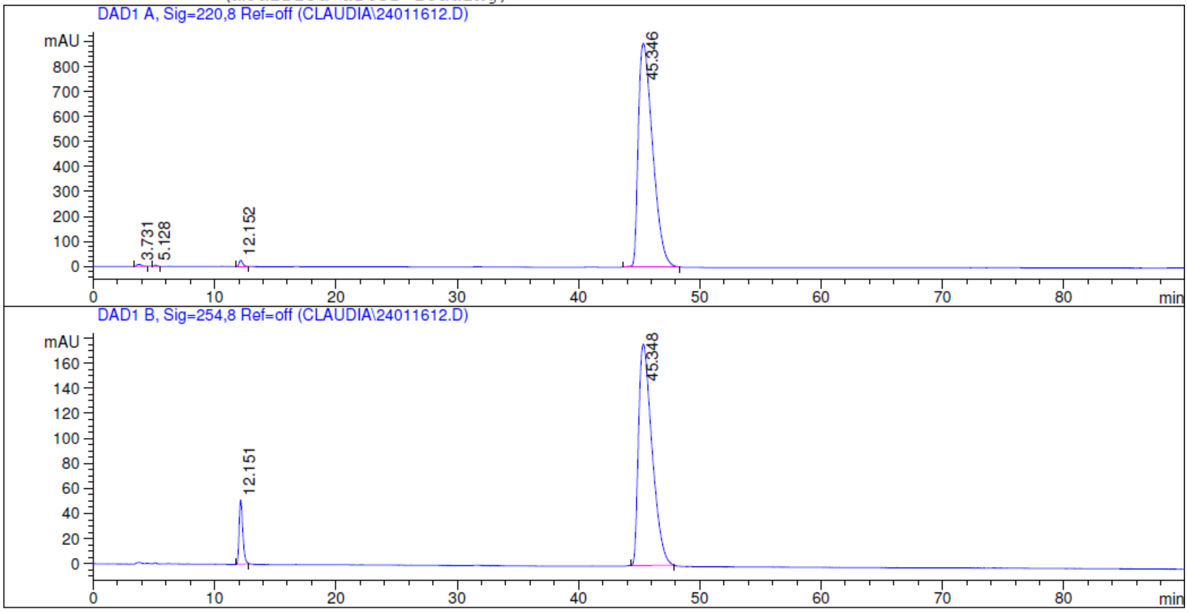


```
====================================================================
                         Area Percent Report
====================================================================

Sorted By             :      Signal
Multiplier            :      1.0000
Dilution              :      1.0000
Use Multiplier & Dilution Factor with ISTDs


Signal 1: DAD1 A, Sig=220,8 Ref=off
```

Peak #	RetTime [min]	Type	Width [min]	Area [mAU*s]	Height [mAU]	Area %
1	3.731	PB	0.3533	223.88268	7.83590	0.3018
2	5.128	BP	0.2290	74.66684	4.97740	0.1006
3	12.152	PB	0.3154	522.69836	25.05079	0.7046
4	45.346	BB	0.9715	7.33659e4	895.78760	98.8930

```
=====================================================================
Injection Date  : 1/17/2024 1:59:19 AM          Seq. Line :  10
Sample Name     : 18f                            Location : Vial 65
Acq. Operator   : Sandra                              Inj :   1
Acq. Instrument : Instrument 1                 Inj Volume : 5 µl
Different Inj Volume from Sequence !     Actual Inj Volume : 10 µl
Acq. Method     : C:\HPCHEM\1\METHODS\QUIRAL\5P80M1.M
Last changed    : 1/16/2024 6:03:18 PM by Sandra
Analysis Method : C:\HPCHEM\1\METHODS\QUIRAL\APAGA.M
Last changed    : 1/29/2024 1:01:40 PM by Sandra
                  (modified after loading)
```

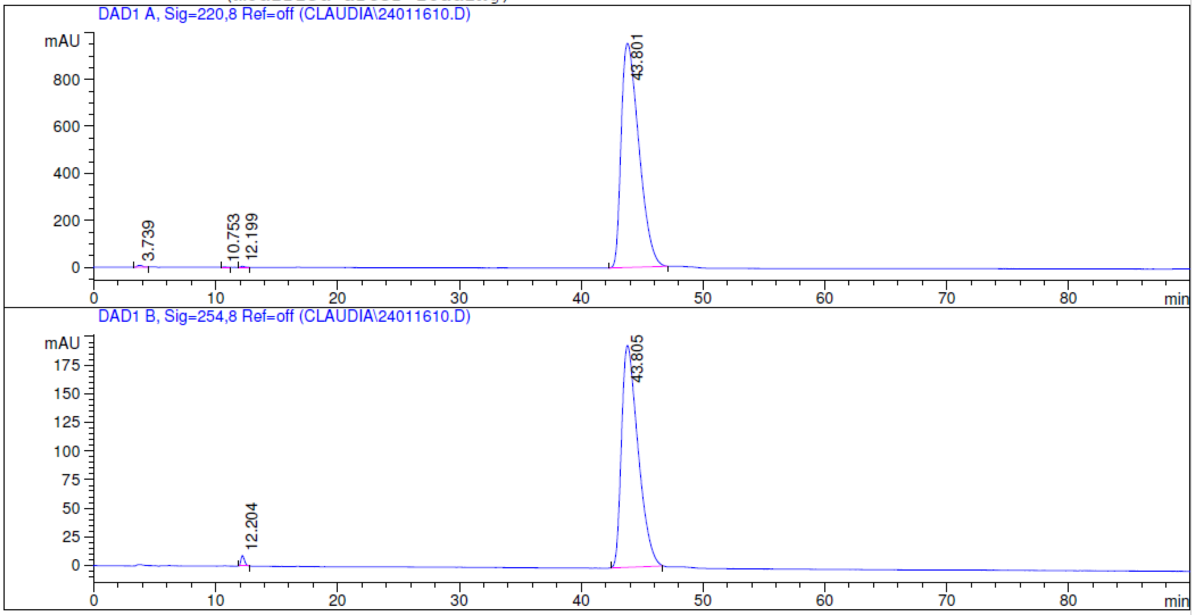


```
=====================================================================
                         Area Percent Report
=====================================================================

Sorted By             :      Signal
Multiplier            :      1.0000
Dilution              :      1.0000
Use Multiplier & Dilution Factor with ISTDs


Signal 1: DAD1 A, Sig=220,8 Ref=off
```

Peak #	RetTime [min]	Type	Width [min]	Area [mAU*s]	Height [mAU]	Area %
1	3.739	PP	0.3717	231.11781	8.03190	0.2387
2	10.753	BB	0.2488	33.14703	1.66113	0.0342
3	12.199	PB	0.2873	92.44038	4.39586	0.0955
4	43.801	BB	1.2223	9.64592e4	955.06482	99.6316

```
==================================================================
Injection Date  : 1/17/2024 9:32:07 AM          Seq. Line :  18
Sample Name     : 18o                            Location : Vial 69
Acq. Operator   : Sandra                              Inj :   1
Acq. Instrument : Instrument 1                 Inj Volume : 5 µl
Different Inj Volume from Sequence !    Actual Inj Volume : 10 µl
Acq. Method     : C:\HPCHEM\1\METHODS\QUIRAL\5P80M1.M
Last changed    : 1/16/2024 6:03:18 PM by Sandra
Analysis Method : C:\HPCHEM\1\METHODS\QUIRAL\APAGA.M
Last changed    : 1/29/2024 1:01:40 PM by Sandra
                  (modified after loading)
```

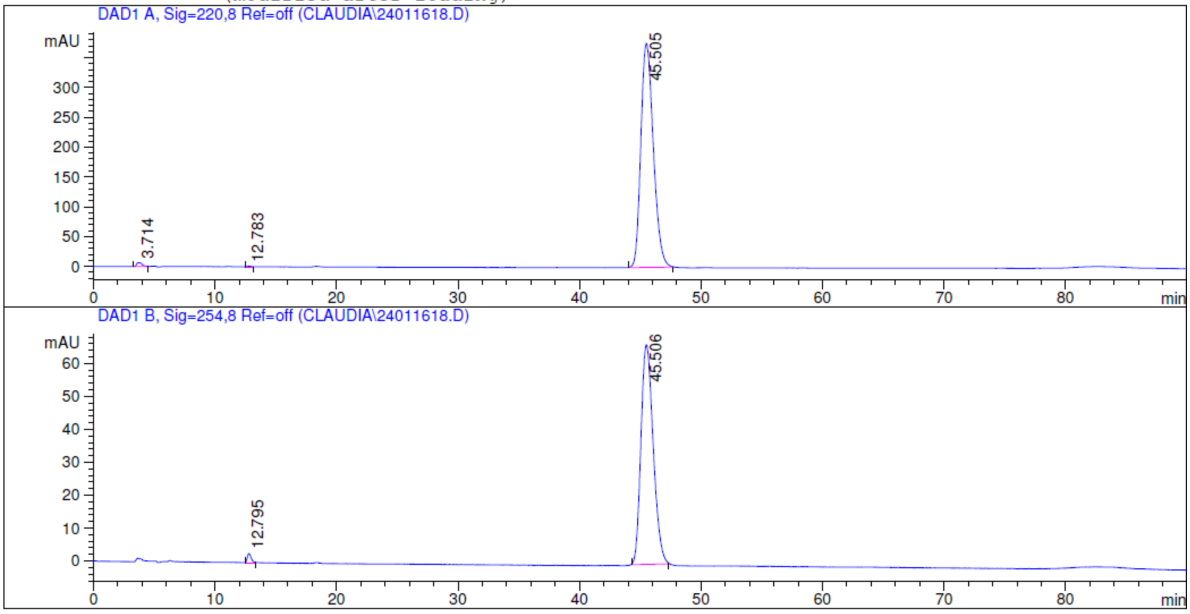


```
==================================================================
                         Area Percent Report
==================================================================

Sorted By             :      Signal
Multiplier            :      1.0000
Dilution              :      1.0000
Use Multiplier & Dilution Factor with ISTDs


Signal 1: DAD1 A, Sig=220,8 Ref=off
```

Peak #	RetTime [min]	Type	Width [min]	Area [mAU*s]	Height [mAU]	Area %
1	3.714	PB	0.4117	195.11958	6.50864	0.7236
2	12.783	PB	0.2550	28.72019	1.35638	0.1065
3	45.505	BB	1.0376	2.67400e4	375.14017	99.1699

```
=====================================================================
Injection Date  : 1/17/2024 11:25:41 AM          Seq. Line :  20
Sample Name     : 18p                             Location : Vial 70
Acq. Operator   : Sandra                               Inj :   1
Acq. Instrument : Instrument 1                  Inj Volume : 5 µl
Different Inj Volume from Sequence !     Actual Inj Volume : 10 µl
Acq. Method     : C:\HPCHEM\1\METHODS\QUIRAL\5P80M1.M
Last changed    : 1/16/2024 6:03:18 PM by Sandra
Analysis Method : C:\HPCHEM\1\METHODS\QUIRAL\APAGA.M
Last changed    : 1/29/2024 1:01:40 PM by Sandra
                  (modified after loading)
```

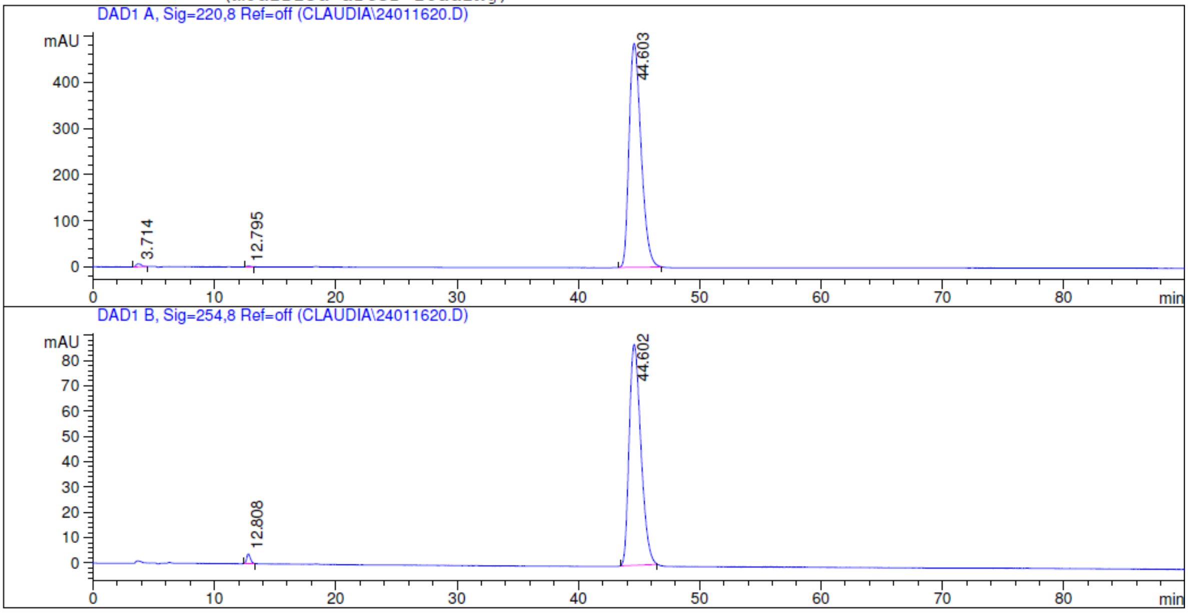


```
=====================================================================
                         Area Percent Report
=====================================================================

Sorted By             :      Signal
Multiplier            :      1.0000
Dilution              :      1.0000
Use Multiplier & Dilution Factor with ISTDs


Signal 1: DAD1 A, Sig=220,8 Ref=off
```

Peak #	RetTime [min]	Type	Width [min]	Area [mAU*s]	Height [mAU]	Area %
1	3.714	PB	0.3623	189.68976	6.46451	0.5662
2	12.795	BP	0.2568	38.06348	1.81401	0.1136
3	44.603	BB	1.0160	3.32717e4	485.22858	99.3201

HPLC purity analysis

SAMPLE INFORMATION			
Sample Name:	15a	Acquired By:	System
Sample Type:	Unknown	Sample Set Name:	Analitic
Vial:	90	Acq. Method Set:	5_95%B_20min_P3
Injection #:	1	Processing Method:	Amidas cíclicas
Injection Volume:	5,00 ul	Channel Name:	254+280nm@5
Run Time:	20,0 Minutes	Proc. Chnl. Descr.:	2998 PDA Calculated: 254+280
Date Acquired:	07/12/2024 13:02:25 CET		
Date Processed:	07/12/2024 14:21:37 CET		

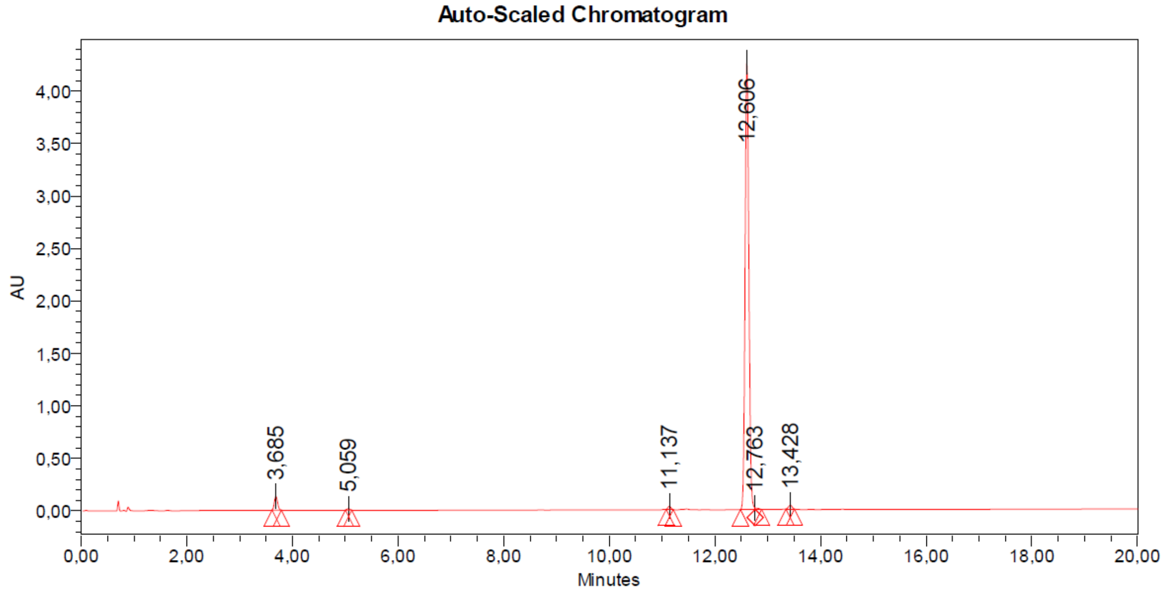

Peak Results

	RT	Area	% Area	Height
1	3,685	559971	2,51	131522
2	5,059	53141	0,24	13630
3	11,137	117648	0,53	29798
4	12,606	21343610	95,71	4264724
5	12,763	55066	0,25	14423
6	13,428	170409	0,76	36507

SAMPLE INFORMATION

Sample Name:	15h	Acquired By:	System
Sample Type:	Unknown	Sample Set Name:	Analitic
Vial:	87	Acq. Method Set:	5_95%B_20min_P3
Injection #:	1	Processing Method:	Amidas cíclicas
Injection Volume:	15,00 ul	Channel Name:	254+280nm
Run Time:	20,0 Minutes	Proc. Chnl. Descr.:	2998 PDA Calculated: 254+280
Date Acquired:	06/12/2024 15:25:27 CET		
Date Processed:	07/12/2024 14:36:55 CET		

Auto-Scaled Chromatogram

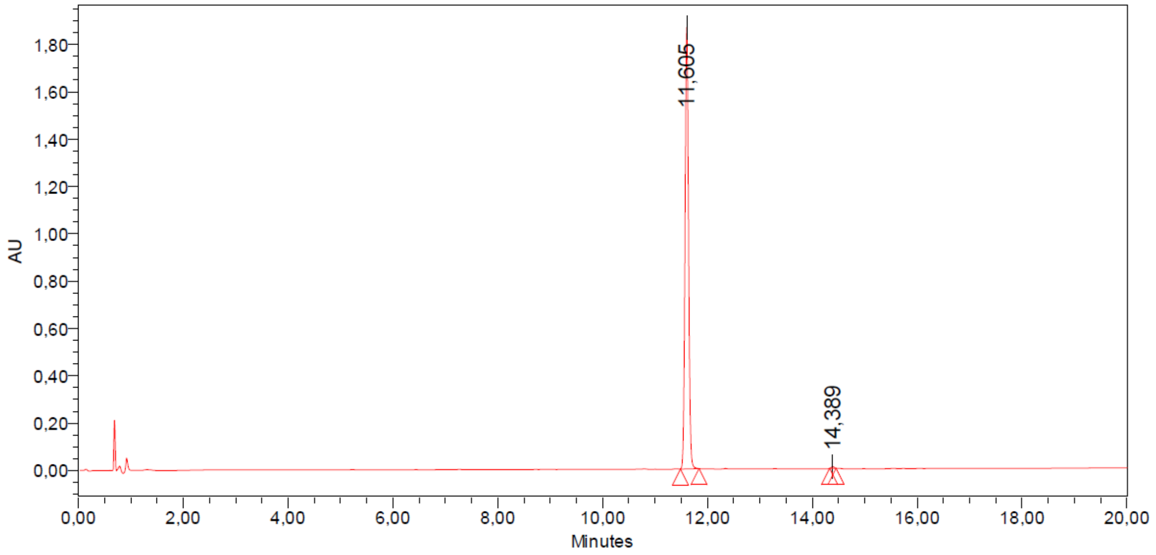

Peak Results

	RT	Area	% Area	Height
1	11,605	8612869	99,67	1866140
2	14,389	28599	0,33	7305

SAMPLE INFORMATION

Sample Name:	15i	Acquired By:	System
Sample Type:	Unknown	Sample Set Name:	Analitic
Vial:	89	Acq. Method Set:	5_95%B_20min_P3
Injection #:	1	Processing Method:	Amidas cíclicas
Injection Volume:	5,00 ul	Channel Name:	254+280nm@2
Run Time:	20,0 Minutes	Proc. Chnl. Descr.:	2998 PDA Calculated: 254+280
Date Acquired:	07/12/2024 12:35:52 CET		
Date Processed:	07/12/2024 14:34:38 CET		

Auto-Scaled Chromatogram

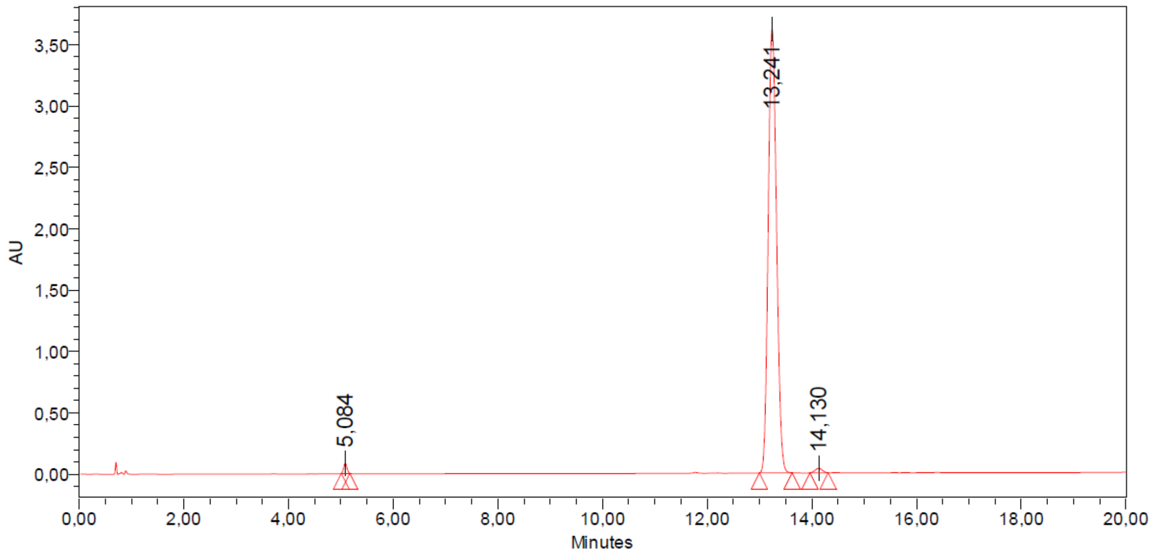

Peak Results

	RT	Area	% Area	Height
1	5,084	366243	0,90	82205
2	13,241	39870140	98,23	3616752
3	14,130	353790	0,87	35612

S A M P L E   I N F O R M A T I O N

Sample Name:	15l	Acquired By:	System
Sample Type:	Unknown	Sample Set Name:	Analitic
Vial:	88	Acq. Method Set:	5_95%B_20min_P3
Injection #:	1	Processing Method:	Amidas cíclicas
Injection Volume:	5,00 ul	Channel Name:	254+280nm
Run Time:	20,0 Minutes	Proc. Chnl. Descr.:	2998 PDA Calculated: 254+280
Date Acquired:	06/12/2024 16:18:03 CET		
Date Processed:	07/12/2024 14:40:12 CET		

Auto-Scaled Chromatogram

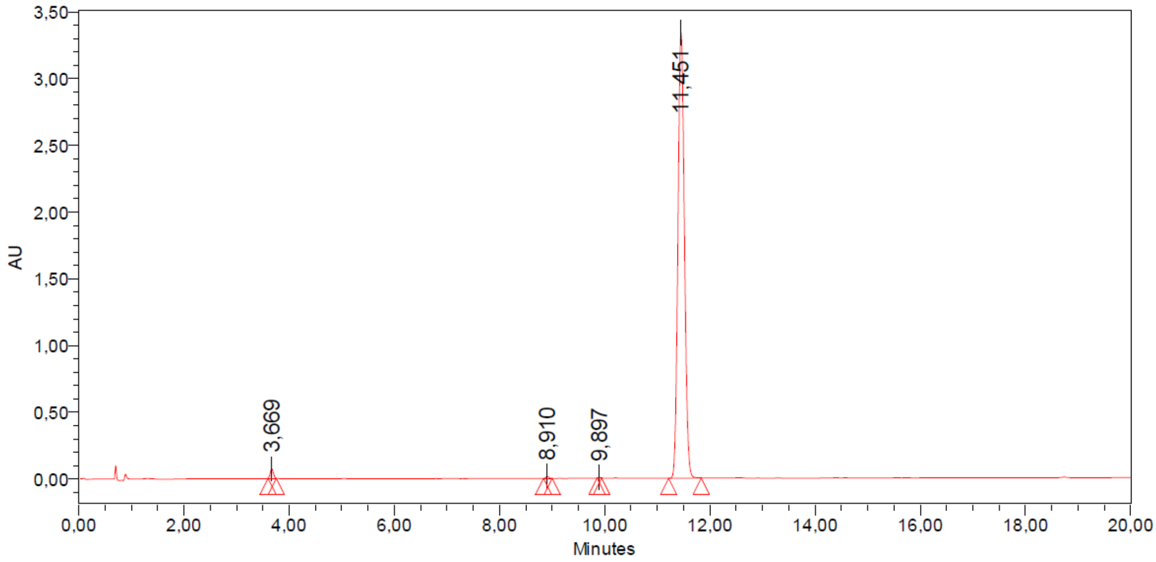

Peak Results

	RT	Area	% Area	Height
1	3,669	295412	1,02	70740
2	8,910	60189	0,21	14447
3	9,897	14162	0,05	4632
4	11,451	28498946	98,72	3338021

SAMPLE INFORMATION

Sample Name:	16i	Acquired By:	System
Sample Type:	Unknown	Sample Set Name:	Analitic
Vial:	83	Acq. Method Set:	5_95%B_20min_P3
Injection #:	1	Processing Method:	Amidas cíclicas
Injection Volume:	7,00 ul	Channel Name:	254+280nm
Run Time:	20,0 Minutes	Proc. Chnl. Descr.:	2998 PDA Calculated: 254+280
Date Acquired:	06/12/2024 17:10:47 CET		
Date Processed:	07/12/2024 14:55:07 CET		

Auto-Scaled Chromatogram

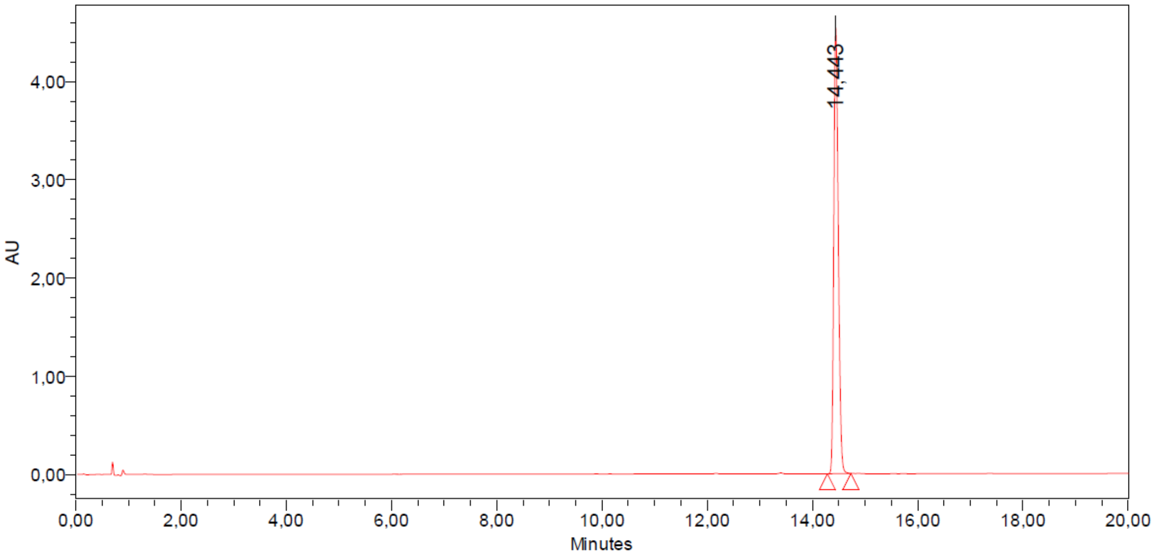

Peak Results

	RT	Area	% Area	Height
1	14,443	27089482	100,00	4540403

SAMPLE INFORMATION

Sample Name:	16e	Acquired By:	System
Sample Type:	Unknown	Sample Set Name:	Analitic
Vial:	92	Acq. Method Set:	5_95%B_20min_P3
Injection #:	1	Processing Method:	Amidas cíclicas
Injection Volume:	5,00 ul	Channel Name:	254+280nm
Run Time:	20,0 Minutes	Proc. Chnl. Descr.:	2998 PDA Calculated: 254+280
Date Acquired:	07/12/2024 13:55:24 CET		
Date Processed:	07/12/2024 14:52:04 CET		

Auto-Scaled Chromatogram

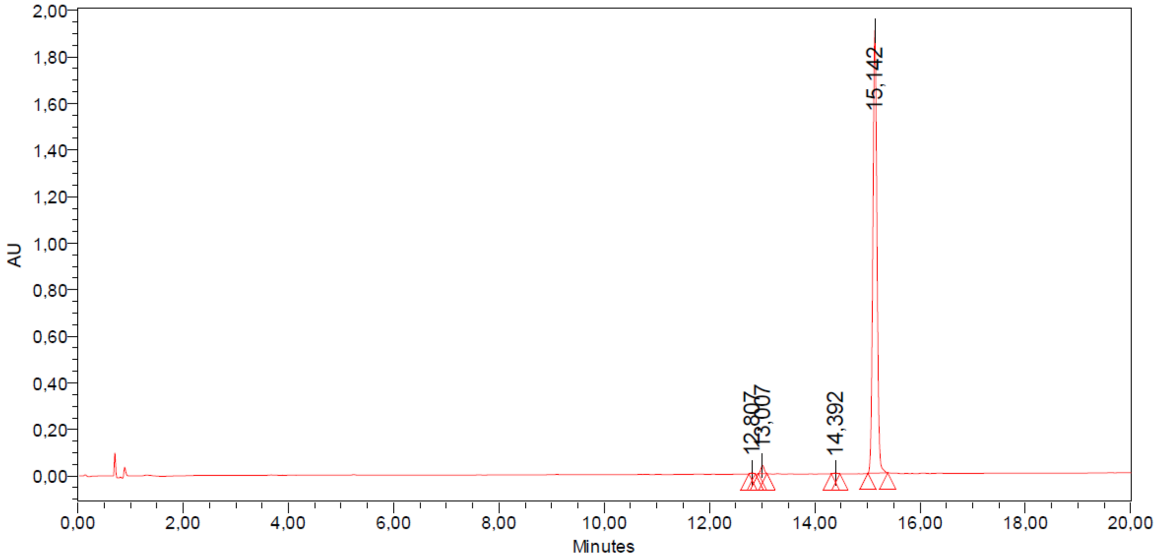

Peak Results

	RT	Area	% Area	Height
1	12,807	19881	0,19	4991
2	13,007	157231	1,49	35040
3	14,392	19287	0,18	4239
4	15,142	10333235	98,13	1901177

SAMPLE INFORMATION

Sample Name:	16p	Acquired By:	System
Sample Type:	Unknown	Sample Set Name:	Analitic
Vial:	84	Acq. Method Set:	5_95%B_20min_P3
Injection #:	1	Processing Method:	Amidas cíclicas
Injection Volume:	15,00 ul	Channel Name:	254+280nm
Run Time:	20,0 Minutes	Proc. Chnl. Descr.:	2998 PDA Calculated: 254+280
Date Acquired:	06/12/2024 13:39:37 CET		
Date Processed:	07/12/2024 14:58:51 CET		

Auto-Scaled Chromatogram

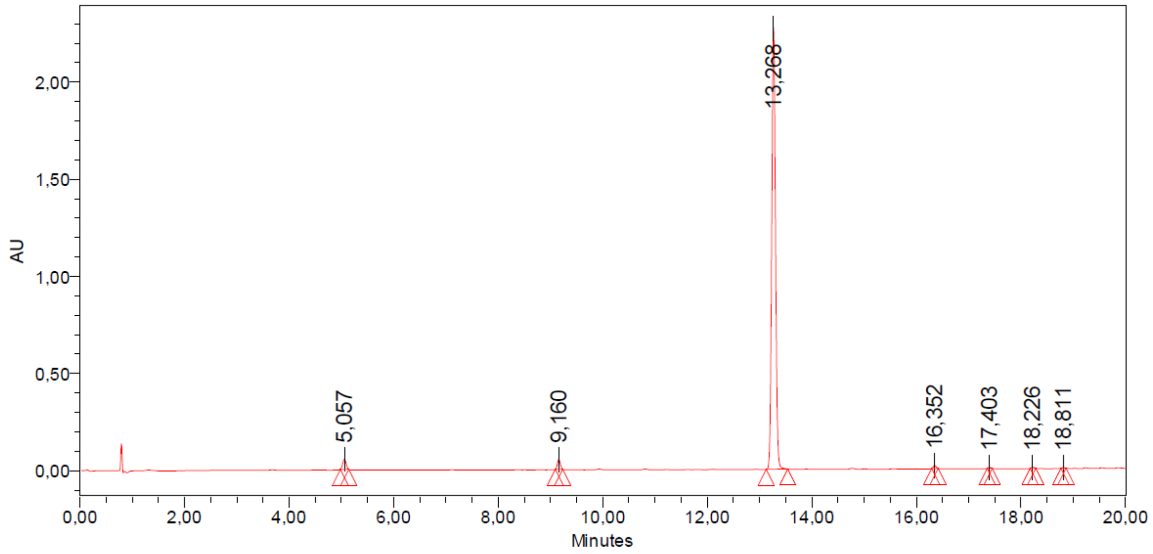

Peak Results

	RT	Area	% Area	Height
1	5,057	244884	2,07	53063
2	9,160	199500	1,68	50569
3	13,268	11291258	95,32	2271281
4	16,352	57140	0,48	14684
5	17,403	16183	0,14	4915
6	18,226	21670	0,18	6077
7	18,811	14762	0,12	4408

SAMPLE INFORMATION

Sample Name:	17b	Acquired By:	System
Sample Type:	Unknown	Sample Set Name:	Analitic
Vial:	93	Acq. Method Set:	5_95%B_20min_P3
Injection #:	1	Processing Method:	Amidas cíclicas
Injection Volume:	5,00 ul	Channel Name:	254+280nm
Run Time:	20,0 Minutes	Proc. Chnl. Descr.:	2998 PDA Calculated: 254+280
Date Acquired:	07/12/2024 14:21:48 CET		
Date Processed:	07/12/2024 15:04:45 CET		

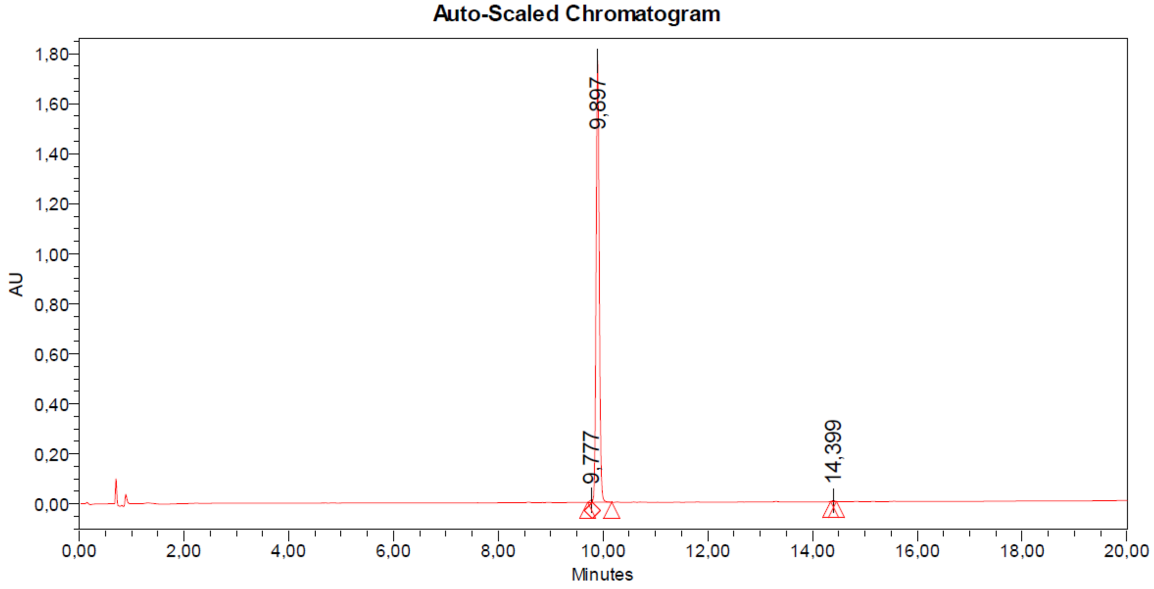

Peak Results

	RT	Area	% Area	Height
1	9,777	28909	0,39	8760
2	9,897	7321530	99,43	1766001
3	14,399	12761	0,17	3433

SAMPLE INFORMATION

Sample Name:	17f	Acquired By:	System
Sample Type:	Unknown	Sample Set Name:	Analitic
Vial:	81	Acq. Method Set:	5_95%B_20min_P3
Injection #:	1	Processing Method:	Amidas cíclicas
Injection Volume:	5,00 ul	Channel Name:	254+280nm
Run Time:	20,0 Minutes	Proc. Chnl. Descr.:	2998 PDA Calculated: 254+280
Date Acquired:	06/12/2024 12:20:45 CET		
Date Processed:	07/12/2024 15:07:41 CET		

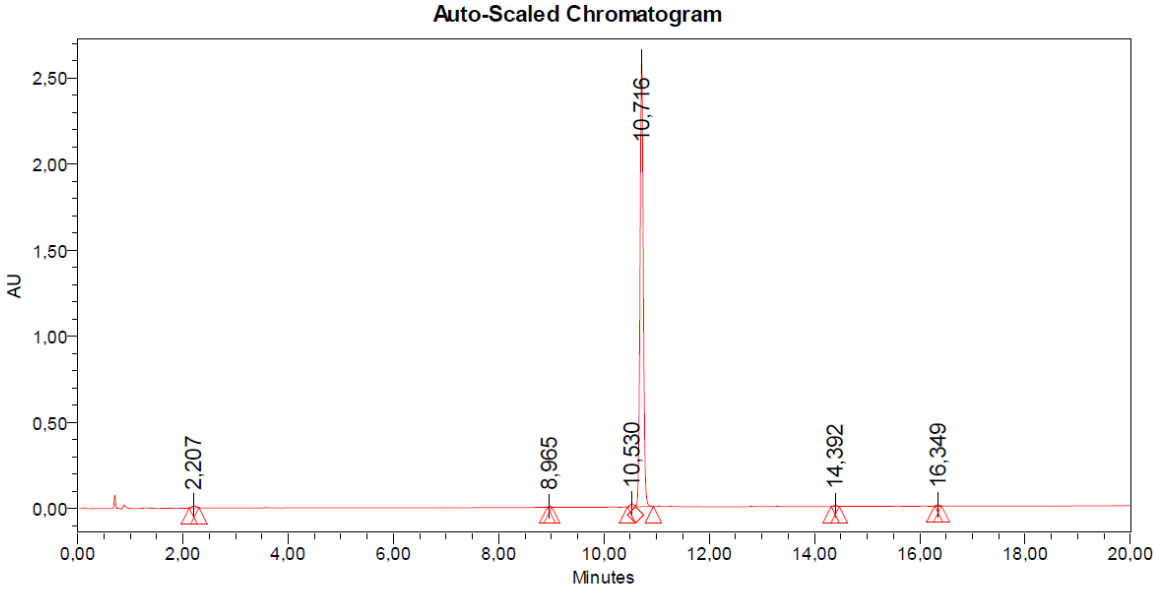

Peak Results

	RT	Area	% Area	Height
1	2,207	66503	0,58	9553
2	8,965	12867	0,11	4551
3	10,530	95700	0,84	17976
4	10,716	11175053	97,97	2587277
5	14,392	27900	0,24	6332
6	16,349	28210	0,25	6460

SAMPLE INFORMATION

Sample Name:	17n	Acquired By:	System
Sample Type:	Unknown	Sample Set Name:	Analitic
Vial:	94	Acq. Method Set:	5_95%B_20min_P3
Injection #:	1	Processing Method:	Amidas cíclicas
Injection Volume:	5,00 ul	Channel Name:	254+280nm
Run Time:	20,0 Minutes	Proc. Chnl. Descr.:	2998 PDA Calculated: 254+280
Date Acquired:	07/12/2024 14:48:07 CET		
Date Processed:	07/12/2024 15:10:32 CET		

Auto-Scaled Chromatogram

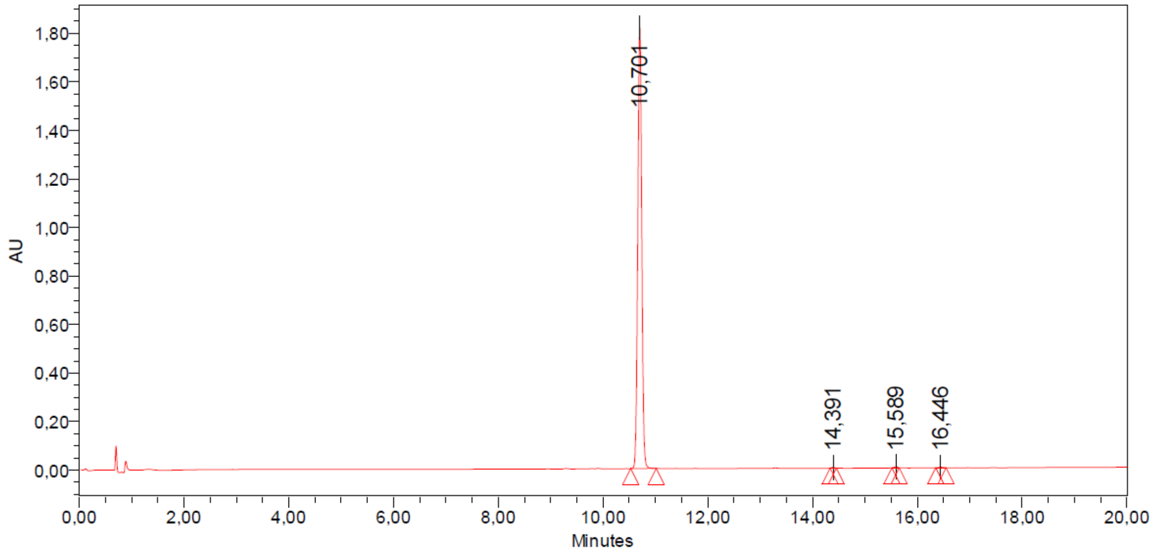

Peak Results

	RT	Area	% Area	Height
1	10,701	9593848	99,58	1818253
2	14,391	10586	0,11	2764
3	15,589	16080	0,17	3393
4	16,446	13602	0,14	2223

SAMPLE INFORMATION

Sample Name:	17o	Acquired By:	System
Sample Type:	Unknown	Sample Set Name:	Analitic
Vial:	83	Acq. Method Set:	5_95%B_20min_P3
Injection #:	1	Processing Method:	Amidas cíclicas
Injection Volume:	20,00 ul	Channel Name:	254+280nm
Run Time:	20,0 Minutes	Proc. Chnl. Descr.:	2998 PDA Calculated: 254+280
Date Acquired:	03/12/2024 17:58:33 CET		
Date Processed:	07/12/2024 15:14:23 CET		

Auto-Scaled Chromatogram

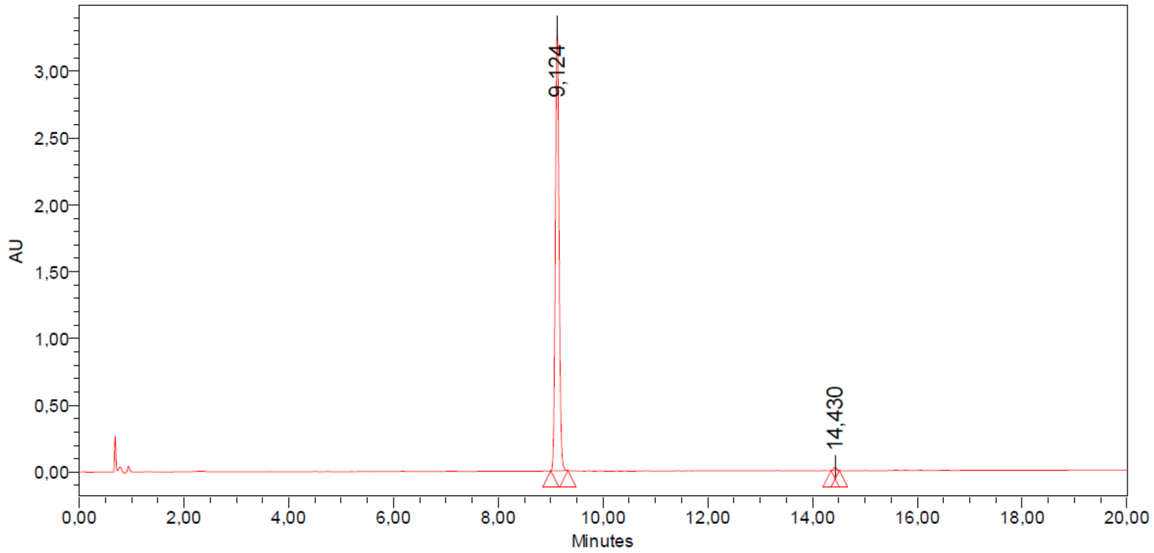

Peak Results

	RT	Area	% Area	Height
1	9,124	16269554	99,35	3316866
2	14,430	106151	0,65	23800

SAMPLE INFORMATION

Sample Name:	18c	Acquired By:	System
Sample Type:	Unknown	Sample Set Name:	Analitic
Vial:	96	Acq. Method Set:	5_95%B_20min_P3
Injection #:	1	Processing Method:	Amidas cíclicas
Injection Volume:	5,00 ul	Channel Name:	254+280nm@1
Run Time:	20,0 Minutes	Proc. Chnl. Descr.:	2998 PDA Calculated: 254+280
Date Acquired:	07/12/2024 15:40:34 CET		
Date Processed:	07/12/2024 16:07:06 CET		

Auto-Scaled Chromatogram

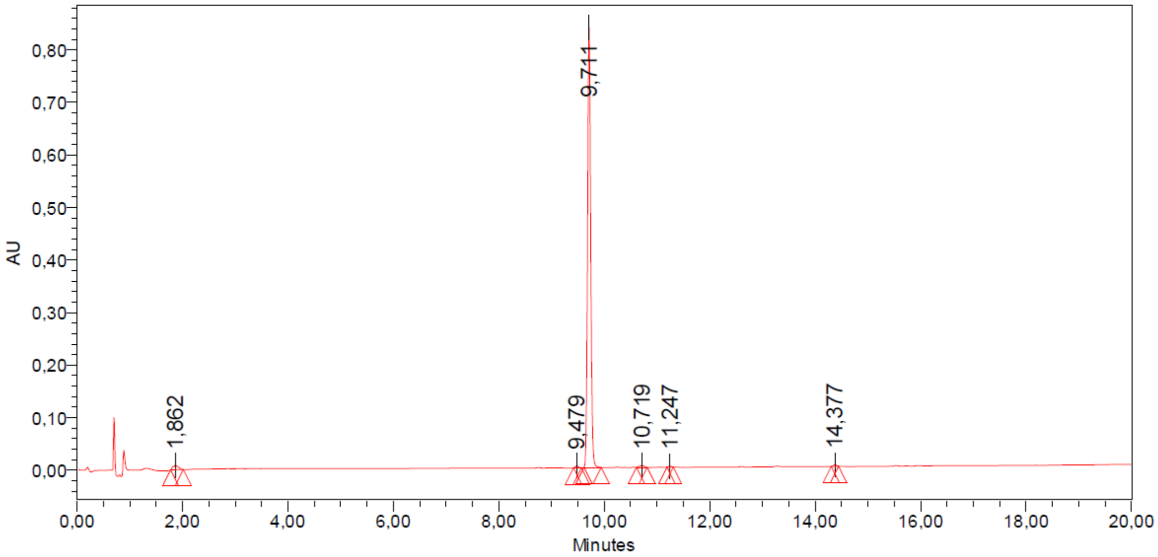

Peak Results

	RT	Area	% Area	Height
1	1,862	59295	1,66	7441
2	9,479	15522	0,43	3869
3	9,711	3458726	96,74	836969
4	10,719	20944	0,59	3078
5	11,247	8167	0,23	2171
6	14,377	12664	0,35	3001

SAMPLE INFORMATION

Sample Name:	18m	Acquired By:	System
Sample Type:	Unknown	Sample Set Name:	Analitic
Vial:	85	Acq. Method Set:	5_95%B_20min_P3
Injection #:	1	Processing Method:	Amidas cíclicas
Injection Volume:	15,00 ul	Channel Name:	254+280nm
Run Time:	20,0 Minutes	Proc. Chnl. Descr.:	2998 PDA Calculated: 254+280
Date Acquired:	06/12/2024 16:44:30 CET		
Date Processed:	07/12/2024 15:17:47 CET		

Auto-Scaled Chromatogram

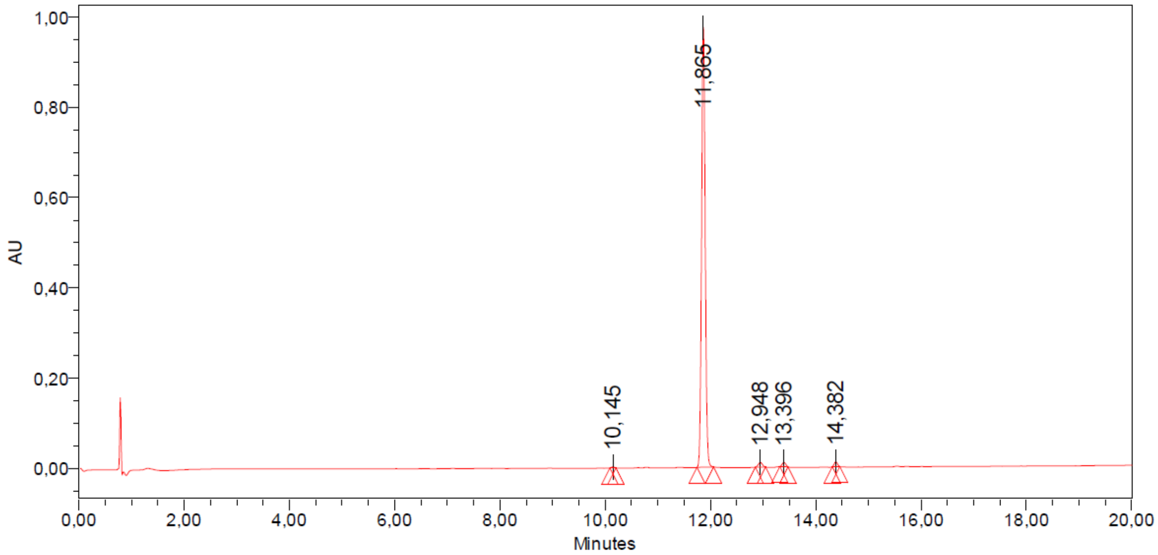

Peak Results

	RT	Area	% Area	Height
1	10,145	8958	0,19	2530
2	11,865	4618353	96,76	974186
3	12,948	57274	1,20	10404
4	13,396	43838	0,92	9491
5	14,382	44701	0,94	10498

SAMPLE INFORMATION

Sample Name:	18o	Acquired By:	System
Sample Type:	Unknown	Sample Set Name:	Analitic
Vial:	86	Acq. Method Set:	5_95%B_20min_P3
Injection #:	1	Processing Method:	Amidas cíclicas
Injection Volume:	15,00 ul	Channel Name:	254+280nm
Run Time:	20,0 Minutes	Proc. Chnl. Descr.:	2998 PDA Calculated: 254+280
Date Acquired:	06/12/2024 14:32:36 CET		
Date Processed:	07/12/2024 15:20:45 CET		

Auto-Scaled Chromatogram

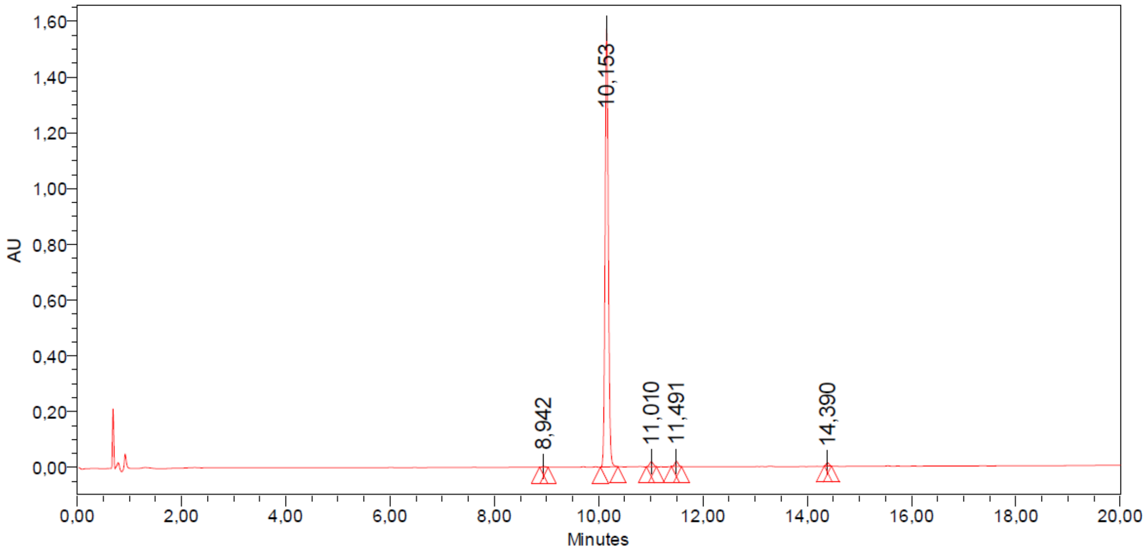

Peak Results

	RT	Area	% Area	Height
1	8,942	12223	0,17	2918
2	10,153	6750348	96,61	1576295
3	11,010	91228	1,31	17879
4	11,491	85594	1,23	18900
5	14,390	47900	0,69	11452

Supplementary Figures

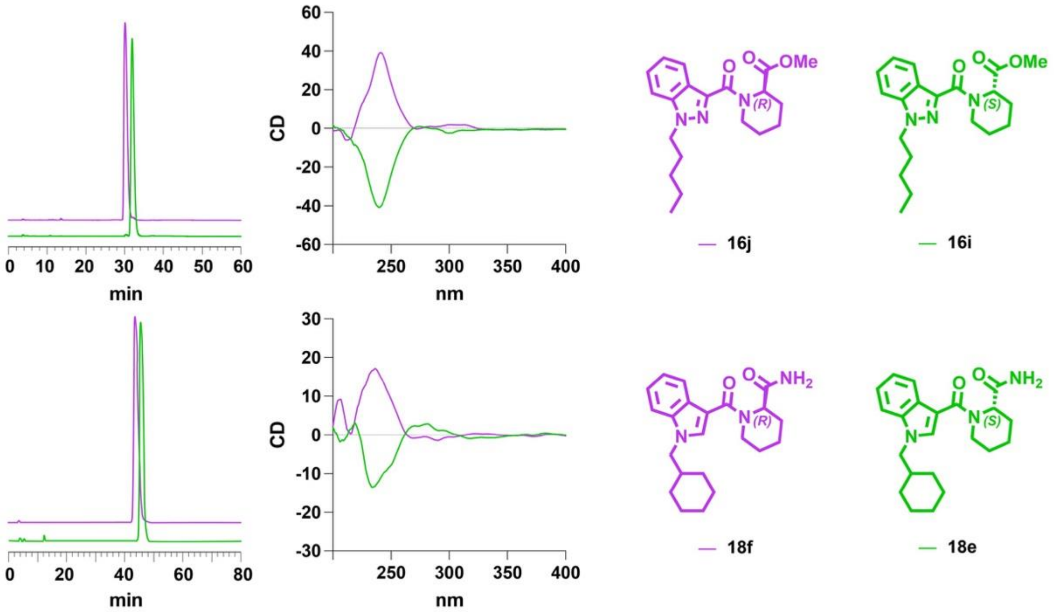

Supplementary Figure S1. HPLC traces of representative enantiopure esters and amides SCRAs (left) and circular dichroism spectra of enantiomer pairs (right). At 240 nm the (*S*) enantiomer showed a negative Cotton effect (green line), while the (*R*) stereoisomer gave a positive Cotton effect (purple line).

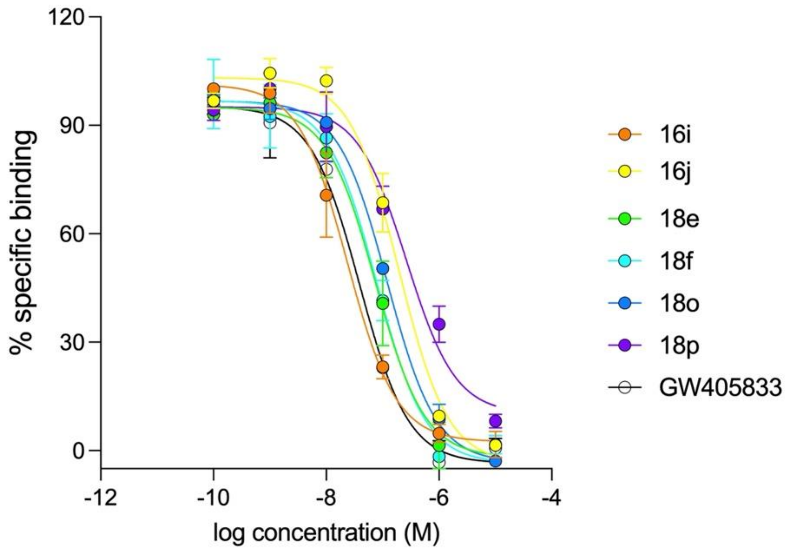

Supplementary Figure S2. Representative dose-response binding curves obtained for selected ligands at CB_2R.

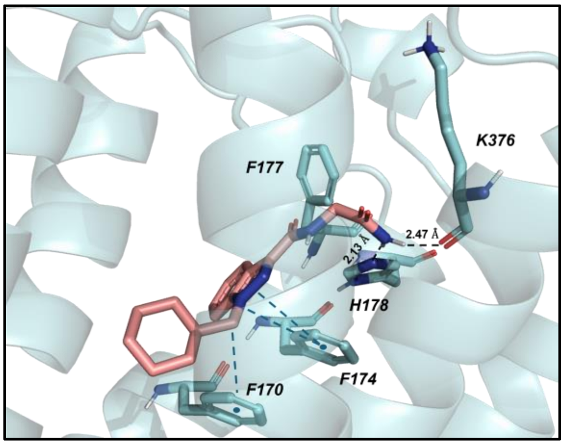

Supplementary Figure S3. Protein-ligand complex returned by MMGBSA calculations performed on the top- scored docking poses of 13 into the CB_1R binding site.

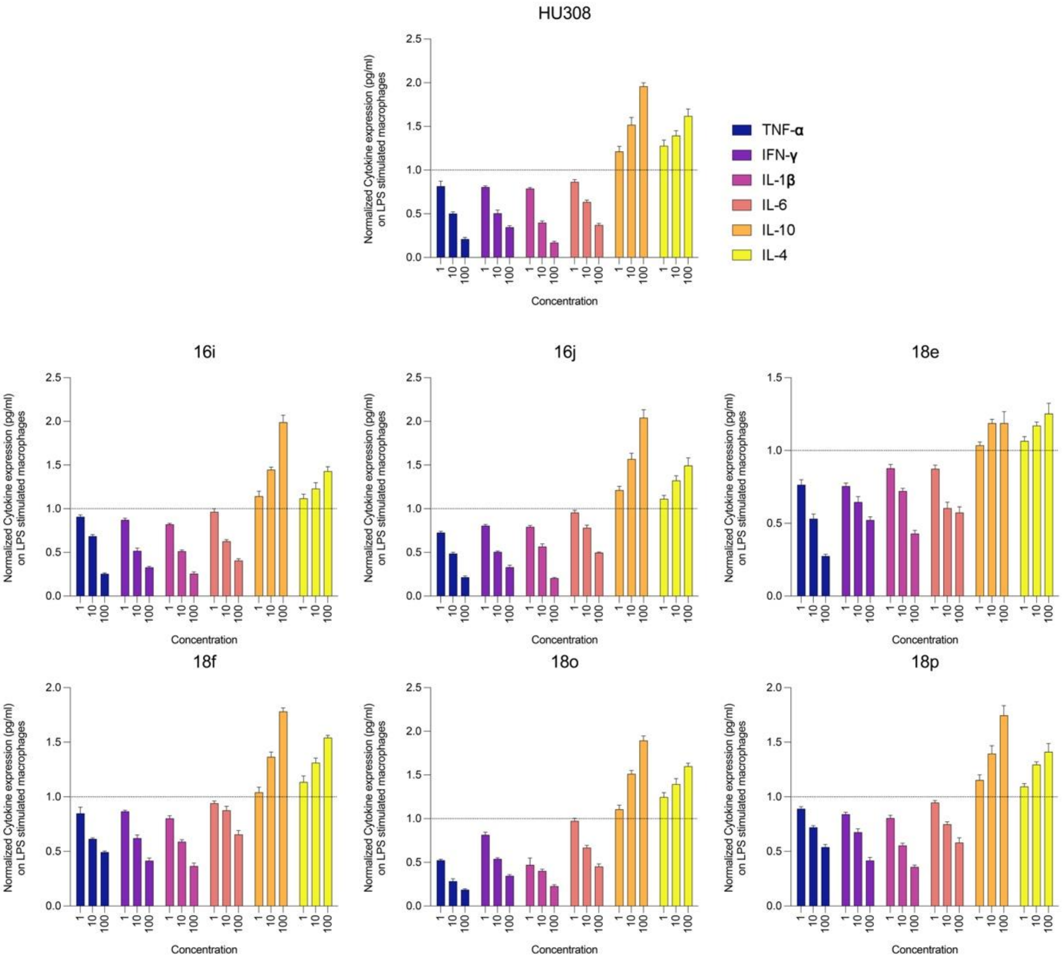

Supplementary Figure S4. Dose-response effects of lead compounds (16i, 16j, 18e, 18f, 18o, and 18p) compared with the agonist HU308 on macrophages stimulated with LPS. Compounds were tested at concentrations of 1 nM, 10 nM, and 100 nM.

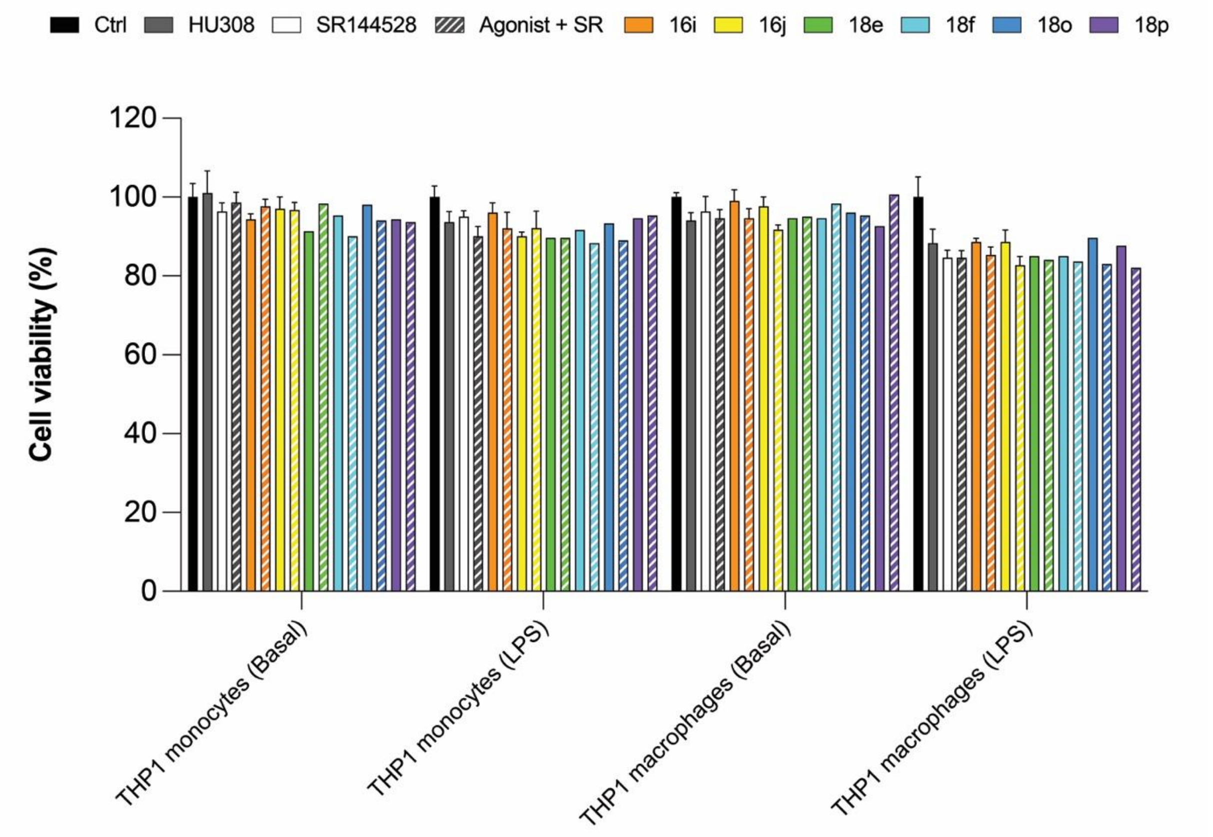

Supplementary Figure S5. Cytotoxicity assessment of lead compounds (16i, 16j, 18e, 18f, 18o, and 18p) on THP1 monocytes and macrophages (n = 3). Cells were treated with each compound at 100 nM under both basal and LPS-stimulated conditions. The viability was compared to controls, including HU308 and SR144528. Results indicate minimal cytotoxic effects, with cell viability remaining close to 100% across all conditions. Error bars represent standard deviation.

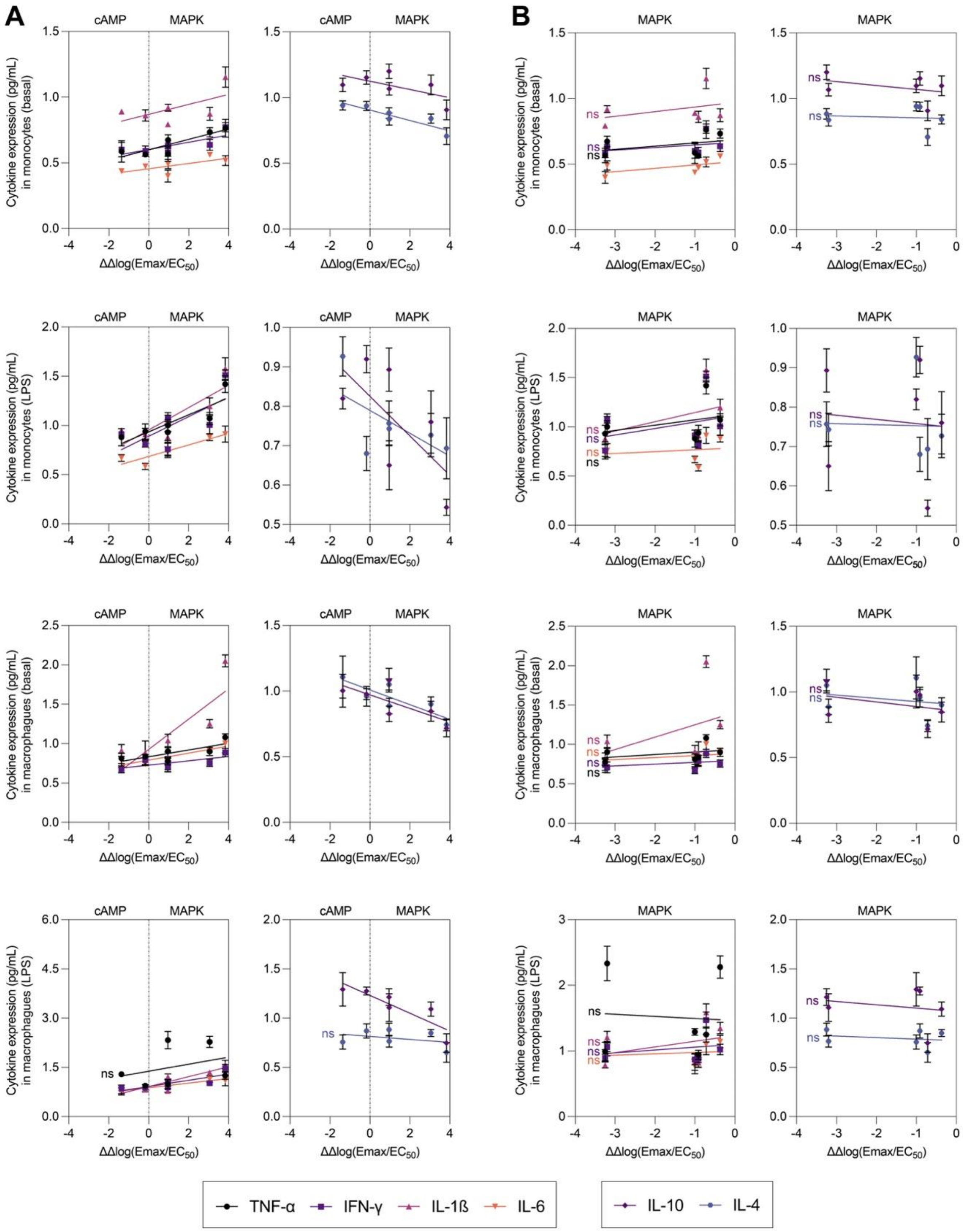

Supplementary Figure S6. Linear correlation between ΔΔlog(Emax/EC50) for cAMP and MAPK pathways and cytokine expression (TNF-α, IFN-γ, IL-1β, IL-6, IL-10, IL-4) in monocytes and macrophages. B) Similar correlation for MAPK and β-arrestin pathways. Slopes showing ns are not significantly different from 0. Graphics are represented in the negative axis as no β-arrestin over MAPK biased ligands were discovered. Data are presented as mean ± SEM.

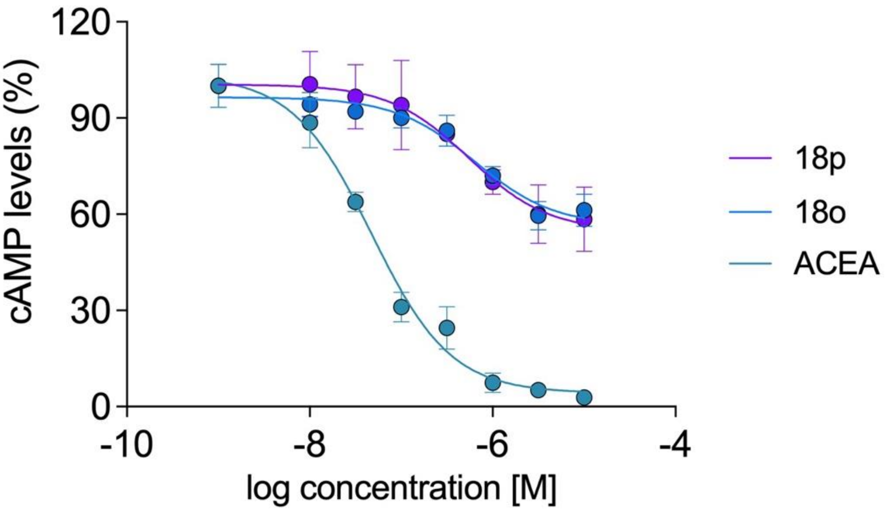

Supplementary Figure S7. Representative dose-response functional curves obtained for selected ligands at CB_1R.

References

(1) Hua, T.; Vemuri, K.; Nikas, S. P.; Laprairie, R. B.; Wu, Y.; Qu, L.; Pu, M.; Korde, A.; Jiang, S.; Ho, J.-H.; Han, G. W.; Ding, K.; Li, X.; Liu, H.; Hanson, M. A.; Zhao, S.; Bohn, L. M.; Makriyannis, A.; Stevens, R. C.; Liu, Z.-J. Crystal Structures of Agonist-Bound Human Cannabinoid Receptor CB1. *Nature* **2017**, *547*, 468–471.

(2) Schrödinger 2024-4: Protein Preparation Wizard; Epik; Prime; LigPrep; Glide, 2024.

(3) ChemAxon. InstantJChem.

(4) Daina, A.; Michielin, O.; Zoete, V. SwissADME: A Free Web Tool to Evaluate Pharmacokinetics, Drug-Likeness and Medicinal Chemistry Friendliness of Small Molecules. *Scientific Reports* **2017**, *7*, 42717.

(5) Daina, A.; Zoete, V. A BOILED-Egg To Predict Gastrointestinal Absorption and Brain Penetration of Small Molecules. *ChemMedChem* **2016**, *11*, 1117–1121.
